# Freezing the Bioactive Conformation to Boost Potency: The Identification of BAY 85-8501, a Selective and Potent Inhibitor of Human Neutrophil Elastase for Pulmonary Diseases

**DOI:** 10.1002/cmdc.201500131

**Published:** 2015-06-17

**Authors:** Franz von Nussbaum, Volkhart M-J Li, Swen Allerheiligen, Sonja Anlauf, Lars Bärfacker, Martin Bechem, Martina Delbeck, Mary F Fitzgerald, Michael Gerisch, Heike Gielen-Haertwig, Helmut Haning, Dagmar Karthaus, Dieter Lang, Klemens Lustig, Daniel Meibom, Joachim Mittendorf, Ulrich Rosentreter, Martina Schäfer, Stefan Schäfer, Jens Schamberger, Leila A Telan, Adrian Tersteegen

**Affiliations:** [a] Medicinal Chemistry Berlin, Bayer HealthCare AG13353 Berlin (Germany); [b] Lead Discovery Wuppertal, Bayer HealthCare AG42096 Wuppertal (Germany); [c] Medicinal Chemistry Wuppertal, Bayer HealthCare AG42096 Wuppertal (Germany); [d] Department of Cardiology Research Wuppertal, Bayer HealthCare AG42096 Wuppertal (Germany); [e] COPD Research, Bayer Healthcare AGSlough SL2 4LY, Berkshire (UK); [f] DMPK Wuppertal, Bayer HealthCare AG42096 Wuppertal (Germany); [g] Lead Discovery, Structural Biology BerlinBayer HealthCare AG, 13353 Berlin (Germany)

**Keywords:** biginelli reaction, biological activity, elastase inhibitors, proteases, pyrimidinones

## Abstract

Human neutrophil elastase (HNE) is a key protease for matrix degradation. High HNE activity is observed in inflammatory diseases. Accordingly, HNE is a potential target for the treatment of pulmonary diseases such as chronic obstructive pulmonary disease (COPD), acute lung injury (ALI), acute respiratory distress syndrome (ARDS), bronchiectasis (BE), and pulmonary hypertension (PH). HNE inhibitors should reestablish the protease–anti-protease balance. By means of medicinal chemistry a novel dihydropyrimidinone lead-structure class was identified. Further chemical optimization yielded orally active compounds with favorable pharmacokinetics such as the chemical probe BAY-678. While maintaining outstanding target selectivity, picomolar potency was achieved by locking the bioactive conformation of these inhibitors with a strategically positioned methyl sulfone substituent. An induced-fit binding mode allowed tight interactions with the S2 and S1 pockets of HNE. BAY 85-8501 ((4*S*)-4-[4-cyano-2-(methylsulfonyl)phenyl]-3,6-dimethyl-2-oxo-1-[3-(trifluoromethyl)phenyl]-1,2,3,4-tetrahydropyrimidine-5-carbonitrile) was shown to be efficacious in a rodent animal model related to ALI. BAY 85-8501 is currently being tested in clinical studies for the treatment of pulmonary diseases.

## Introduction

Human neutrophil elastase (HNE; EC 3.4.21.37) is a member of the chymotrypsin-like family of serine proteases and is stored in the azurophil granules in the neutrophil cytoplasm. This highly active protease is able to break down mechanically important structures of the body’s own cellular matrix (e.g., proteins such as elastin and collagen), as well as proteins foreign to the body (e.g., outer cell wall proteins of Gram-negative bacteria). Furthermore, the enzyme cleaves a variety of endogenous and exogenous proteins, tuning their biological activity such as activation of other bioactive proteases (e.g., matrix metalloproteinases, MMPs), liberation of growth factors, shedding of cell-surface-bound receptors, and degradation of endogenous proteinase inhibitors (e.g., tissue inhibitors of metalloproteases, TIMPs) or exogenous virulence factors.[1] Thus, human neutrophil elastase plays a pivotal role in tissue remodeling processes, as well as in the onset of inflammation and in host defense (innate immune response).

The activity of the versatile protease HNE is tightly controlled by: 1) channeling the potentially dangerous protease to specialized compartments (e.g., storage granula and phagolysosomes), and 2) the presence of extracellular neutralizing endogenous serine protease inhibitors (SERPINs), for example, α-1 antitrypsin (AAT, also known as α-PI) and elafin, which maintain the crucial balance of the protease and its anti-proteases.[2]–[4]

**Figure 1:**
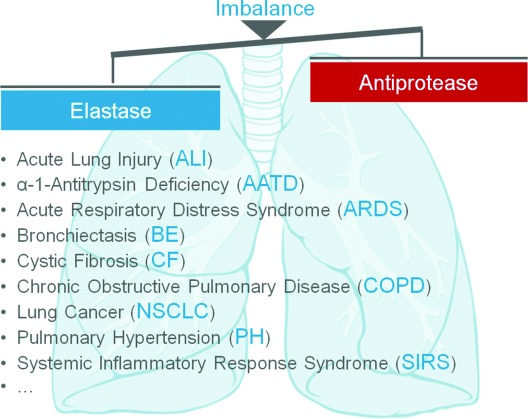
Elastase potentially plays key roles in many pulmonary diseases in which the elastase–anti-protease balance is disrupted. Image: P. J. Lynch, http://www.wikipedia.com.

An imbalance in elastase activity might contribute to the onset and progression of many inflammatory diseases (Figure 1) with an impact on organ tissue integrity, especially in cardiopulmonary diseases, such as chronic obstructive pulmonary disease (COPD), bronchiectasis (BE), pulmonary arterial hypertension (PAH), and pulmonary fibrosis. Elastase knock-out in rodents or anti-protease transgenic animals reveal a significant protection or resistance against experimental lung emphysema,[5] pulmonary hypertension (PH),[6] pulmonary fibrosis,[7] and myocarditis.[8] Individuals with antitrypsin deficiency (AATD) reveal dramatically lower levels of AAT and have an increased risk of suffering from lung emphysema.[9] Notably, elastase knock-out mice are vulnerable to infection with Gram-negative bacteria.[10]

**Figure 2:**
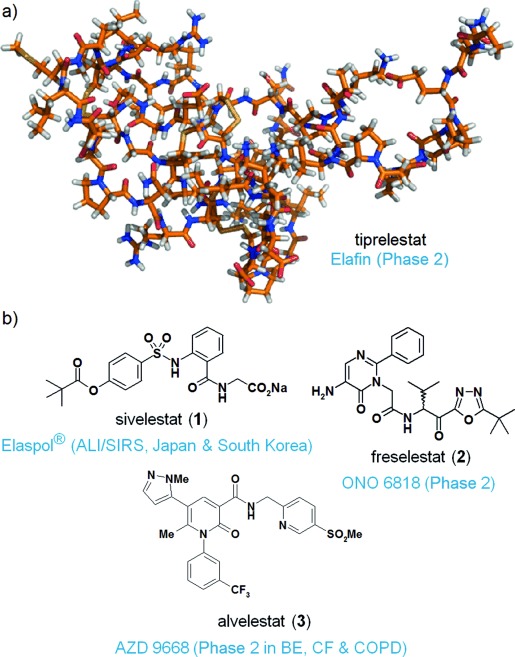
Selection of HNE inhibitors that have reached clinical development. a) Biologicals such as elafin^[11]^ were the first class of HNE-inhibiting drugs. These compounds are only suitable for i.v. application. b) As a second prominent class, covalent drugs that acylate serine in the active site of the enzyme, such as sivelestat (1), or transition-state mimetics, such as freselestat (2), were described. A tremendous effort was required to find the first reversible and selective small-molecule drugs as a third class. A prominent example is alvelestat (3).

So far, various HNE inhibitors have been described;[12], [13] however, only a few chemical entities have had an overall profile suitable for clinical testing. The first potent elastase inhibitors to reach the clinic were biologicals such as elafin[11] (Figure 2). The first small-molecule inhibitors were electrophilic compounds including serine acylators such as sivelestat (**1**)[14]–[16] or transition-state mimetics such as freselestat (**2**).[17] Recently, AstraZeneca reported phase II studies with the reversible HNE inhibitor AZD 9668 (**3**) in patients with pulmonary diseases: Here a small four-week treatment study with 56 cystic fibrosis patients revealed modulation of some secondary endpoint biomarkers (e.g., urinary desmosine concentration), but did not show a significant improvement in primary endpoints (clinical outcomes, e.g., lung function FEV_1_ or quality of life).[18] A further small four-week treatment signal-searching study with 38 BE patients showed a promising improvement in lung function (FEV_1_) and trends for decreases in sputum inflammatory markers.[19] For both indications, longer and larger studies would be needed to confirm the initial findings. In two larger 12-week treatment studies with nearly 1500 COPD patients in total, no clinical benefit and no effect on biomarkers of inflammation or tissue degradation could be demonstrated. This might have been due to the rather short treatment period and the heterogeneity of this disease (a post-hoc analysis revealed an improvement in lung function in a subgroup of patients with a chronic bronchitis phenotype).[20], [21]

In general, combining potency with selectivity is a large hurdle for small-molecule serine protease inhibitors. Herein we report the discovery of a new, highly potent and selective neutrophil elastase inhibitor, BAY 85-8501, with an unprecedented locked bioactive conformation. BAY 85-8501 is currently being investigated in a safety and efficacy trial in BE patients.[22]

## Results and Discussion

In the search for better HNE inhibitors, we performed a high-throughput screen (HTS) of our small-molecule compound library. From the hit list, we identified hexahydroquinoline **4** as the most promising and structurally unique starting point for exploratory chemistry (Table 1). The racemic HTS hit **4** displayed moderate in vitro potency (IC_50_: 0.9 μm). Upon separation of the enantiomers, stereospecific activity became evident as the *R* enantiomer **5** proved to be fivefold more potent (IC_50_: 0.2 μm) than the nearly inactive *S* enantiomer **6** (7 μm). Due to high lipophilicity and a molecular weight >500 Da, the lipophilic binding efficiency[24] (LipE=2.5) of the screening hit was poor (Table 1). Unfortunately, both hexahydroquinolines **5** and **6** showed undesirable inhibition of human CYP 2C9 and/or CYP 3A4.

**Table 1:** Potency, lipophilic binding efficiency, metabolic stability, and CYP inhibition data of the initial screening hit.

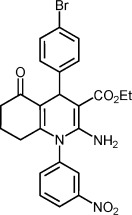					
Compd	Stereo.	HNE IC_50_ [nm][a]	Clog *D*[b]	LipE[c]	CYP 2C9/3A4 IC_50_ [μm][d]
**4**	*rac*	900	–	–	–/–
**5**	*R*	200	4.2	2.5	2/10
**6**	*S*	7000	4.2	1.0	8/0.4

[37] The inhibitory capacity of test compounds was assessed by applying functional biochemical assays with the isolated enzyme (Supporting Information); IC_50_ values were derived from enzyme activity data (pH 7.4) in the presence/absence of various compound concentrations by applying a suitable fluorogenic peptide substrate, MeOSuc-AAPV-AMC.

[b] Clog *D* (pH 7.5) was calculated by using a highly predictive method developed at Bayer, based on data points of experimentally determined log *D* values of internal pharmaceutical compounds and the Simulations Plus p*K*_a_ predictor.[23]

[c] Calculated as LipE=pIC_50_−log *D*.[24]

[d] The capacity of test compounds to inhibit human CYP 2C9 and CYP 3A4 was investigated with pooled human liver microsomes as enzyme source and selective standard substrates (Supporting Information); IC_50_ values were derived from enzyme activity data (pH 7.4) in the presence/absence of various compound concentrations and diclofenac/midazolam as CYP 2C9/CYP 3A4 substrate.

To decrease the molecular weight and ring count, the corresponding ring-opened dihydropyridine analogues **7**–**9** were prepared (Table 2). Fortunately, the ring-opened analogue **7** was fivefold more potent than our screening hit **4**. The potentially unfavorable nitro group of **7** was subsequently converted into a trifluoromethyl group (in **8**) without loss in potency. As a second step toward an even more drug-like molecule, we exchanged the northern bromo substituent for other, potentially more stable, electron-withdrawing groups. The linear cyano substituent of **9** enabled a 10-fold potency increase coupled with a significant improvement in the lipophilic binding efficiency (LipE=4.0, Clog *D*=3.7). Unfortunately, all 2-aminodihydropyridine-type compounds **7**–**9** revealed inhibitory potency toward CYP 2C9 and/or CYP 3A4.

**Table 2:** Initial shift from core bicyclic to monocyclic 2-amino-1,4-dihydropyridine systems: northern and southern SAR.

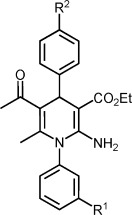
Compd	R^1^	R^2^	HNE IC_50_ [nm][a]	*F*_max_ [%][b]	CYP 2C9/3A4 IC_50_ [μm][c]
**7**	NO_2_	Br	200	15	–/–
**8**	CF_3_	Br	200	31	0.6/0.5
**9**	CF_3_	CN	20	57	0.5/25

[64] The inhibitory capacity of test compounds was assessed by applying functional biochemical assays with the isolated enzyme (Supporting Information); IC_50_ values were derived from enzyme activity data (pH 7.4) in the presence/absence of various compound concentrations by applying a suitable fluorogenic peptide substrate, MeOSuc-AAPV-AMC.

[b] The metabolic stability of test compounds was assessed in the presence of rat hepatocytes by determination of the half-life of the compound (Supporting Information). Clearance parameters and *F*_max_ values were calculated from this half-life, representing a measure of the phase 1 and phase 2 metabolism.

[c] The potency of test compounds to inhibit human CYP 2C9 and CYP 3A4 was investigated with pooled human liver microsomes as enzyme source and selective standard substrates (Supporting Information); IC_50_ values were derived from enzyme activity data (pH 7.4) in the presence/absence of various compound concentrations and diclofenac/midazolam as selective CYP 2C9/CYP 3A4 substrate.

To further explore the central ring system, dihydro-2-pyridones **10**–**13** were pursued next (Table 3). A shift from nitrogen to oxygen at C2 was indeed possible, leading to **10** with an additional stereocenter at C3, which was unfortunately inclined to epimerize. Omitting the ester functionality at C3 streamlined the system to only one stereocenter at C4 (compounds **11**–**13**). In this series, nearly unchanged target potency and overall less inhibitory potency toward CYP 3A4 were observed. A 5-cyano substituent yielded the most potent compound **13**. However, synthetic accessibility and stereochemical integrity were potential limitations of this pyridone class. To tackle these issues, we capitalized on the high SAR flexibility at C3 that allowed another major structural transformation.

**Table 3:** Shift from 2-aminodihydropyridines to dihydro-2-pyridones: eastern and western SAR.

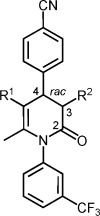
Compd	R^1^	R^2^	HNE IC_50_ [nm][a]	CYP3A4 IC_50_ [μm][b]
**10**	COMe	CO_2_Et *cis*	40	4
**11**	COMe	H	50	9
**12**	CO_2_Et	H	70	–
**13**	CN	H	20	15

[92] The inhibitory capacity of test compounds was assessed by applying functional biochemical assays with the isolated enzyme (Supporting Information); IC_50_ values were derived from enzyme activity data (pH 7.4) in the presence/absence of various compound concentrations by applying a suitable fluorogenic peptide substrate, MeOSuc-AAPV-AMC.

[b] The potency of test compounds to inhibit human CYP 3A4 was investigated with pooled human liver microsomes as enzyme source (Supporting Information); IC_50_ values were derived from enzyme activity data (pH 7.4) in the presence/absence of various compound concentrations and midazolam as CYP 3A4 substrate.

Introduction of a ring nitrogen at position 3 led to 1,4-dihydropyrimidinones, which were especially potent against HNE (Table 4). Enantiopure compounds of this type were readily available by Biginelli chemistry[25] coupled with chiral chromatography (Supporting Information). Impressive flexibility was observed with respect to ligand polarity in the equatorial sphere at N3. Either potentially anionic or cationic compounds, such as carboxylic acids **16** and **19**, or amines **21** and **24**, inhibited HNE with good to excellent potency. Furthermore, an overall decreased inhibitory potency of the 1,4-dihydropyrimidinones against CYP isoforms turned out to be a significant improvement. For example, up to a concentration of 50 μm, amide **23** elicited no observable inhibition toward CYP 2C9 and CYP 3A4. Due to its overall balanced profile with respect to polarity and potency, BAY-678 (**20**) was selected for in-depth in vitro and in vivo characterization as a chemical probe candidate for HNE (see below).

**Table 4:** Potency of 1,4-dihydropyrimidinones: exploration of eastern and western SAR.

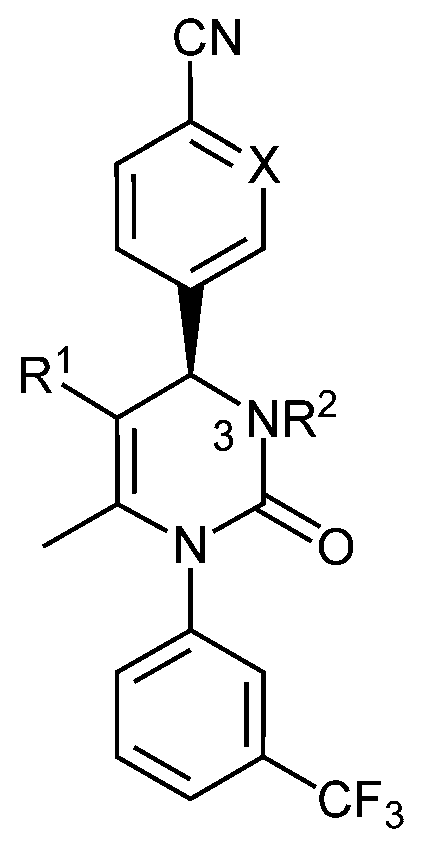
Compd	R^1^	R^2^	X		IC_50_ [nm][a]		*F*_max_ [%][b]	IC_50_ [μm][c]
					HNE	RNE			CYP 2C9/3A4
**14**	CO_2_Et	H	CH		27	192		5	5/>50
**15**	CO_2_Et	CH_2_CONH_2_	CH		1.0	15		13	>50/>50
**16**	CO_2_Et	CH_2_CO_2_H	CH		2.1	43		94	>50/>50
**17**	CO_2_CH_2_CH_2_OH	H	CH		4.3	276		57	2/>50
**18**	COCH_3_	H	CH		13	–		31	7/>50
**19**	COCH_3_	CH_2_CO_2_H	CH		3.5	44		96	31/>100
**20**	COCH_3_	H	N		20	800		57	12/>50
**21**	COCH_3_	CH_2_CH_2_NEt_2_	CH		1	49		9	>50/>50
**22**	CN	H	CH		4.7	1018		35	18/>50
**23**	CN	CH_2_CONH_2_	CH		25	233		77	>50/>50
**24**	CN	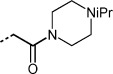	CH		5	126		16	–/–

[193] The inhibitory capacity of test compounds was assessed by applying functional biochemical assays with the isolated (HNE) or enriched (RNE) enzyme (Supporting Information); IC_50_ values were derived from enzyme activity data (pH 7.4) in the presence/absence of various compound concentrations by applying a suitable fluorogenic peptide substrate, MeOSuc-AAPV-AMC.

[b] The metabolic stability of test compounds was assessed in the presence of rat hepatocytes by determination of the half-life of the compound (Supporting Information). Clearance parameters and *F*_max_ values were calculated from this half-life, representing a measure of the phase 1 and phase 2 metabolism.

[c] The potency of test compounds to inhibit human CYP 2C9 and CYP 3A4 was investigated with pooled human liver microsomes as enzyme source and selective standard substrates; IC_50_ values were derived from enzyme activity data (pH 7.4) in the presence/absence of various compound concentrations and diclofenac/midazolam as selective CYP 2C9/CYP 3A4 substrate.

Apparently, all compounds from the 1,4-dihydropyrimidinones series **14**–**24** showed significantly lower potency against rat neutrophil elastase (RNE, Table 4). Obtaining compounds with decent potency not only against HNE but against neutrophil elastase in rodents as well seemed more desirable for upcoming in vivo studies with rats and mice. For this reason we continued our efforts to further optimize potency versus HNE, hoping that this would ultimately lead to compounds with improved RNE inhibition as well.

### X-ray crystallographic investigation of bound ligands

**Figure 3:**
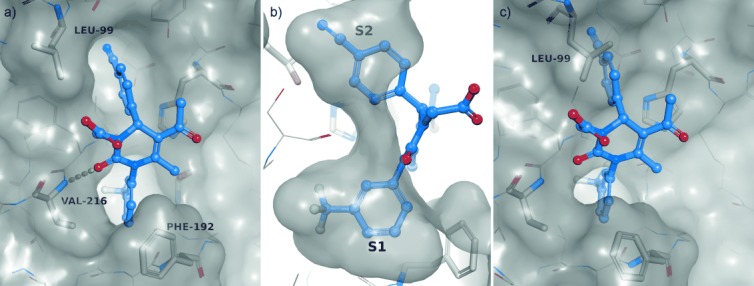
Induced-fit binding mode. Protease (HNE) residues are shown in stick representation (white) with transparent Connolly-like surface.[28], [29] Ligand 19 (purple) is shown in ball-and-stick model (oxygen: red, nitrogen: blue, fluorine: cyan); hydrogen bonds are depicted as broken yellow lines. a) Structure of HNE in complex with 19. Ligand 19 interacts with HNE by a hydrogen bond (3.1 Å) formed between the C2 carbonyl oxygen atom of the central pyrimidine ring and the Val216 backbone amide of HNE. b) Binding to the S1 and S2 subsites is governed by exact protein–ligand shape complementarity of the northern and southern phenyl spheres of 19. c) Binding conformation of 19 overlaid on the binding site of apo-HNE. In the apo structure the S2 subsite next to Leu99 is not large enough to accommodate the northern *para*-cyanophenyl ring. Binding is only possible through an induced-fit mechanism, in which Leu99B is rotated toward the bulk solvent, thus expanding the lipophilic S2 pocket.

Ligand–protein co-crystallization experiments and modeling were applied to design better HNE binders within our 1,4-dihydropyrimidinone series: X-ray crystallography showed the anticipated folding structure for HNE, typical for chymotrypsin-like serine proteases. Upon binding, the equatorial C2 carbonyl moiety of ligand **19** formed a strong hydrogen bond to Val216[26] (Figure 3 a). More significantly, binding of the inhibitor **19** was driven by its shape complementarity with the binding subsites of HNE. The clamp-like ligand **19** fits perfectly into the S1 and S2 subsites of HNE, with both phenyl moieties perpendicularly directed away from the central core (Figure 3 b). In particular, the hydrophobic S1 subsite was fully occupied by the southern *meta*-(trifluoromethyl)phenyl moiety of ligand **19**.

**Figure 4:**
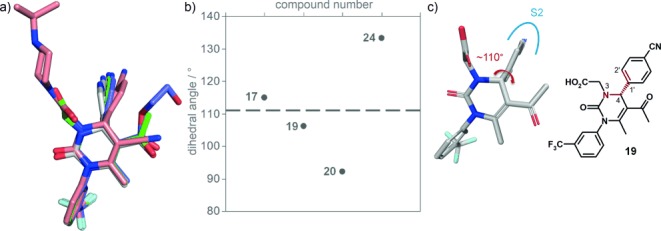
Conformational analysis of HNE-bound ligands based on X-ray crystallographic data (co-crystallization structures). a) Overlay of a series of ligands bound to HNE. b) Rotational dihedral angles observed in this series of crystallized 1,4-dihydropyrimidinones in protein–ligand complexes. See reference [27] for the X-ray data of 17. c) The dihedral angle was measured at the northern cyanophenyl moiety along N3=C4=C1′=C2′ (highlighted in red), as indicated for compound 19.

In contrast, the S2 subsite found in the apo structure of HNE was at best a shallow groove, clearly not providing sufficient room for binding of the northern *para*-cyanophenyl moiety; however, in the ligand-bound structure, Leu99B had been moved toward the bulk solvent, thus expanding the lipophilic S2 to a larger subsite, ideally suited to accommodate the large northern cyanophenyl residue (Figure 3 c). In our complete series of co-crystallization structures we observed an induced-fit binding mode.[27] All bound inhibitors from the dihydropyrimidinone series were characterized by rotational dihedral angles of the northern cyanophenyl moiety of ∼90°–135° (Figure 4) with a preference of dihedral angles of ∼110° (mean: 111.6°).

### Strategy to tune conformational space

**Figure 5:**
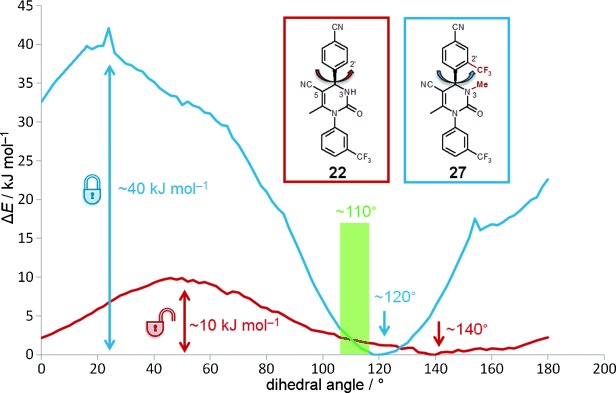
Locking the bioactive conformation with substituents at N3 and C2′. Conformational analysis of free ligands based on modeling. Relaxed coordinate scan of the rotation of the cyanophenyl moiety of 22 and 27 from 0° to 180° in steps of 2°. Depicted is the dihedral angle along N3=C4=C1′=C2′. Whereas the unsubstituted system 22 has very low rotation barriers around the ‘non-ideal’ dihedral angle of 140°, the substituted system 27 is locked with significantly higher rotation barriers at 120°, which is close to the ‘ideal’ dihedral binding angle of 110°. Energies were calculated using the B3LYP/6-31G* density functional technique.[30]

Locking the dihedral angle of the northern phenyl substituent into the preferred bioactive conformation (Figure 4) was envisaged as a design strategy to improve binding efficiency. As a base case, we assessed conformational freedom of the N3- and C2′-unsubstituted system **22** with relaxed scan, density-functional calculations.[30] Rotation appeared to be nearly unhindered, with a barrier of ∼10 kJ mol^−1^ and a lowest-energy conformation at a (suboptimal) dihedral angle of ∼140° (Figure 5). Apparently, this system in its free state was not pre-oriented for HNE binding.

Next, the more hindered N3-methylated and C2′-trifluoromethylated system **27** was analyzed. According to our protein X-ray structures, substitution at C2′ of the northern cyanophenyl sphere (′C2′-north′) was considered to be feasible. This position projected into the solvent and accordingly was not likely to compromise the binding mode; instead, it was well suited to introduce a large rotational barrier at the C4=C1′ axis. Additional substituents at N3 (and C5) of the dihydropyrimidinone core would reinforce that effect by further locking the system at the equatorial–northern interphase. According to our calculations, ligand **27** was expected to have significantly less rotational freedom along the C4=C1′ axis, pre-orienting the system with a rotational barrier of >40 kJ mol^−1^ at a dihedral angle of ∼120°, very close to the ‘ideal’ bioactive dihedral angle of ∼110°.

### Synthesis and assessment of C2′-north substituted systems

The synthesis of C2′-north-substituted systems turned out to be challenging (Table 5). Benzaldehydes with +M substituents at the *ortho* position proved to be less reactive starting materials for the Biginelli reaction. Accordingly, electron-donating substituents had to be avoided at the pyrimidine-forming stage of the synthesis for compounds **25**–**30** (Supporting Information).

**Table 5:** Conformational tuning at N3 and C2′ north: effect on lipophilic binding efficiency.

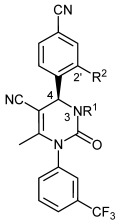
Compd	R^1^	R^2^	HNE IC_50_ [nm][a]	log *D*[b]/Clog *D*[c]	LipE[d]	CYP 2C9/3A4 IC_50_ [μm][e]
**22**	H	H	4.7	2.8[b]	5.6	18/>50
**25**	Me	H	1.6	3.6[c]	5.2	23/40
**26**	H	CF_3_	0.6	3.2[b]	6.0	>50/>50
**27**	Me	CF_3_	0.024	3.7[b]	7.0	–/>20
**28**	H	SO_2_Me	0.540	2.7[b]	7.2	>50/>50
BAY 85-8501 (**29**)	Me	SO_2_Me	0.065	3.0[b]	7.2	>50/>50
**30**	Me	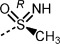	0.250	3.3[c]	6.3	>50/>50

[260] The inhibitory capacity of test compounds was assessed by applying functional biochemical assays with the isolated enzyme (Supporting Information); IC_50_ values were derived from enzyme activity data (pH 7.4) in the presence/absence of various compound concentrations by applying a suitable fluorogenic peptide substrate, MeOSuc-AAPV-AMC.

[b] log *D* (pH 7.5) was determined by reversed-phase HPLC at physiological pH 7.5. A series of standards were injected for which log *D* has already been determined using definitive analytical methods (a homologous series of *n*-alkanones). Plotting of the retention times against their log *D* generated a calibration curve. The retention time of the test compound was then compared with the calibration curve leading to its log *D*.

[c] Clog *D* (pH 7.5) was calculated by using a highly predictive method developed at Bayer, based on data points of experimentally determined log *D* values of internal pharmaceutical compounds and the Simulations Plus p*K*_a_ predictor.[23]

[d] Calculated as LipE=pIC_50_−log *D*.[24]

[e] The potency of test compounds to inhibit human CYP 2C9 and CYP 3A4 was investigated with pooled human liver microsomes as enzyme source and selective standard substrates (Supporting Information); IC_50_ values were derived from enzyme activity data (pH 7.4) in the presence/absence of various compound concentrations and diclofenac/midazolam as selective CYP 2C9/CYP 3A4 substrate.

Whereas N3 alkylation (**22**→**25**) only improved potency twofold, trifluoromethylation at C2′-north (**22**→**26**) advanced the IC_50_ by a factor of eight. Yet, the combination of both substituents at N3 and C2′ (**22**→**27**) boosted potency by more than two orders of magnitude in a synergistic fashion, validating our design hypothesis. The double conformational lock resulted in high lipophilic binding efficiency (LipE=7.0). Still, compound **27** was not an ideal candidate, with log *D*>3 (at pH 7.5). Therefore, we decided to replace the lipophilic trifluoromethyl group by a more polar, less lipophilic alternative while retaining the double conformational lock.

Indeed, with a sulfone group, potency could again be advanced by a factor of ten (**22**→**28**). Combination of the C2′-sulfone with a methyl group at N3 enhanced potency by nearly two orders of magnitude relative to **22**, yielding BAY 85-8501 (**29**, HNE IC_50_: 65 pm) with a formidable lipophilic binding efficiency (LipE 7.2). The C2′-north position also tolerated the slightly basic sulfoximine[31] residue, yielding compound **30** with improved solubility (Table 5). Due to its overall balanced technical profile, BAY 85-8501 (**29**) was selected for in-depth in vitro and in vivo testing (see below).

**Scheme 1:**
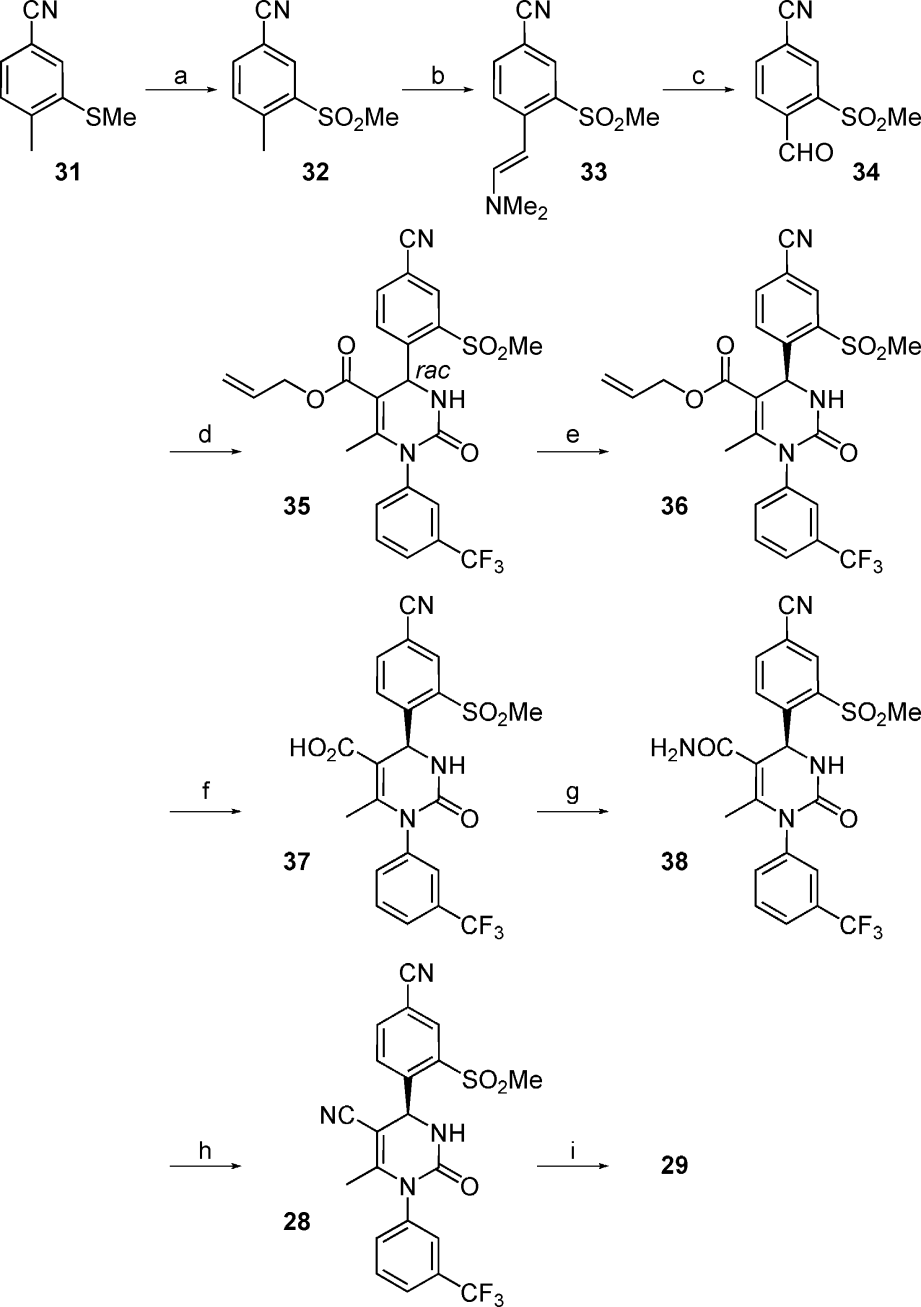
Synthesis of BAY 85-8501 (29). *Reagents and conditions*: a) *m*CPBA, CH_2_Cl_2_, 10 °C→RT, 93 %; b) *N*,*N*-dimethylformamide dimethyl acetal, DMF, 140 °C, 98 % (crude product); c) sodium periodate, H_2_O/THF (1:1), RT, 65 %; d) triethyl phosphate, phosphorus pentoxide, 50 °C, then 1-[3-(trifluoromethyl)phenyl]urea, allyl acetoacetate, reflux, 64 %; e) preparative HPLC, eluent: isohexane/isopropanol (1:1), selector poly(*N*-methacryloyl-D-leucine-dicyclopropylmethylamide), 69 %; f) morpholine, tetrakis(triphenylphosphine)palladium(0) (0.05 equiv), THF, RT, 81 %; g) HATU, DMF, NH_4_Cl, *N*,*N*-diisopropylethylamine, 0 °C→RT, 88 %; h) Burgess reagent [(methoxycarbonylsulfamoyl)triethylammonium hydroxide], THF, RT, 87 %; i) LiHMDS, CH_3_I, THF, −78 °C→RT, 96 %. DMF=*N*,*N*-dimethylformamide; LiHMDS=lithium bis(trimethylsilyl)amide; THF=tetrahydrofuran; HATU=1-[bis(dimethylamino)methylene]-1*H*-1,2,3-triazolo[4,5-*b*]pyridinium 3-oxide hexafluorophosphate.

BAY 85-8501 was synthesized in a nine-step sequence, with deliberate introduction of the electron-withdrawing sulfone substituent prior to the Biginelli reaction in order to increase electrophilicity and reactivity of the corresponding benzaldehyde **34** (Scheme 1). Separation of enantiomers **35** was subsequently achieved by HPLC on chiral phase. The cyano group at the dihydropyrimidinone was installed from carboxylic acid **37** via amide **38** by dehydration with the Burgess reagent.

**Figure 6:**
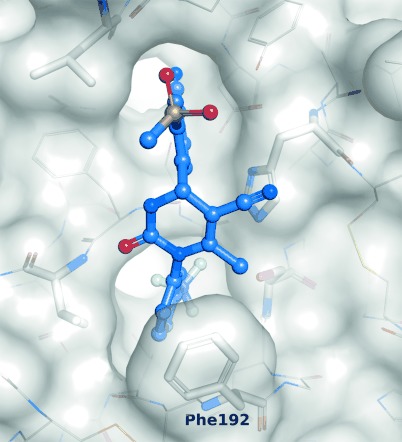
Co-crystallization of 28 with HNE. Protease residues are shown in stick representation with transparent Connolly-like surface; ligand 28 is shown in ball-and-stick representation (oxygen: red, nitrogen: blue, fluorine: cyan, sulfur: yellow). Ligand 28 has a dihedral angle of 109.5° at the northern cyanophenyl moiety along N3=C4=C1′=C2′. The image was generated with PyMOL.[29]

For a better understanding of the binding mode with our novel conformationally locked systems, **28** was co-crystallized with HNE (Figure 6), which revealed a binding mode nearly identical to that of ligand **19** (Figure 3). The N3=C4=C1′=C2′ dihedral angle of 109.5° was very close to the assumed optimum of 110° (Supporting Information). The sulfone moiety pointed outward from the active site while one of its oxygen atoms was hydrogen bonded to a water molecule, gaining further binding energy.

### In-depth in vitro testing of BAY-678 and BAY 85-8501

We ran the biochemical inhibition assay for BAY 85-8501 also in the presence of 1 mm hydrogen peroxide to mimic in vivo conditions of oxidative stress in an inflammatory environment. Under these harsh circumstances, the IC_50_ only shifted by a factor of two toward 140 pm, indicating good oxidative stability.

To confirm the exceptionally high potency of BAY-678 (**20**) and BAY 85-8501 (**29**), we measured enzyme reaction velocities with different substrate concentrations at various inhibitor concentrations and extrapolated the inhibition constants (*K*_i_) from Dixon plots (Supporting Information). Both compounds revealed (substrate) competitive inhibition, further confirming their binding into the active site of the enzyme. However, the *K*_i_ values toward rodent orthologous enzymes were about two orders of magnitude higher than toward HNE (Table 6). Beneficially, BAY-678 and BAY 85-8501 revealed no inhibition against 21 related serine proteases, up to an inhibitor concentration of 30 μm (Table 6).

**Table 6:** Species selectivity: in vitro potency of BAY-678 (20) and BAY 85-8501 (29).

Compd		*K*_i_ [nm][a]		Ser protease
		HNE	RNE	MNE[b]		panel IC_50_ [nm][c]
**20**		15	600	700		>30 000
**29**		0.08	8.0	6.0		>30 000

[286] *K*_i_ values were extrapolated from Dixon plots (Supporting Information). As expected, *K*_i_ values showed good correlation to the IC_50_ values.[24]

[b] Murine neutrophil elastase.

[c] IC_50_ values for 21 related serine proteases, including porcine pancreatic elastase (PPE), were determined by applying functional biochemical assays with the respective isolated enzyme and the appropriate fluorogenic peptide substrate (Supporting Information).

Next we investigated the binding kinetics of BAY 85-8501 (Table 7). BAY 85-8501 showed a long residence time of ∼17 min. According to published data,[33] **29** binds to HNE as rapidly as the endogenous α-proteinase inhibitor (αPI). The latter, however, being a protein, shows a much longer residence time, leading to pseudo-irreversible binding characteristics.

**Table 7:** Enzyme binding kinetics of BAY 85-8501 (29) and the endogenous α-proteinase inhibitor (αPI).

Compd	*k*_on_ [10^6^ m^−1^ s^−1^][a]	*k*_off_ [10^−3^ s^−1^][b]	Residence time [h]
**29**	12.6	1.0	∼0.3
αPI	14.5[c]	0.0002[d]	∼1900[e]

[300] The on-rates at which elastase inhibitors bind to the target were determined by applying a functional biochemical assay using a substrate with a modified fluorescent label, MeOSuc-AAPV-umbelliferyl; this allows very sensitive detection of substrate hydrolysis on the millisecond timescale, in the presence or absence of elastase inhibitor. Using nonlinear regression of the reaction progress curves, the observed rate constant of the onset of inhibition (*k*_obs_) was obtained and plotted against the inhibitor concentration. The slope of the linear regression revealed the estimated *k*_on_ value (Supporting Information).

[b] Calculated from *k*_on_ and *K*_i_ according to the equation *k*_off_=*k*_on_×*K*_i_.

[c] Data from Sinden et al.[33]

[d] Calculated with *K*_i_∼1×10^−14^ m, taken from Beatty et al.[32]

[e] Calculated according to target residence time, 1/*k*_off_.

### Pharmacokinetic studies

Various dihydropyrimidinones showed overall promising pharmacokinetic data in rodents (Table 8). While both early dihydropyrimidinones **17** and **20** revealed medium clearance in rodents as a result of oxidation at C4, the additional electron-withdrawing effect of the sulfone C2′-north substituent in BAY 85-8501 resulted in metabolic stabilization of the drug. BAY 85-8501 (**29**) displayed low clearance and improved half-life in rats, and no inhibitory potency toward CYP isoforms (Tables 5 and 8).

**Table 8:** Pharmacokinetic profile of selected dihydropyrimidinones in rats.[a]

Compd	CL_p_ [L h^−1^ kg^−1^][b]	*V*_SS_ [L kg^−1^][c]	*t*_1/2_ [h]	*F* [%][d]
**17**	2.1	4.7	2.6	43
**20**	2.0	3.9	1.3	83
**29**	0.5	5.8	8.5	63[e]

[322] Mean values were derived by intravenous (0.25–2 h infusion) and oral (gavage) administration of 0.3 mg kg^−1^ in EtOH/PEG400/H_2_O vehicles.

[b] Total plasma clearance.

[c] Apparent steady-state volume of distribution.

[d] Oral bioavailability.

[e] From Tylose (0.5 %) suspension.

### In vivo pharmacodynamic studies: acute lung injury (ALI)

A main role of elastase inhibitors in lung diseases could be the prevention of lung injury driven by chronic inflammation (Figure 1). We set up a rapid, preventive in vivo model that was designed to reflect basic aspects of lung diseases, such as ALI, by combining an exogenous ‘noxa’ [triggered by HNE or porcine pancreatic elastase (PPE)] with an endogenous inflammation that developed over time and was driven by murine neutrophil elastase (MNE).

**Figure 7:**
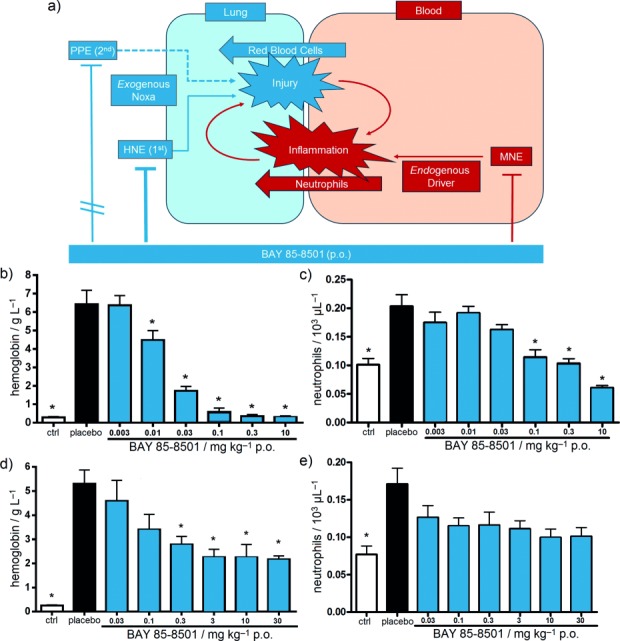
Acute lung injury (ALI) in vivo model in mice. a) Schematic representation of the experimental rationale. 1^st^: HNE-induced ALI model: BAY 85-8501 (29) has a strong inhibitory effect on the exogenous noxa from HNE (*K*_i_ for HNE=0.08 nm), which primarily triggers injury. BAY 85-8501 has only a medium effect on murine neutrophil elastase (MNE; *K*_i_ for MNE=6 nm), which is the primary endogenous driver of inflammation and only a secondary driver of lung injury. 2^nd^: Porcine pancreatic elastase (PPE)-induced ALI model: BAY 85-8501 has no inhibitory effect on the exogenous noxa from PPE, which primarily triggers injury in this second setup. Again, BAY 85-8501 has a medium effect on MNE, which is the primary endogenous driver of inflammation and a secondary driver of injury. b) Hemoglobin concentration after HNE noxa as a primary quantification of injury; data are the mean ±SEM, *n*=4–13, *p<0.05. c) Neutrophil count after HNE noxa as a primary quantification of inflammation; data are the mean ±SEM, *n*=4–13, *p<0.05. d) Hemoglobin concentration after PPE noxa as a primary quantification of injury; data are the mean ±SEM, *n*=4–13, *p<0.05. e) Neutrophil count after PPE noxa as a primary quantification of inflammation; data are the mean ±SEM, *n*=4–13, *p<0.05.

Intratracheal instillation of HNE into the lungs of mice caused severe injury, leading to lung hemorrhage and inflammation that were quantified 1 h after the HNE challenge by measuring hemoglobin concentrations and neutrophil count in the bronchoalveolar lavage fluid (BALF; Figure 7 a, 1^st^). In this model the exogenous HNE noxa was the primary cause of injury and lung hemorrhage. Accordingly, the degree of primary injury was directly dependent on the amount of HNE given. However, the subsequent inflammation that was primarily driven by the endogenous MNE also contributed to secondary injury effects, developing over time. Based on picomolar potency against HNE as well as single digit potency versus MNE, BAY 85-8501 (**29**) completely prevented the development of lung injury and subsequent inflammation when administered 1 h prior to the HNE noxa. In the 0.01 mg kg^−1^ dose group, hemoglobin concentration was already significantly decreased (Figure 7 b). At a dose of 0.1 mg kg^−1^, a significant effect on neutrophil count was observed (Figure 7 c). In this setup, efficacy was predominantly driven by potency against HNE (*K*_i_=0.08 nm).

Next we changed the experimental setup to an exogenous PPE noxa (Figure 7 a, 2^nd^): Intratracheal instillation of PPE caused severe lung hemorrhage and inflammation which were quantified by measuring hemoglobin concentrations and neutrophil count in BALF 1 h after the noxa. As the highly HNE-selective inhibitor BAY 85-8501 had no effect on PPE, BAY 85-8501 could not prevent the primary lung injury in this setup. Nevertheless, BAY 85-8501 could inhibit MNE, the endogenous driver of inflammation and secondary injury, although with decreased potency. Consequently, the effects of BAY 85-8501 on inflammation and secondary injury were weaker at this point, and only observed at 30-fold higher doses (Figure 7 d,e). Efficacy was predominantly driven by potency against MNE (*K*_i_=6 nm) in this second setup. Ultimately, in vitro potency against HNE and MNE translated nicely into in vivo efficacy in our acute lung injury model.

## Conclusions

**Scheme 2:**
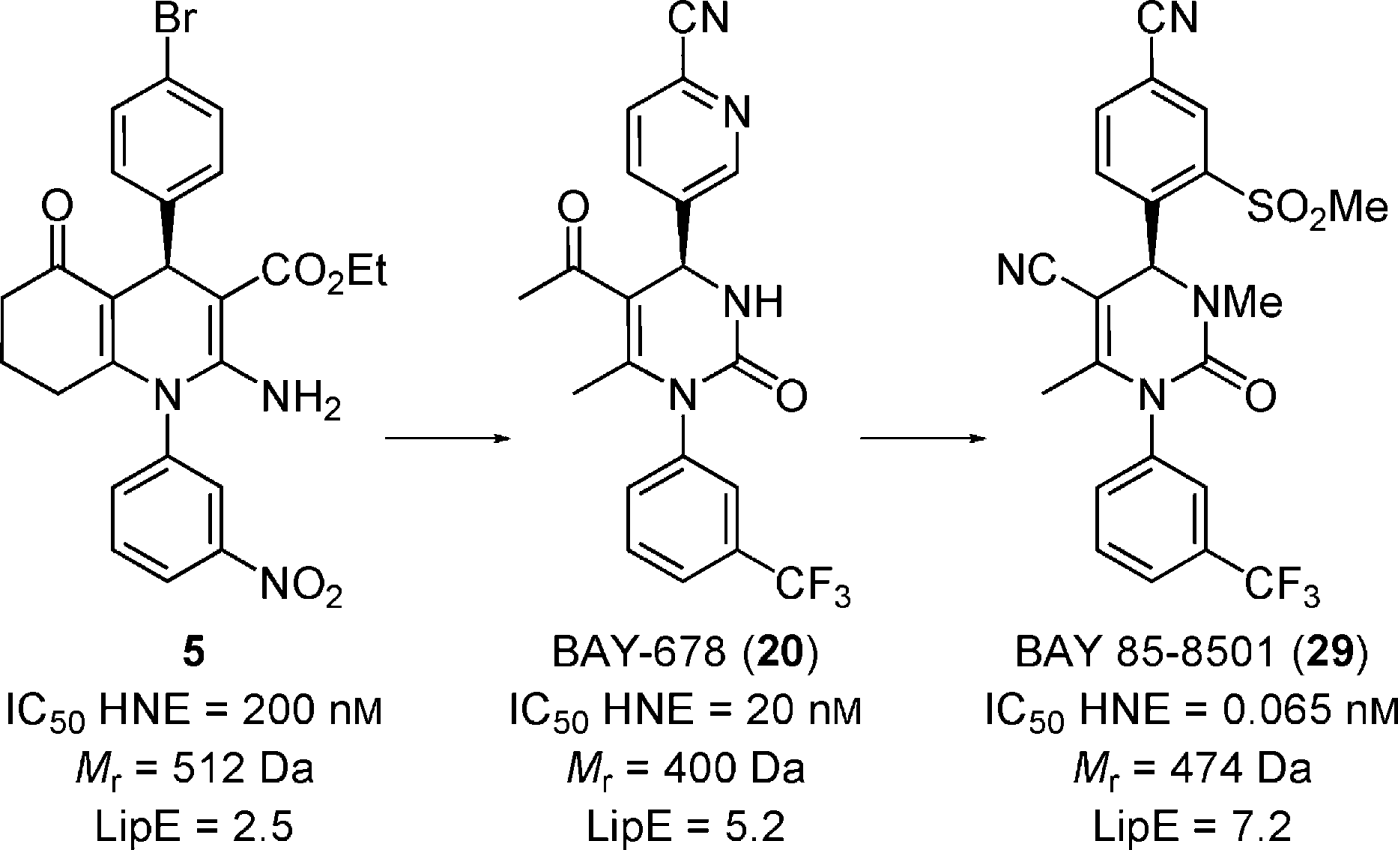
Summary of the optimization path.

In summary, we have evolved a sub-micromolar lipophilic screening hit into a highly selective picomolar HNE inhibitor with lower molecular weight (Scheme 2). Boosting of the lipophilic binding efficiency by nearly five was possible by locking the bioactive conformation of our pyrimidinone lead series on the basis of a thorough conformational understanding of the induced-fit binding mode. Electronic modulation of the northern hemisphere improved in vitro and in vivo pharmacokinetics data significantly. BAY 85-8501 (**29**) has shown in vivo efficacy in various preclinical animal models, results that will be published in due course. BAY 85-8501 is currently in clinical testing for the treatment of pulmonary diseases.

## Experimental Section

**Allyl (*rac*)-4-[4-cyano-2-(methylsulfonyl)phenyl]-6-methyl-2-oxo-1-[3-(trifluoromethyl)phenyl]-1,2,3,4-tetrahydropyrimidine-5-carboxylate (35)**: The reaction was carried out under argon. Triethyl phosphate (22.98 g, 126 mmol) and phosphorus pentoxide (11.94 g, 84.1 mmol) were stirred at 50 °C overnight. The mixture was diluted with *tert*-butyl methyl ether (MTBE; 450 mL). 4-Formyl-3-(methylsulfonyl)benzonitrile (22.00 g, 105 mmol), 1-[3-(trifluoromethyl)phenyl]urea (21.47 g, 105 mmol) and allyl acetoacetate (22.42 g, 158 mmol) were added. The reaction mixture was heated at reflux overnight. MTBE (350 mL) was removed by distillation. The residue was heated at reflux for a further 4 h. The organic solvents were removed. The residue was suspended in Et_2_O and filtered. The solid was washed with H_2_O (350 mL) and Et_2_O (50 mL). Yield: 34.74 g (64 %); ^1^H NMR (400 MHz, [D_6_]DMSO): *δ*=2.15 (s, 3 H), 3.45 (s, 3 H), 4.45 (m, 2 H), 4.95 (d, 1 H), 5.05 (d, 1 H), 5.65 (m, 1 H), 6.40 (d, 1 H), 7.20 (d, 1 H), 7.70 (m, 2 H), 7.80 (m, 1 H), 7.85 (br s, 1 H), 8.10 (br d, 1 H), 8.25 (d, 1 H), 8.35 (s, 1 H) ppm; MS (ESI+) *m*/*z*: 520.2 [*M*+H]^+^.

**Allyl (4*S*)-4-[4-cyano-2-(methylsulfonyl)phenyl]-6-methyl-2-oxo-1-[3-(trifluoromethyl)phenyl]-1,2,3,4-tetrahydropyrimidine-5-carboxylate (36)**: Compound **35** (2.33 g) was separated into enantiomers by preparative chiral-phase HPLC [column: chiral silica gel phase based on the selector poly(*N*-methacryloyl-D-leucine-dicyclopropylmethylamide), 600 mm×30 mm, 10 μm; sample preparation: each 1 g of the sample was dissolved in THF/EtOAc/isohexane (20:25:25, 70 mL); injection volume: 8 mL; eluent: isohexane/isopropanol 1:1; flow: 60 mL min^−1^; *T*: 24 °C; UV detection: *λ*=260 nm]. Yield: 0.8 g (69 %, >99.5 % *ee*); LC–MS (method 14, Supporting Information): *t*_R_=2.11 min; chiral analytical HPLC [column: chiral silica gel phase based on the selector poly(*N*-methacryloyl-D-leucine-dicyclopropylmethylamide), 250 mm×4.6 mm, 5 μm; eluent: isohexane/EtOAc 1:1; flow: 2 mL min^−1^; UV detection: *λ* 260 nm]: *t*_R_=1.45 min; [*α*]_D_^20^=+41.6 (*c*=0.485 in MeOH); ^1^H NMR (400 MHz, [D_6_]DMSO): *δ*=2.10 (s, 3 H), 3.45 (s, 3 H), 4.45 (m, 2 H), 4.95 (d, 1 H), 5.05 (d, 1 H), 5.65 (m, 1 H), 6.40 (d, 1 H), 7.20 (d, 1 H), 7.70 (m, 2 H), 7.80 (m, 1 H), 7.85 (br s, 1 H), 8.10 (br d, 1 H), 8.25 (d, 1 H), 8.35 (s, 1 H) ppm; MS (ESI+) *m*/*z*: 520.1 [*M*+H]^+^; MS (ESI−) *m*/*z*: 518.2 [*M*−H]^−^.

**(4*S*)-4-[4-Cyano-2-(methylsulfonyl)phenyl]-6-methyl-2-oxo-1-[3-(trifluoromethyl)phenyl]-1,2,3,4-tetrahydropyrimidine-5-carboxylic acid (37)**: The reaction was carried out under argon. **36** (800 mg, 1.54 mmol) and morpholine (201 mg, 2.31 mmol, 1.5 equiv) were dissolved in anhydrous THF (25 mL) at room temperature. Tetrakis(triphenylphosphine)palladium(0) (89 mg, 0.077 mmol, 0.05 equiv) was added. The mixture was stirred at room temperature for 90 min, then concentrated. The residue was dissolved in EtOAc (500 mL) and washed with saturated aqueous NH_4_Cl solution (50 mL), H_2_O (50 mL) and brine (50 mL). The organic layer was dried over Na_2_SO_4_ and concentrated. The residue was purified by preparative HPLC (column: GromSil C_18_, 10 μm; eluent: CH_3_CN/H_2_O+0.1 % TFA 10:90→90:10). Yield: 696 mg (94 %); ^1^H NMR (400 MHz, [D_6_]DMSO): *δ*=2.14 (s, 3 H), 3.45 (s, 3 H), 6.35 (d, 1 H), 7.14 (d, 1 H), 7.72 (m, 2 H), 7.80 (m, 1 H), 7.86 (s, 1 H), 8.11 (d, 1 H), 8.27 (d, 1 H), 8.36 (s, 1 H), 12.64 (br s, 1 H) ppm; MS (ESI+) *m*/*z*: 480.1 [*M*+H]^+^; MS (ESI−) *m*/*z*: 478.1 [*M*−H]^−^.

**(4*S*)-4-[4-Cyano-2-(methylsulfonyl)phenyl]-6-methyl-2-oxo-1-[3-(trifluoromethyl)phenyl]-1,2,3,4-tetrahydropyrimidine-5-carboxamide (38)**: The reaction was carried out under argon. **37** (696 mg, 1.45 mmol) and HATU (1.1 g, 2.9 mmol, 2 equiv) were dissolved in anhydrous DMF (35 mL) at 0 °C. After 20 min, NH_4_Cl (388 mg, 7.26 mmol, 5 equiv) and *N*,*N*-diisopropylethylamine (1.31 g, 10.16 mmol, 7 equiv) were added. The mixture was stirred at room temperature for 4 h, and was then concentrated. The residue was purified by preparative HPLC (column: GromSil C_18_, 10 μm; eluent: CH_3_CN/H_2_O+0.1 % TFA 10:90→90:10). Yield: 612 mg (88 %); ^1^H NMR (400 MHz, [D_6_]DMSO): *δ*=1.80 (s, 3 H), 3.40 (s, 3 H), 6.35 (s, 1 H), 7.20 (s, 1 H), 7.25 (br s, 1 H), 7.45 (br s, 1 H), 7.65–7.80 (m, 4 H), 8.10 (d, 1 H), 8.30 (s, 1 H), 8.35 (d, 1 H) ppm; MS (ESI+) *m*/*z*: 479.1 [*M*+H]^+^; MS (ESI−) *m*/*z*: 477 [*M*−H]^−^.

*Method A*: **(4*S*)-4-[4-Cyano-2-(methylsulfonyl)phenyl]-6-methyl-2-oxo-1-[3-(trifluoromethyl)phenyl]-1,2,3,4-tetrahydropyrimidine-5-carbonitrile (28)**: The reaction was carried out under argon. **38** (560 mg, 1.17 mmol) was dissolved in anhydrous THF (35 mL). (Methoxycarbonylsulfamoyl)triethylammonium hydroxide (Burgess reagent; 1115 mg, 4.68 mmol, 4 equiv) was added, and the mixture was stirred at room temperature for 90 min. The solvent was removed in vacuo. The residue was purified by preparative HPLC (column: GromSil C_18_, 10 μm; eluent: CH_3_CN/H_2_O+0.1 % TFA 10:90→90:10). Yield: 470 mg (87 %).

*Method B*: **(4*S*)-4-[4-Cyano-2-(methylsulfonyl)phenyl]-6-methyl-2-oxo-1-[3-(trifluoromethyl)phenyl]-1,2,3,4-tetrahydropyrimidine-5-carbonitrile (28)**: Compound **38** (10.4 g, 21.7 mmol) and trimethylamine (5.63 g, 55.6 mmol) were dissolved in anhydrous THF (50 mL). Trifluoroacetic anhydride (11.69 g, 55.6 mmol) was added dropwise, ensuring a temperature <35 °C. The reaction mixture was stirred at room temperature for 15 min. A saturated aqueous solution of NaHCO_3_ (250 mL) was added dropwise. The mixture was extracted twice with EtOAc. The combined organic layers were washed with brine and dried over MgSO_4_. Silica gel (30 g) was added, and the mixture was concentrated. The residue was purified by column chromatography on silica gel (CH_2_Cl_2_/EtOAc 2:1). Yield: 6.46 g (65 %); mp: 258–259 °C; [*α*]_D_^20^=−222.0 (*c*=0.480 in DMF); ^1^H NMR (400 MHz, [D_6_]DMSO): *δ*=1.80 (s, 3 H), 3.40 (s, 3 H), 6.45 (s, 1 H), 7.70–7.85 (m, 3 H), 7.95 (br s, 1 H), 8.30–8.40 (m, 4 H) ppm; MS (ESI+) *m*/*z*: 461.1 [*M*+H]^+^; MS (ESI−) *m*/*z*: 459.2 [*M*−H]^−^.

*Method A*: **(4*S*)-4-[4-Cyano-2-(methylsulfonyl)phenyl]-3,6-dimethyl-2-oxo-1-[3-(trifluoromethyl)phenyl]-1,2,3,4-tetrahydropyrimidine-5-carbonitrile (BAY 85-8501, 29)**: The reaction was carried out under argon. **28** (75 mg, 163 μmol) was dissolved in THF (2 mL), and NaH suspension in mineral oil (60 %; 9.2 mg, 228 μmol) was added. After stirring at room temperature for 20 min, CH_3_I (32.4 mg, 14.2 μL, 228 μmol) was added. The reaction mixture was stirred at room temperature for 120 min. The mixture was purified directly by preparative HPLC (column: Kromasil-100A C_18_, 250 mm×4.6 mm, 5 μm; eluent: CH_3_CN/H_2_O+0.1 % TFA 10:90→80:20). Yield: 18 mg (23 %).

*Method B*: **(4*S*)-4-[4-Cyano-2-(methylsulfonyl)phenyl]-3,6-dimethyl-2-oxo-1-[3-(trifluoromethyl)phenyl]-1,2,3,4-tetrahydropyrimidine-5-carbonitrile (BAY 85-8501, 29)**: The reaction was carried out under argon. **28** (460.4 mg, 1 mmol) was dissolved in absolute THF (10 mL) and cooled to −78 °C. A solution of LiHMDS (1 m in THF; 1 mL, 1 mmol, 1 equiv) was added dropwise. After stirring for 20 min, CH_3_I (710 mg, 5 mmol, 5 equiv) was added. The mixture was allowed to warm to room temperature over 60 h. The mixture was purified directly by preparative HPLC (column: GromSil C_18_, 10 μm; eluent: CH_3_CN/H_2_O+0.1 % TFA 10:90→90:10). Yield: 454 mg (96 %); ^1^H NMR (400 MHz, [D_6_]DMSO): *δ*=1.80 (s, 3 H), 2.65 (s, 3 H), 3.40 (s, 3 H), 6.45 (s, 1 H), 7.65–8.40 (m, 6 H), 8.45 (s, 1 H) ppm; MS (ESI+) *m*/*z*: 475.0 (100) [*M*+H]^+^; MS (ESI−) *m*/*z*: 473.2 [*M*−H]^−^.

The crystallographic data for the four protein co-crystallization structures with **19**, **20**, **24**, and **28** have been deposited at the RCSB Protein Data Bank (PDB) with the respective access codes 5A09, 5A0A, 5A0B, and 5A0C. All in vivo procedures conformed to European Community directives and national legislation (German law for the protection of animals) for the use of animals for scientific purposes and were approved by the competent regional authority. All other experimental data including chemical procedures and analytics are provided in the Supporting Information.
