# Supplementary material for: Freezing the Bioactive Conformation to Boost Potency: The Identification of BAY 85-8501, a Selective and Potent Inhibitor of Human Neutrophil Elastase for Pulmonary Diseases
Source: ChemMedChem. 2015 Jun 17;10(7):1163–73. doi: 10.1002/cmdc.201500131 (PMC4515084; doi:10.1002/cmdc.201500131)
Supplement: Supplementary file 1 [file cmdc0010-1163-sd1.pdf]

CHEMMEDCHEM
Chemistry & Drug Discovery

Supporting Information

Freezing the Bioactive Conformation to Boost Potency: The Identification of BAY 85-8501, a Selective and Potent Inhibitor of Human Neutrophil Elastase for Pulmonary Diseases

Franz von Nussbaum,*[a] Volkhart M.-J. Li,*[b] Swen Allerheiligen,[c] Sonja Anlauf,[c] Lars Bärfacker,[c] Martin Bechem,[d] Martina Delbeck,[d] Mary F. Fitzgerald,[e] Michael Gerisch,[f] Heike Gielen-Haertwig,[c] Helmut Haning,[c] Dagmar Karthaus,[c] Dieter Lang,[f] Klemens Lustig,[f] Daniel Meibom,[c] Joachim Mittendorf,[c] Ulrich Rosentreter,[c] Martina Schäfer,[g] Stefan Schäfer,[d] Jens Schamberger,[c] Leila A. Telan,[c] and Adrian Tersteegen[b]

cmdc_201500131_sm_miscellaneous_information.pdf

EXPERIMENTAL CHEMISTRY

General Methods: Reaction progress was monitored by HPLC. Crude products were immediately purified using preparative HPLC methodology or flash chromatography on silica gel. The fractions obtained were concentrated in vacuo to remove organic volatiles. *Chemicals* were obtained in analytical grade from Bachem (Switzerland), ABCR (Germany) or Sigma-Aldrich (Germany). All compounds tested in biological assays possessed a purity >95% determined by LC-MS, GC-MS, MS or NMR data.

Mass Spectrometry: The FT-ICR instrument (*HR-FT-ICR-MS*) was an APEX II mass spectrometer (Bruker Daltonics, Billerica, MA, USA) equipped with a 160-mm, room temperature, 7-tesla actively shielded magnet, electrostatic ion-transfer optics, octupole ion-storage device and external off-axis electrospray ion source. *HPLC-FT-ICR-MS* was performed on a HP 1100 HPLC system synchronized to the FT-ICR spectrometer via contact closure. The whole effluent (250 μL/min) was directed to the external electrospray ion source. A heated nitrogen flow of 8 L/min each, with a temperature of 250°C, was used as nebulizing and drying gas. Acurate-mass *TOF-ESI-MS* spectra were obtained on a Micromass LCT mass spectrometer (capillary 3.2 KV, cone 42 V, source 120°C). Samples were injected with a syringe pump (Harvard Apparatus). Leucine enkephalin was used as the standard (resolution 5500). *HR-ESI-MS* spectra were performend using an LTQ Orbitrap mass spectrometer (Thermo Fisher Scientific). All experimental data were acquired using external calibration prior to data acquisition and leucine enkephalin as the standard lock mass. Mass spectroscopic analyses were carried out on full-scan MS using a resolution of 30000.

NMR Spectroscopy: Spectra were recorded on a Bruker Avance DPX200, Bruker Avance DPX300 or Bruker Avance DRX400 instrument with a ^{1}H dual Cryoprobe. Spectra of microsamples were recorded using a 400-MHz 1-mm TXI probe (active volume 5 μL). ^{1}H NMR chemical shifts are given with respect to TMS or the solvent as internal standard ([D$_6$]DMSO: δ H 2.49; CDCl$_3$: δH 7.25. The data were processed using Bruker XWINNMR 3.5 and Topspin 1.3 software. Several NMR spectra were visualized using NPNMR (NPNMR 2.0, NMR processing and visualization software,

2006: www.npnmr.com) or ACD/SpecManager (ACD/labs Release: 12.00, Product Version 12.5, www.acdlabs.com).

Analytical HPLC, LC-MS and GC-MS Methods: *Method 1 (LC-MS):* instrument: Micromass ZQ MS with Waters Alliance 2790 HPLC; column: Uptisphere HDO, 50 mm × 2.0 mm, 3.0 μm; eluent A: H_2O + 0.05% formic acid; eluent B: acetonitrile + 0.05% formic acid; gradient: 0.0 min 5% B → 2.0 min 40% B → 4.5 min 90% B → 5.5 min 90% B; flow: 0.75 mL/min; oven temperature: 45°C; UV detection: 210 nm. *Method 2 (HPLC):* instrument: HP 1100 with DAD detection; column: Kromasil-100 RP-18, 60 mm × 2 mm, 3.5 μm; eluent A: 5 mL $HClO_4$/1 L H_2O; eluent B: acetonitrile; gradient: 0 min 2% B → 0.5 min 2% B → 4.5 min 90% B → 6.5 min 90% B; flow: 0.75 mL/min; temperature: 30°C; UV detection: 210 nm. *Method 3 (LC-MS):* instrument: Micromass Quattro Micro MS with Agilent Series 1100 HPLC; column: Thermo Hypersil GOLD, 20 mm × 4 mm, 3 μm; eluent A: 1 L H_2O + 0.5 mL 50% formic acid; eluent B: 1 L acetonitrile + 0.5 mL 50% formic acid; gradient: 0.0 min 100% A → 3.0 min 10% A → 4.0 min 10% A → 4.01 min 100% A (flow: 2.5 mL/min) → 5.00 min 100% A; flow: 2.0 mL/min; oven temperature: 50°C; UV detection: 210 nm. *Method 4 (HPLC):* instrument: HP 1100 with DAD detection; column: Kromasil-100 RP-18, 60 mm × 2.1 mm, 3.5 μm; eluent A: 5 mL $HClO_4$ (70% strength)/1 L H_2O; eluent B: acetonitrile; gradient: 0 min 2% B → 0.5 min 2% B → 4.5 min 90% B → 9.0 min 90% B → 9.2 min 2% B → 10.0 min 2% B; flow: 0.75 mL/min; temperature: 30°C; UV detection: 210 nm. *Method 5 (LC-MS):* instrument: Micromass Quattro LCZ MS with Agilent Series 1100 HPLC; column: Phenomenex Onyx Monolithic C18, 100 mm × 3 mm; eluent A: 1 L H_2O + 0.5 mL 50% formic acid; eluent B: 1 L acetonitrile + 0.5 mL 50% formic acid; gradient: 0.0 min 90% A → 2.0 min 65% A → 4.5 min 5% A → 6.0 min 5% A; flow: 2 mL/min; oven temperature: 45°C; UV detection: 208–400 nm. *Method 6 (LC-MS):* instrument: Micromass Quattro LCZ MS with Agilent Series 1100 HPLC; column: Phenomenex Synergi Hydro RP Mercury, 20 mm × 4 mm, 2 μm; eluent A: 1 L H_2O + 0.5 mL 50% formic acid; eluent B: 1 L acetonitrile + 0.5 mL 50% formic acid; gradient: 0.0 min 90% A → 2.5 min 30% A → 3 min 5% A → 4.5 min 5% A; flow: 0.0 min 1mL/min → 2.5 min/3.0 min/4.5 min 2 mL/min; oven temperature: 50°C; UV detection: 208–400 nm. *Method 7 (analytical HPLC):* instrument: HP 1050 Series with DAD detection; column: Phenomenex Synergi MAX-RP Mercury, 20 mm × 4 mm, 2 μm; eluent A: 1 L H_2O + 0.5 mL 50%

formic acid; eluent B: 1 L acetonitrile + 0.5 mL 50% formic acid; gradient: 0.0 min 90% A → 2.5 min 30% A → 3 min 5% A → 4.5 min 5% A; flow: 0.0 min 1mL/min → 2.5 min/3.0 min/4.5 min 2 mL/min; oven temperature: 50°C; UV detection: 210 nm. *Method 8 (LC-MS):* instrument: Micromass ZQ MS with Waters Alliance 2795/HP1100 HPLC; column: Phenomenex Synergi Hydro RP Mercury, 20 mm × 4 mm, 2 µm; eluent A: 1 L H_2O + 0.5 mL 50% formic acid; eluent B: 1 L acetonitrile + 0.5 mL 50% formic acid; gradient: 0.0 min 90% A → 2.5 min 30% A → 3.0 min 5% A → 4.5 min 5% A; flow: 0.0 min 1 mL/min → 2.5 min/3.0 min/4.5 min 2 mL/min; oven temperature: 50°C; UV detection: 210 nm. *Method 9 (LC-MS):* instrument: Micromass ZQ MS with HP 1100 Series HPLC and DAD detection; column: Phenomenex Gemini 30 mm × 3.0 mm, 3 µm; eluent A: 1 L H_2O + 0.5 mL 50% formic acid; eluent B: 1 L acetonitrile + 0.5 mL 50% formic acid; gradient: 0.0 min 90% A → 2.5 min 30% A → 3.0 min 5% A → 4.5 min 5% A; flow: 0.0 min 1 mL/min → 2.5 min/3.0 min/4.5 min 2 mL/min; oven temperature: 50°C; UV detection: 210 nm. *Method 10 (HPLC):* instrument: HP 1100 with DAD detection; column: Kromasil-100 RP-18, 60 mm × 2.1 mm, 3.5 µm; eluent A: 5 mL $HClO_4$ (70%)/1 L H_2O; eluent B: acetonitrile; gradient: 0 min 2% B → 0.5 min 2% B → 4.5 min 90% B → 6.5 min 90% B → 6.7 min 2% B → 7.5 min 2% B; flow: 0.75 mL/min; temperature: 30°C; UV detection: 210 nm. *Method 11 (GC-MS):* instrument: Micromass GCT MS with GC 6890; column: Restek RTX-35, 15 m × 200 µm, 0.33 µm; constant flow with helium: 0.88 mL/min; oven temperature: 70°C; inlet: 250°C; gradient: 70°C, 30°C/min → 310°C (3 min remaining). *Method 12 (LC-MS):* instrument: Waters Acquity SQD HPLC System; column: Waters Acquity HSS T3, 50 mm × 1 mm, 1.8 µm; eluent A: 1 L of H_2O + 0.25 mL 99% formic acid; eluent B: 1 L acetonitrile + 0.25 mL 99% formic acid; gradient: 0.0 min 90% A → 1.2 min 5% A → 2.0 min 5% A; flow: 0.40 mL/min; oven temperature: 50°C; UV detection: 210–400 nm. *Method 13 (LC-MS):* instrument: Micromass QuattroPremier MS with Waters Acquity UPLC; column: Thermo Hypersil GOLD, 50 mm × 1 mm, 1.9 µm; eluent A: 1 L H_2O + 0.5 mL 50% formic acid; eluent B: 1 L acetonitrile + 0.5 mL 50% formic acid; gradient: 0.0 min 90% A → 0.1 min 90% A → 1.5 min 10% A → 2.2 min 10% A; flow: 0.33 mL/min; oven temperature: 50°C; UV detection: 210 nm. *Method 14*: instrument: Micromass ZQ with Waters Alliance 2795; column: Phenomenex Synergi 2.5µ MAX-RP 100A Mercury 20 mm × 4 mm; eluent A: 1 L H_2O + 0.5 mL 50% formic acid, Eluent B: 1 L acetonitrile + 0.5 mL 50% formic acid; gradient: 0.0 min 90% A → 0.1 min 90% A → 3.0 min 5% A → 4.0 min

5% A → 4.01 min 90% A; flow: 2 ml/min; oven temperature: 50°C; UV detection: 210 nm.

X-ray Structure Analysis of HNE–Ligand Complexes: Human neutrophil elastase (HNE) was purchased from Serva and was dissolved in 10 mM Hepes buffer at pH 6.5 to a final concentration of 10 mg/mL. Cocrystallization was performed using vapor diffusion in sitting drops at 20°C. Crystals of **19** and **20** were grown in space group *P6(3)* using 28% PEG4000, 0.1 M Tris/HCl pH 8.2 and 0.7 M LiCl with cell axis of $a = b = 72.77$ Å and $c = 69.62$ Å for **19**, and $a = b = 71.71$ Å and $c = 69.58$ Å for **20**. Data of **19** were collected at DESY (BW6) at a wavelength of 1.050 Å at 100 K. Data of **20** were collected using a Rigaku rotating anode generator at a wavelength of 1.54179 Å at 100 K. Crystals were cryoprotected by using 25% glycerol. All data were integrated using XDS[1] and Scala as part of the CCP4 package.[2] Structures were solved by Molecular Replacement using 1PPG as search model. Data of **19** refined to a final R_{value} (R_{free}) of 18.2 (23.9)%. Data of **20** refined to final R_{value} (R_{free}) of 17.0 (20.4)%. Figure S1 shows the crystal structure of **20** in the same orientation and color code as **19** in Figure 3. Crystals of **24** were obtained by adding 2 mM compound to HNE and were crystallized using 15% PEG6000 and 0.2 M ammonium citrate. Crystals were cryoprotected with small amounts of glycerol and data were collected on a Rigaku 007 rotating anode system equipped with an Image Plate detector. Data processing was performed using Mosflm.[3] The structure of **24** was solved using a previously solved complex structure as search model and was refined using the program Refmac in space group *P6(3)22* with $a = 77.5$ Å and $c = 149.6$ Å. The final R_{value} (R_{free}) converged to 17.27 (21.81)%. Crystals of **28** were grown by adding 2 mM compound originally dissolved to 100 mM in DMSO in space group *P3(2)*, and grew in a 1:1 ratio using 0.1 M MES at pH 6.5 and 1.2 M sodium malonate. The crystals were cryoprotected with 25% glycerol and data for both compounds were collected on a Bruker rotating anode system equipped with a CCD detector. Data of **28** were integrated and scaled with the Proteum software package.[4] Data were solved using the program Molrep, using a previously solved complex structure as search model. Figure S2 shows the crystal structure of **24** in the same orientation and color code as **19** in Figure 3. The structure of **28** was refined using the program Refmac in the CCP4 suite in the trigonal space group *P3(2)* with $a = b = 71.5$ Å and $c = 97.4$ Å, with two independent protein inhibitor complexes in the asymmetric unit (Figure S3). Data refined to a final R_{value} (R_{free}) of 16.06 (22.13)%. The absolute

structures of **19**, **20**, **24** and **28** at the pyrimidine carbon atom were determined without any doubt in all complex X-ray structures. The crystallographic data for the four structures have been deposited with the RCSB Protein Data Bank (PDB) with access codes 5a09, 5a0a, 5a0b, 5a0c.

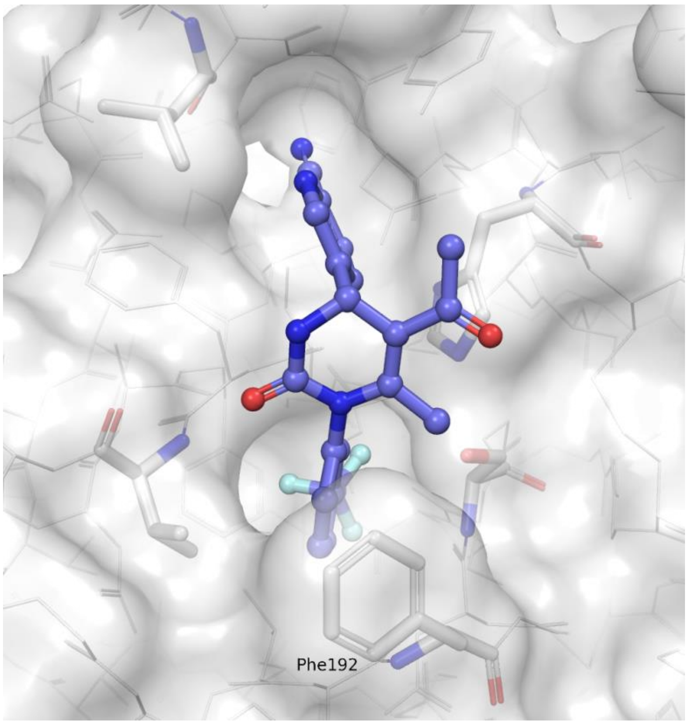

Figure S1. Crystal structure of HNE in complex with compound **20**: The protease is shown in a stick representation (white) with transparent Connolly-like surface; ligand **20** (purple) is shown as balls and sticks. Heteroatoms are colored as follows: oxygen, red; nitrogen, blue; fluorine, cyan.

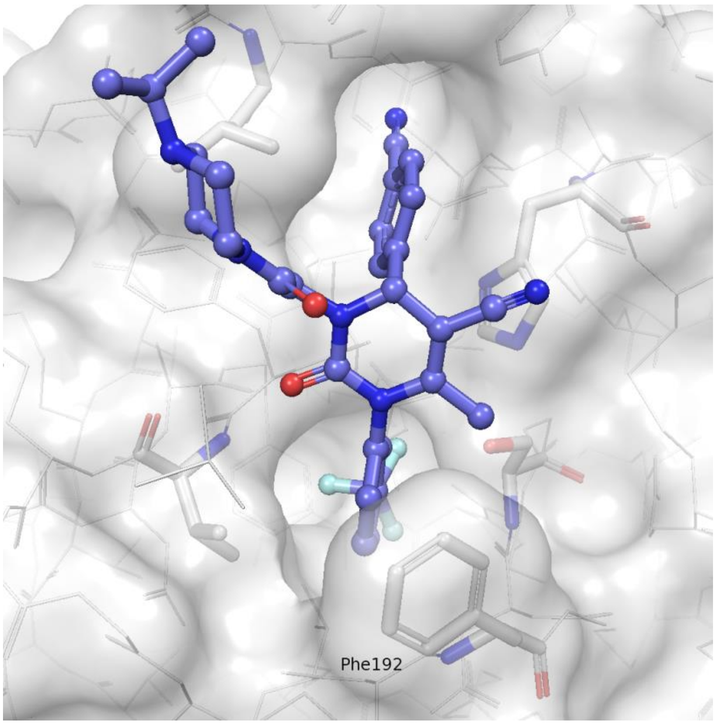

Figure S2. Crystal structure of HNE in complex with compound **24**: Color coding is identical to that in Figure S1.

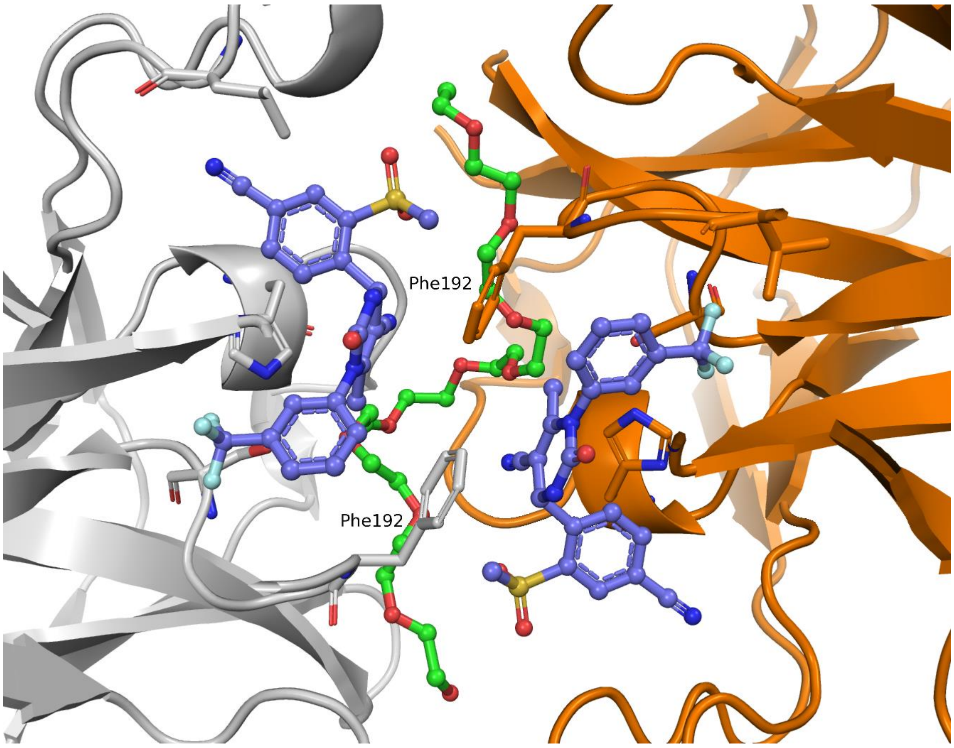

Figure S3. Cocrystallization of **28** with HNE. In the crystal structure two independent molecules were found in the asymmetric unit related by non-crystallographic twofold symmetry; Phe192 is packed toward the *meta*-(trifluoromethyl)phenyl moiety of **28**, as seen in the X-ray structure of HNE in complex with **19**, but in addition it is also packed toward the central pyrimidine as well as the methyl of the

sulfone of **28** in the neighboring molecule. This 'glue' effect resulted in a shift of Phe192 by around 1.1 Å away from its position compared to the cocrystal with **19**. In addition, two MES molecules (omitted from the figure for clarity) and one PEG molecule were localized in the electron density, stabilizing the packing. The nitrogen atom of the pyrimidine core was hydrogen-bonded to the solvent MES molecule. PEG = polyethylene glycol, MES = 2-(*N*-morpholino)ethanesulfonic acid.

Small Molecule X-ray Structures: The crystallographic data of **29** and **30**, as well as figures depicting the thermal ellipsoids of the structures, are shown in Tables S1 and S2, and Figures S4 and S5.

Colorless crystals of compound **29** were obtained from slow evaporation of a methanol solution of the compound at room temperature. A single crystal was mounted on a cryoloop using a protective oil. Single-crystal X-ray diffraction data were collected at 100 K (**29**) on a Bruker Proteum system equipped with a CCD area detector and Cu X-ray radiation (Cu Kα, λ = 1.54178 Å). X-ray data collection and processing of data was performed using the Proteum software package.[4] SHELXS was used for structure solution and SHELXL was used for full-matrix least-squares refinement on F^2.[5] All non-hydrogen atoms were refined anisotropically. All hydrogen atoms were added in calculated positions and refined riding on their resident atoms. The stereochemistry could be assigned unambiguously for both structures. The program XP in the Proteum software package was used for molecular representations.

Colorless crystals of compound **30** were obtained from slow evaporation of an ethanol solution of the compound. A single crystal was mounted on a cryoloop using a protective oil. Single-crystal X-ray diffraction data were collected at 100 K on an Oxford Diffraction (Xcalibur series) system equipped with a CCD area detector with sealed-tube Cu X-ray radiation (Cu Kα, λ = 1.54178 Å). X-ray data collection and processing of data was performed using Crysalis.[6] SHELXS was used for structure solution and SHELXL was used for full-matrix least-squares refinement on F^2.[5] There are two molecues present in the asymmetric unit. All non-hydrogen atoms were refined anisotropically. All hydrogen atoms were added in calculated positions and refined riding on their resident atoms. The stereochemistry could be assigned unambiguously. The program XP in the Proteum software package was used for molecular representations.

The crystallographic data for the two structures have been deposited with the Cambridge Chrystallographic Data Centre (CCDC) with deposition codes CCDC 1060270 and CCDC 1060271

Table S1. Crystallographic data for BAY 85-8501 (**29**).

Empirical formula	$C_{22}H_{17}F_3N_4O_3S + H_2O$
Formula weight	474.46 + H_2O
Temperature	100(2) K
Wavelength	1.54178 Å
Crystal system	Monoclinic
Space group	*P2(1)*
Unit cell dimensions	*a* = 12.3534(5) Å, α = 90°
	b = 11.2949(5) Å, β = 93.794(2)°
	c = 15.8155(6) Å, γ = 90°
Volume	2201.91(16) $Å^3$
Z	4
Density (calculated)	1.455 mg/m^3
Absorption coefficient	1.855 mm^{-1}
F(000)	992
Crystal size	0.15 × 0.10 × 0.02 mm^3
Reflections collected	46352
Independent reflections	5149 [R_{int} = 0.0541]
Data / restraints / parameters	5149 / 575 / 610
Goodness-of-fit on F^2	1.042
Final *R* indices [I > 2σ(I)]	R_1 = 0.0341, wR_2 = 0.0832
R indices (all data)	R_1 = 0.0390, wR_2 = 0.0852
Absolute structure parameter	0.010(14)
Largest diff. peak and hole	0.362 and –0.288 e.$Å^{-3}$

Table S2. Crystallographic data for **30**.

Empirical formula	$C_{22}H_{18}F_3N_5O_2S$ + 0.56 MeOH
Formula weight	491.34
Temperature	100 K
Wavelength	1.54178 Å
Crystal system	Orthorhombic
Space group	*P2(1)2(1)2(1)*
Unit cell dimensions	*a* = 12.2019(2) Å, α = 90°
	b = 12.8296(3) Å, β = 90°
	c = 14.4797(3) Å, γ = 90°
Volume	2266.73(8) $Å^3$
Z	4
Density (calculated)	1.440 mg/m^3
Absorption coefficient	1.791 mm^{-1}
F(000)	1016
Crystal size	0.20 × 0.04 × 0.03 mm^3
Reflections collected	14873
Independent reflections	3991 [R_{int} = 0.0402]
Completeness	98.3%
Data / restraints / parameters	3991 / 0 / 323
Goodness-of-fit on F^2	1.097
Final R indices [I > 2σ(I)]	R_1 = 0.0607, wR_2 = 0.1654
R indices (all data)	R_1 = 0.0639, wR_2 = 0.1681
Absolute structure parameter	0.00(3)
Largest diff. peak and hole	1.054 and –0.606 e.$Å^{-3}$

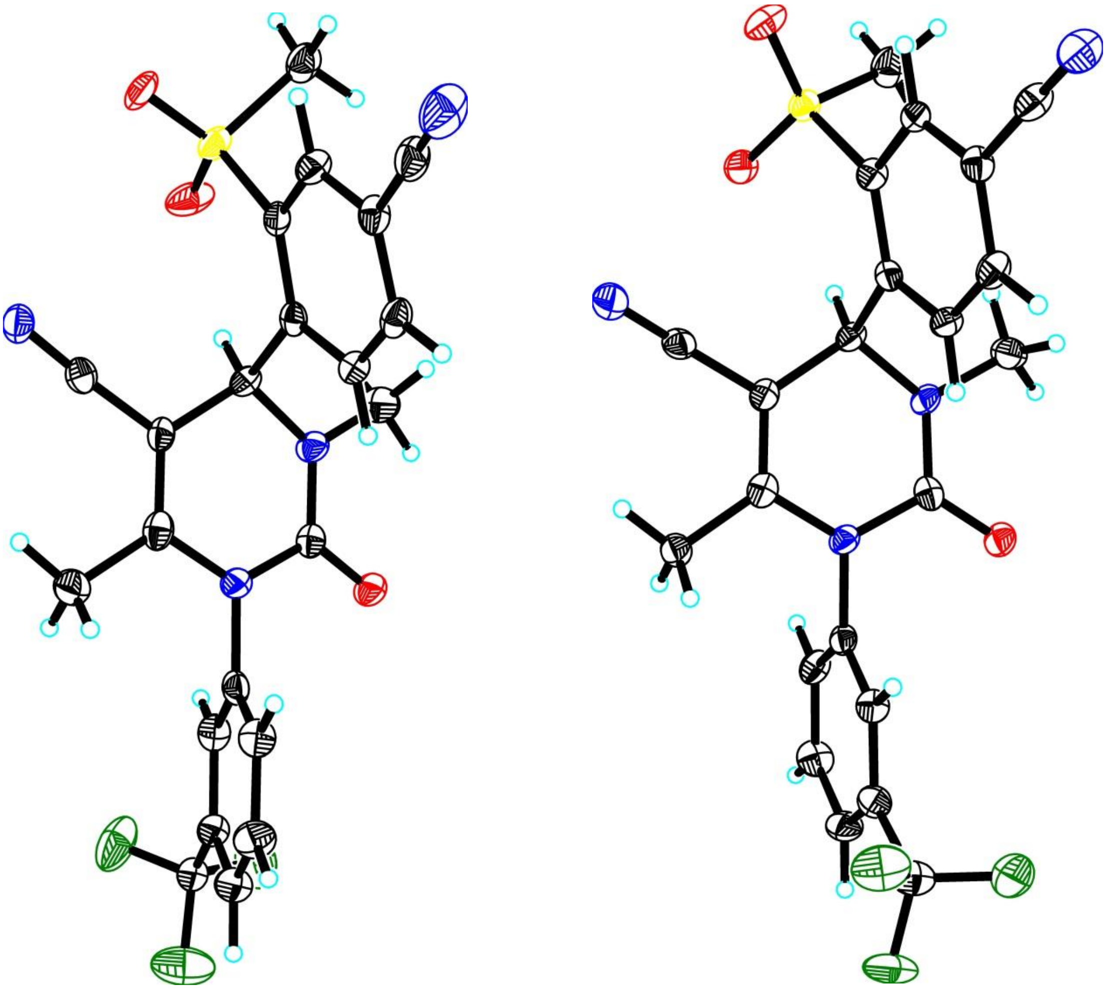

Figure S4. ORTEP plot of **29** (BAY 85-8501) (both molecules shown separately) showing 50% thermal ellipsoids.

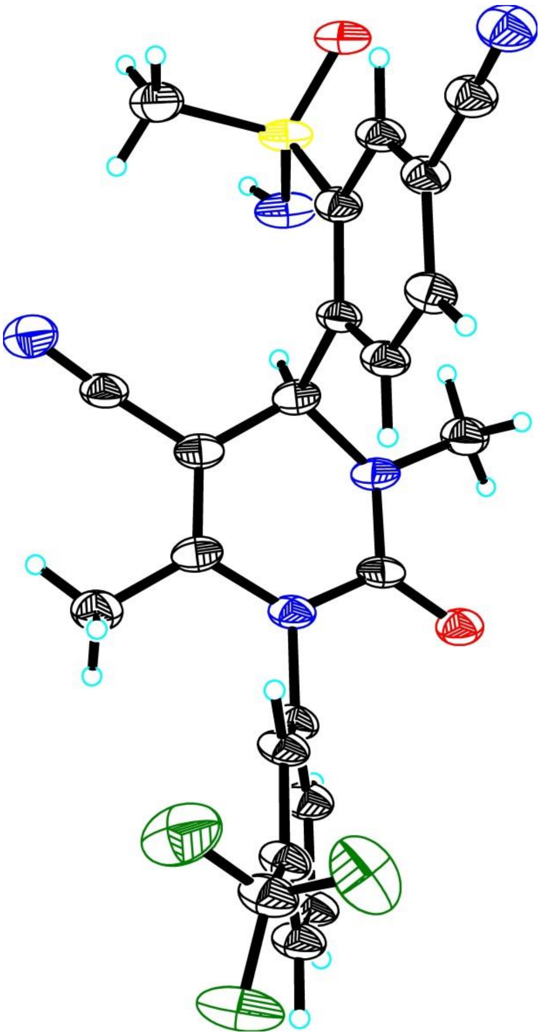

Figure S5. ORTEP plot of **30** showing 50% thermal ellipsoids.

EXPERIMENTAL PROCEDURES

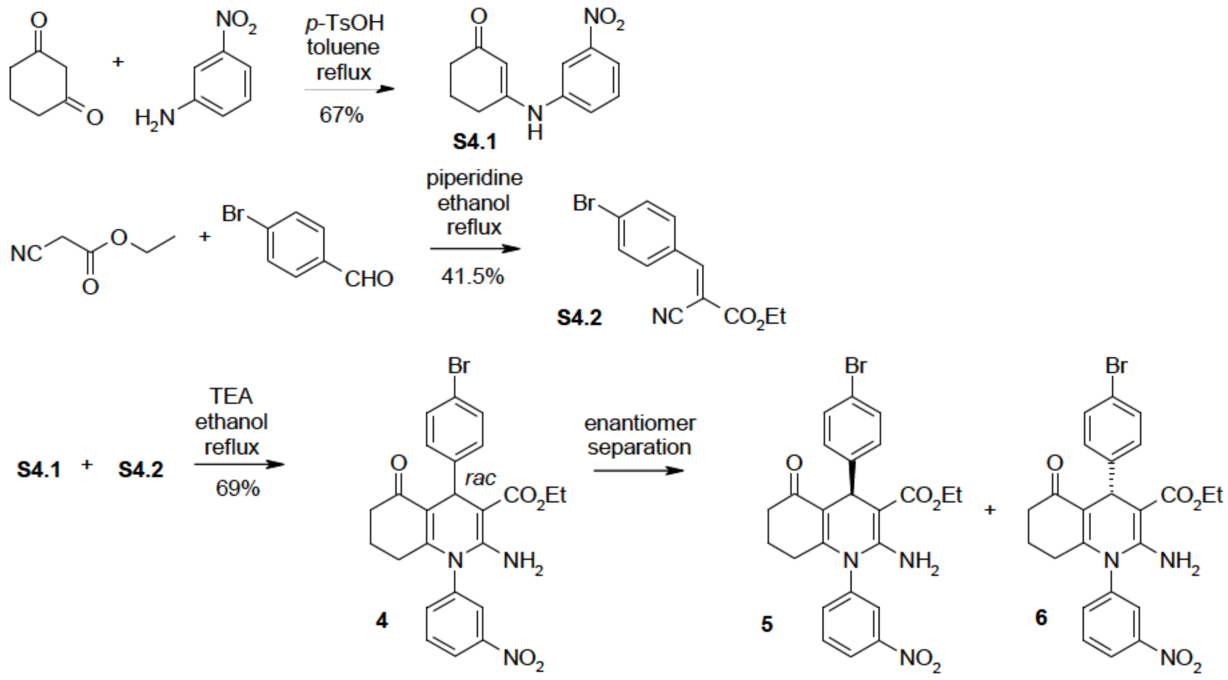

Scheme 1. Synthesis of quinolines **4–6**.

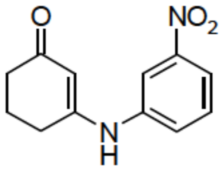

3-[(3-Nitrophenyl)amino]cyclohex-2-en-1-one (S4.1): Cyclohexane-1,3-dione (10 g, 89.2 mmol) and 3-nitroaniline (12.32 g, 89.2 mmol) were dissolved in toluene. 4-Toluenesulfonic acid (1.54 g, 8.92 mmol) was added. The reaction mixture was heated at reflux overnight with a Dean–Stark trap to remove water. After cooling to room temperature, the solvent was removed in vacuo. The residue was purified by column chromatography on silica gel (cyclohexane/ethyl acetate). Yield 13.9 g (67%); ^{1}H NMR (400 MHz, [D$_6$]DMSO): δ = 1.46 (quin, 2H), 1.75 (t, 2H), 2.08 (t, 2H), 2.86 (s, 1H), 5.01 (s, 1H), 7.16–7.18 (m, 1H), 7.46 (dt, 1H), 7.49–7.52 (m, 1H), 8.69 (s, 1H) ppm.

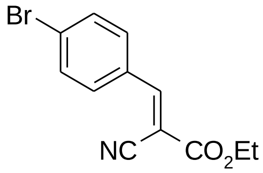

Ethyl (2*E*/*Z*)–3–(4–bromophenyl)–2–cyanoacrylate (S4.2): To a solution of ethyl cyanoacetate (5 g, 44.2 mmol) in ethanol (30 mL) were added 4-bromobenzaldehyde (8.18 g, 44.2 mmol) and piperidine (4.52 g, 5.25 mL, 53.0 mmol). The reaction mixture was heated at reflux for 6 h. The mixture was cooled to 0°C. The formed precipitate was collected by filtration. Yield 4.09 g (33%). The filtrate was concentrated and the residue was purified by flash chromatography on silica gel (cyclohexane/ethyl acetate 50:1 → 20:1). Yield 1.05 g (8.5%); ^{1}H NMR (200 MHz, [D$_6$]DMSO): δ = 1.31 (t, 3H), 4.32 (q, 2H), 7.77–7.89 (m, 2H), 7.95–8.05 (m, 2H), 8.41 (s, 1H) ppm; MS (CI+) *m*/*z*: 297 [*M*+NH$_4$ (^{79}Br)]$^+$, 299 [*M*+NH$_4$ (^{81}Br)]$^+$.

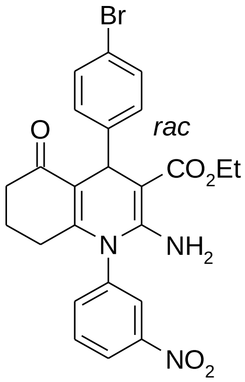

Ethyl (*rac*)-2-amino-4-(4-bromophenyl)-1-(3-nitrophenyl)-5-oxo-1,4,5,6,7,8-hexahydroquinoline-3-carboxylate (4): To a solution of **S4.1** (1 g, 4.3 mmol) in ethanol (10 mL) were added triethylamine (TEA; 1.2 mL, 8.6 mmol) and **S4.2** (1.2 g, 4.3 mmol), under argon. The mixture was heated at reflux for 15 h and then cooled to 0°C. The suspension was filtered and the solid was washed with small amounts of ethanol and dried in vacuo. Yield 1.52 g (69%); ^{1}H NMR (300 MHz, [D$_6$]DMSO): δ = 1.21 (t, 3H), 1.68 (br. s, 1H), 1.75–1.9 (m, 2H), 2.18 (s, 2H), 2.29 (m, 1H), 4.0 (m, 2H), 5.95 (s, 1H), 6.93 (br. s, 2H), 7.29 (m, 2H), 7.42 (m, 2H), 7.89 (m, 2H), 8.36 (s, 1H), 8.42 (m, 1H) ppm; MS (ESI-) *m*/*z*: 512.1 [*M*+H]$^+$.

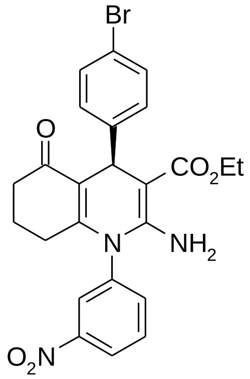

Ethyl (4*R*)-2-amino-4-(4-bromophenyl)-1-(3-nitrophenyl)-5-oxo-1,4,5,6,7,8-hexahydroquinoline-3-carboxylate (5): The enantiomers of **4** (500 mg) were separated by preparative HPLC on a chiral phase (column: KBD 6371, 250 mm × 20 mm, 10 µm; eluent: hexane/ethyl acetate 47:53; UV detection: 254 nm). A crude product was obtained that was further purified by flash chromatography on silica gel (cyclohexane/ethyl acetate 3:1). Yield 16.6 mg (3%); ^{1}H NMR (300 MHz, [D$_6$]DMSO): δ = 1.21 (t, 3H), 1.68 (br. s, 1H), 1.75–1.9 (m, 2H), 2.18 (s, 2H), 2.29 (m, 1H), 4.0 (m, 2H), 5.95 (s, 1H), 6.93 (br. s, 2H), 7.29 (m, 2H), 7.42 (m, 2H), 7.89 (m, 2H), 8.36 (s, 1H), 8.42 (m, 1H) ppm; MS (ESI+) *m*/*z*: 512 [*M*+H]$^+$.

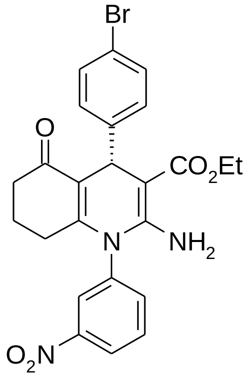

Ethyl (4*S*)-2-amino-4-(4-bromophenyl)-1-(3-nitrophenyl)-5-oxo-1,4,5,6,7,8-hexahydroquinoline-3-carboxylate (6): 6 was obtained as a second enantiomer (vide supra). Yield 50 mg (10%); ^{1}H NMR (300 MHz, [D$_6$]DMSO): δ = 1.21 (t, 3H), 1.68 (br. s, 1H), 1.75–1.9 (m, 2H), 2.18 (s, 2H), 2.29 (m, 1H), 4.0 (m, 2H), 5.95 (s, 1H), 6.93 (br. s, 2H), 7.29 (m, 2H), 7.42 (m, 2H), 7.89 (m, 2H), 8.36 (s, 1H), 8.42 (m, 1H) ppm; MS (ESI+) *m*/*z*: 512 [*M*+H]$^+$.

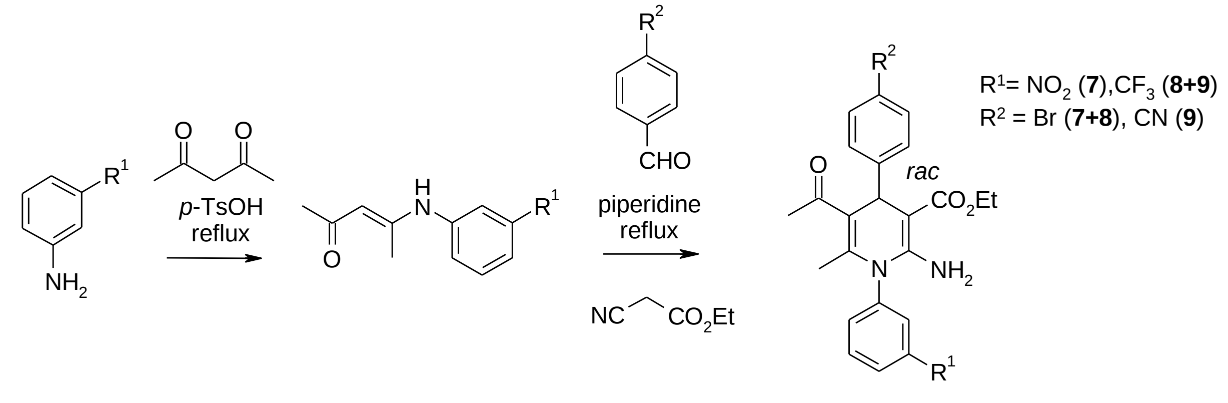

Scheme 2. Synthesis of dihydropyridines **7–9**.

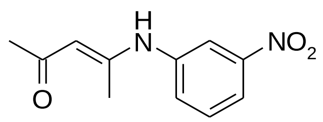

4-[(3-Nitrophenyl)amino]pent-3-en-2-one (S7.1): Acetylacetone (36.24 g, 362 mmol), 3-nitroaniline (10.00 g, 72 mmol) and 4-toluenesulfonic acid (1.25 g, 7.2 mmol) were dissolved in toluene (100 mL). The reaction mixture was heated at reflux overnight with a Dean–Stark trap to remove water. After cooling to room temperature, the solvent was removed in vacuo. The residue was purified by column chromatography on silica gel (dichloromethane). Yield 12.0 g (75%); ^{1}H NMR (400 MHz, [D$_6$]DMSO): δ = 2.0 (s, 3H), 2.1 (s, 3H), 5.4 (s, 1H), 7.7 (m, 2H), 8.0 (m, 2H), 12.5 (s, 1H) ppm; MS (CI+) *m*/*z*: 221 [*M*+H]$^+$, 238 [*M*+NH$_4$]$^+$.

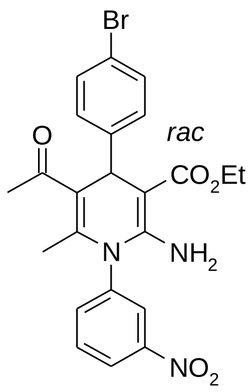

Ethyl (*rac*)-5-acetyl-2-amino-4-(4-bromophenyl)-6-methyl-1-(3-nitrophenyl)-1,4-dihydropyridine-3-carboxylate (7): To **S7.1** (4.8 g, 21.8 mmol) in ethanol (30 mL) were added 4-bromobenzaldehyde (4.0 g, 21.8 mmol), ethyl cyanoacetate (2.47 g, 21.8 mmol) and piperidine (3.71 g, 43.6 mmol). The reaction mixture was heated at

reflux overnight. After cooling to room temperature, the solvent was removed in vacuo. The residue was purified by column chromatography on silica gel (dichloromethane). Yield 1.8 g (17%); ^{1}H NMR (300 MHz, [D$_6$]DMSO): δ = 1.2 (t, 3H), 1.9 (s, 3H), 2.2 (s, 3H), 4.0 (m, 2H), 4.9 (s, 1H), 6.8 (br. s, 2H), 7.3 (m, 2H), 7.5 (m, 2H), 7.8 (m, 2H), 8.2 (m, 1H), 8.4 (m, 1H) ppm; MS (ESI+) *m*/*z*: 500 [*M*+H (^{79}Br)]$^+$, 502 [*M*+H (^{81}Br)]$^+$.

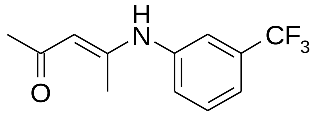

4-{[3-(Trifluoromethyl)phenyl]amino}pent-3-en-2-one (S8.1): Acetylacetone (15.53 g, 155 mmol), 3-(trifluoromethyl)aniline (5.00 g, 31 mmol) and 4-toluenesulfonic acid (0.53 g, 3.1 mmol) were dissolved in toluene (50 mL). The reaction mixture was heated at reflux overnight with a Dean–Stark trap to remove water. After cooling to room temperature, the solvent was removed in vacuo. The residue was purified by column chromatography on silica gel (cyclohexane/ethyl acetate mixtures). Yield 5.46 g (72%); ^{1}H NMR (200 MHz, [D$_6$]DMSO): δ = 2.0 (s, 3H), 2.1 (s, 3H), 5.3 (s, 1H), 7.5 (m, 4H), 12.5 (s, 1H) ppm; MS (CI+) *m*/*z*: 244 [*M*+H]$^+$, 261 [*M*+NH_4]$^+$.

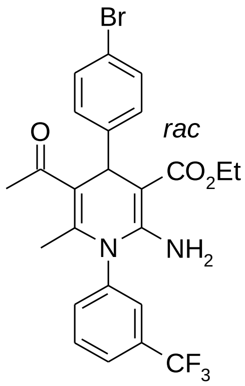

Ethyl (*rac*)-5-acetyl-2-amino-4-(4-bromophenyl)-6-methyl-1-[3-(trifluoromethyl)-phenyl]-1,4-dihydropyridine-3-carboxylate (8): To a solution of **S8.1** (150 mg, 0.62 mmol) in ethanol (2 mL) were added 4-bromobenzaldehyde (114 mg, 0.62 mmol), ethyl cyanoacetate (70 mg, 0.62 mmol) and piperidine (105 mg, 1.23 mmol). The reaction mixture was heated at reflux overnight. After cooling to room temperature, the solvent was removed in vacuo. The residue was purified by column chromatography on silica gel (dichloromethane). Yield 43 mg (13%); ^{1}H NMR

(300 MHz, [D6]DMSO): δ = 1.2 (t, 3H), 1.8 (s, 3H), 2.2 (s, 3H), 4.0 (m, 2H), 4.9 (s, 1H), 6.7 (br. s, 2H), 7.3 (m, 2H), 7.5 (m, 2H), 7.7 (m, 1H), 7.8 (m, 1H), 7.8 (m, 1H), 7.9 (m, 1H) ppm; MS (ESI+) *m*/*z*: 523 [*M*+H (^{79}Br)]$^+$, 525 [*M*+H (^{81}Br)]$^+$.

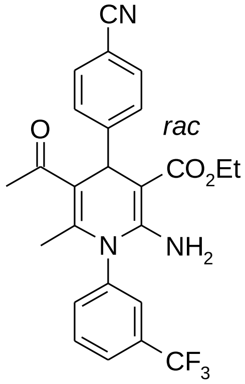

Ethyl (*rac*)-5-acetyl-2-amino-4-(4-cyanophenyl)-6-methyl-1-[3-(trifluoromethyl)-phenyl]-1,4-dihydropyridine-3-carboxylate (9): To a solution of **S8.1** (100 mg, 0.41 mmol) in ethanol (2 mL) were added 4-cyanobenzaldehyde (54 mg, 0.41 mmol), ethyl cyanoacetate (47 mg, 0.41 mmol) and piperidine (70 mg, 0.82 mmol). The reaction mixture was heated at reflux overnight. After cooling to room temperature, the solvent was removed in vacuo. The residue was purified by column chromatography on silica gel (dichloromethane). Yield 26 mg (14%); ^{1}H NMR (400 MHz, [D6]DMSO): δ = 1.2 (t, 3H), 1.8 (s, 3H), 2.2 (s, 3H), 4.0 (m, 2H), 5.0 (s, 1H), 6.7 (br. s, 2H), 7.5 (m, 2H), 7.7 (m, 1H), 7.8 (m, 4H), 7.9 (m, 1H) ppm; MS (ESI+) *m*/*z*: 470 [*M*+H]$^+$.

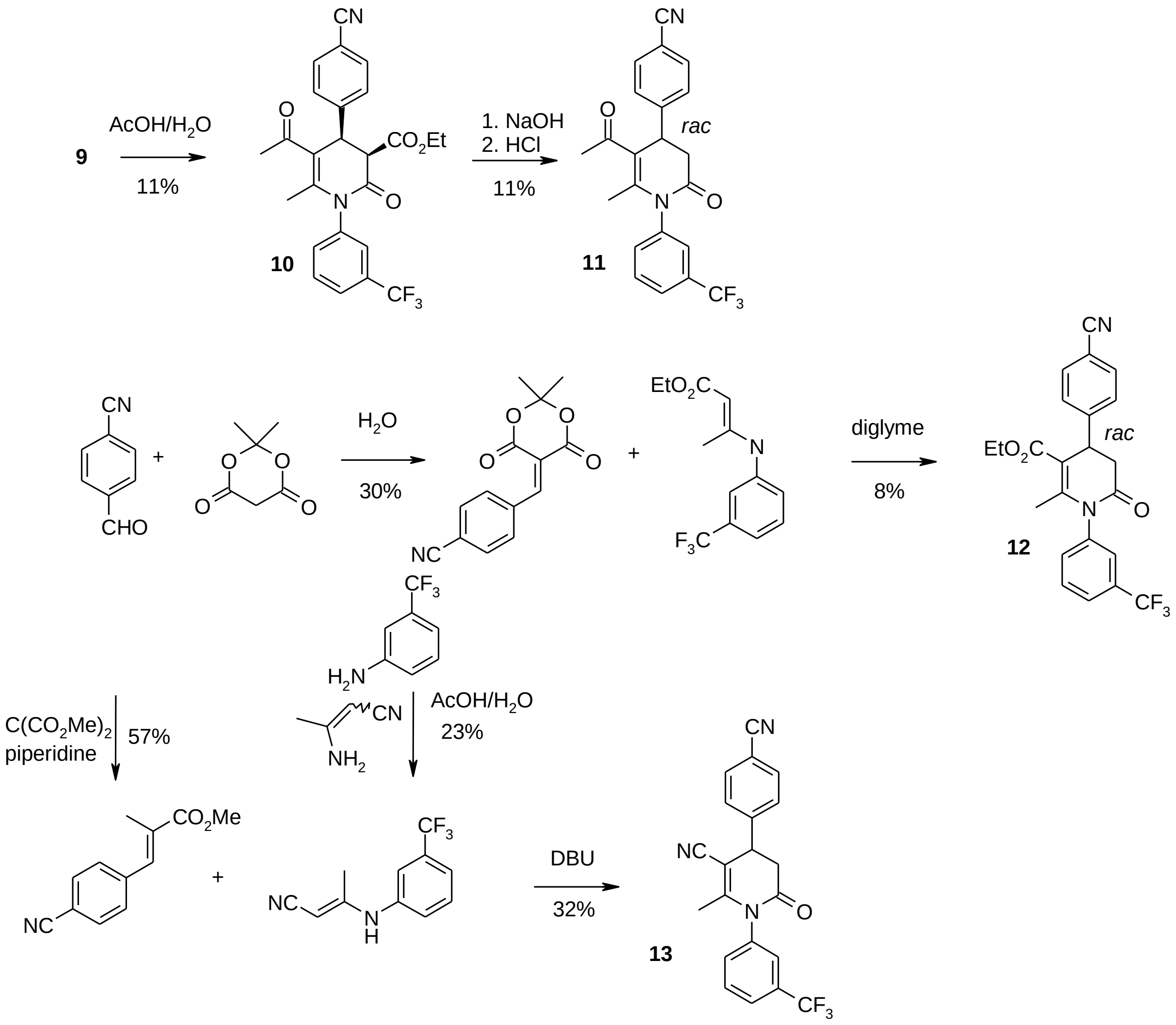

Scheme 3. Synthesis of compounds **10-14**.

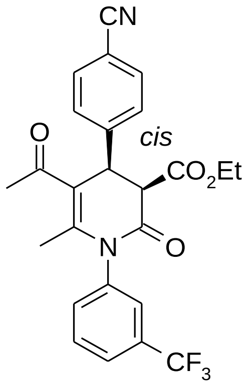

Ethyl 3,4-*cis*-5-acetyl-4-(4-cyanophenyl)-6-methyl-2-oxo-1-[3-(trifluoromethyl)-phenyl]-1,2,3,4-tetrahydropyridine-3-carboxylate (10): 9 (100 mg, 0.21 mmol) was dissolved in acetic acid (2 mL) and H_2O (0.2 mL). The mixture was stirred at reflux for 18 h. After cooling to room temperature, the solvent was removed in vacuo and the residue was purified by column chromatography on silica gel (cyclohexane/ethyl

acetate mixtures). Yield 11 mg (11%); ^{1}H NMR (200 MHz, [D$_6$]DMSO): δ = 1.2 (t, 3H), 2.0 (s, 3H), 2.2 (s, 3H), 4.1 (d, 1H), 4.2 (q, 2H), 4.8 (d, 1H), 7.44–7.53 (m, 2H), 7.63–7.90 (m, 6H) ppm; MS (ESI+) *m*/*z*: 471 [*M*+H]$^+$.

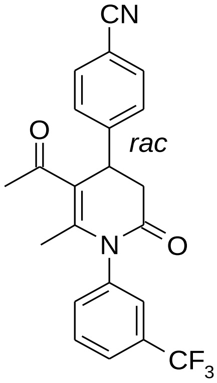

(*rac*)-4-{5-Acetyl-6-methyl-2-oxo-1-[3-(trifluoromethyl)phenyl]-1,2,3,4-tetra-hydropyridin-4-yl}benzonitrile (11): 10 (1.50 g, 3.2 mmol) was suspended in dioxane/H_2O (2:1 v/v, 22.5 mL). Sodium hydroxide (45% aqueous solution; 0.56 mL, 6.4 mmol) was added. The reaction mixture was stirred at room temperature for 4 h. The mixture was acidified with 1 N hydrochloric acid to pH 4 and extracted with dichloromethane (3 ×). The combined organic phases were dried over sodium sulfate and the solvent was removed in vacuo. The residue was purified by column chromatography on silica gel (cyclohexane/ethyl acetate 3:1 → 2:1, then dichloromethane/methanol/formic acid 12:1:0.1). The resulting crude product was further purified by preparative HPLC (RP column; eluent: acetonitrile/H_2O + 0.1% TFA mixtures). Yield 146 mg (11%); HPLC (method 2): t_R = 4.65 min; ^{1}H NMR (200 MHz, $CDCl_3$): δ = 2.11 (s, 3H), 2.20 (s, 3H), 2.97 (dd, 1H), 3.21 (dd, 1H), 4.31 (m, 1H), 7.15–7.77 (m, 8H) ppm; MS (CI+) *m*/*z*: 399 [*M*+H]$^+$.

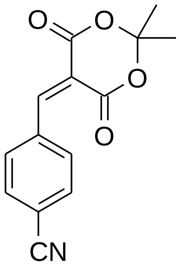

4-[(2,2-Dimethyl-4,6-dioxo-1,3-dioxan-5-ylidene)methyl]benzonitrile (S12.1): This reaction was carried out by analogy to a described procedure.[7] 4-Cyanobenz-aldehyde (5.30 g, 40.5 mmol) and 2,2-dimethyl-1,3-dioxane-4,6-dione (7.93 g,

55.0 mmol) were stirred in H_2O (100 mL) at 75°C. The precipitate was collected by filtration and recrystallized from ethanol. Yield 3.04 g (30%); mp: 180°C (with decomposition); ^{1}H NMR (200 MHz, [D$_6$]DMSO): δ = 1.78 (s, 6H), 7.95 (d, 2H), 8.03 (d, 2H), 8.45 (s, 1H) ppm; MS (CI+) *m*/*z*: 275 [*M*+NH_4]$^+$.

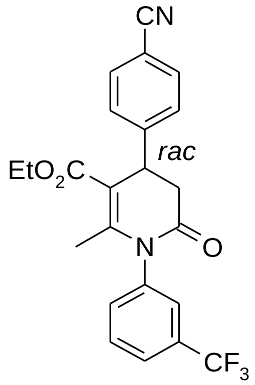

Ethyl (*rac*)-4-(4-cyanophenyl)-2-methyl-6-oxo-1-[3-(trifluoromethyl)phenyl]-1,4,5,6-tetrahydropyridine-3-carboxylate (12): Compound **S12.1** (200 mg, 0.77 mmol) and ethyl (2*E*)-3-{[3-(trifluoromethyl)phenyl]amino}but-2-enoate (212.4 mg, 0.77 mmol) were dissolved in 1-methoxy-2-(2-methoxyethoxy)ethane (3 mL). The solution was heated at reflux overnight. The reaction mixture was cooled to room temperature, diluted with H_2O (5 mL) and extracted with toluene (2 × 5 mL). The extracts were dried over sodium sulfate then filtered, and the solvent was removed in vacuo. The product was purified via preparative HPLC (RP column; eluent: acetonitrile/H_2O + 0.01% TFA-mixtures). Yield 28 mg (8%); LC-MS (method 1): t_R = 4.05 min; ^{1}H NMR (200 MHz, [D$_6$]DMSO): δ = 1.09 (t, 3H), 2.10 (s, 3H), 2.7 (dd, 1H), 3.25 (dd, 1H), 4.05 (q, 2H), 4.4 (m, 1H), 7.5–7.9 (m, 8H) ppm; MS (ESI+) *m*/*z*: 429 [*M*+H]$^+$.

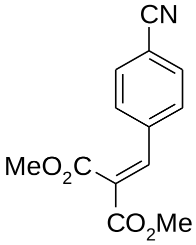

Dimethyl 2-(4-cyanobenzylidene)malonate (S13.1): Dimethyl malonate (5.04 g, 38.13 mmol), 4-cyanobenzaldehyde (5.00 g, 38.13 mmol) and piperidine (0.097 g, 1.1 mmol) were dissolved in methanol (150 mL). The reaction mixture was stirred at room temperature for 48 h. The solvent was removed in vacuo to afford a viscous oil

which was crystallized from methanol. Yield 5.3 g (57%); HPLC (method 2): t_R = 3.94 min; mp: 98–99°C; ^{1}H NMR (300 MHz, [D$_6$]DMSO): δ = 3.80 (s, 3H), 3.81 (s, 3H), 7.6–8.0 (m, 5H) ppm; MS (CI+) *m*/*z*: 246 [*M*+H]$^+$, 263 [*M*+NH$_4$]$^+$.

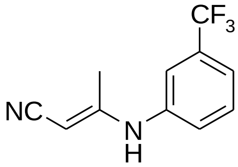

3-{[3-(Trifluoromethyl)phenyl]amino}but-2-enenitrile (S13.2): 3-Aminocrotononitrile (1.0 g, 12.2 mmol), 3-(trifluoromethyl)aniline (2.0 g, 12.4 mmol) and acetic acid (1.23 g, 20.5 mmol) were dissolved in H_2O (8 mL). The reaction mixture was stirred at room temperature for 30 min. The mixture was extracted with toluene (3 ×) and the organic phase was dried over sodium sulfate. The solvent was removed in vacuo and the residue was purified by column chromatography on silica gel (cyclohexane/ethyl acetate mixtures). Yield 0.64 g (23%); ^{1}H NMR (300 MHz, [D$_6$]DMSO): δ = 2.2 (s, 3H), 4.6 (s, 1H), 7.4–7.6 (m, 4H), 9.0 (s, 1H) ppm; MS (ESI+) *m*/*z*: 227 [*M*+H]$^+$.

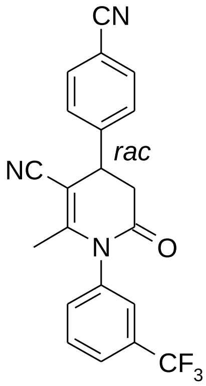

(*rac*)-4-(4-Cyanophenyl)-2-methyl-6-oxo-1-[3-(trifluoromethyl)phenyl]-1,4,5,6-tetrahydropyridine-3-carbonitrile (13): To a solution of **S13.1** (50 mg, 0.20 mmol) and **S13.2** (35 mg, 0.16 mmol) in *tert*-butyl alcohol (1.5 mL) was added 1,8-diazabicyclo[5.4.0]undec-7-ene (DBU; 2.36 mg, 0.02 mmol). The reaction mixture was heated at reflux overnight. Additional amounts of compound **S13.1** (10 mg, 0.04 mmol) and DBU (2.36 mg, 0.02 mmol) were added. The reaction mixture was heated again at reflux overnight. The solvent was removed in vacuo. The residue was purified by column chromatography on silica gel (cyclohexane/ethyl acetate 3:1). Yield 20.8 mg (32%); HPLC (method 2): t_R = 4.63 min; ^{1}H NMR (200 MHz, CDCl$_3$):

δ = 2.02 (d, 3H), 3.02 (dd, 1H), 3.21 (dd, 1H), 4.07 (dd, 1H), 7.35–7.80 (m, 8H) ppm; MS (CI+) *m*/*z*: 399 [*M*+NH_4]$^+$.

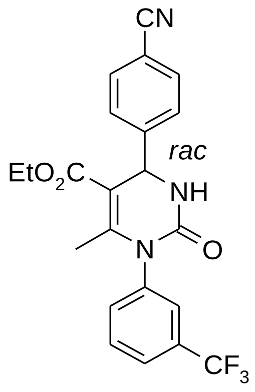

Ethyl (*rac*)-4-(4-cyanophenyl)-6-methyl-2-oxo-1-[3-(trifluoromethyl)phenyl]-1,2,3,4-tetrahydropyrimidine-5-carboxylate (S14.1): 1-[3-(Trifluoromethyl)phenyl]-urea (7.0 g, 34.29 mmol), 4-cyanobenzaldehyde (8.99 g, 68.58 mmol), ethyl 3-oxobutanoate (8.92 g, 68.58 mmol) and polyphosphoric acid ethyl ester (20 g) were suspended in tetrahydrofuran (THF; 250 mL). The mixture was heated at reflux for 18 h. After cooling to room temperature, the solvent was removed in vacuo and the residue was purified by column chromatography on silica gel (toluene/ethyl acetate 10:1 → 5:1 → 2:1). Yield 13.4 g (91%); ^{1}H NMR (200 MHz, [D$_6$]DMSO): δ = 1.1 (t, 3H), 2.0 (s, 3H), 4.0 (q, 2H), 5.4 (d, 1H), 7.6 (m, 3H), 7.7 (m, 3H), 7.9 (m, 2H), 8.4 (d, 1H) ppm; MS (ESI+) *m*/*z*: 430 [*M*+H]$^+$.

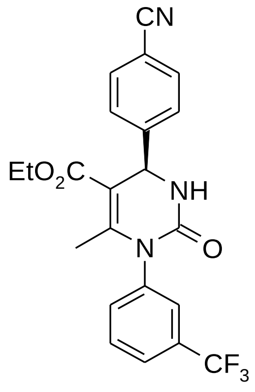

Ethyl (4*R*)-4-(4-cyanophenyl)-6-methyl-2-oxo-1-[3-(trifluoromethyl)phenyl]-1,2,3,4-tetrahydropyrimidine-5-carboxylate (14): S14.1 (60 g) was dissolved in ethyl acetate (360 mL) and fractionated into the enantiomers by preparative HPLC on a chiral phase [column: KBD 7644 chiral silica gel phase based on the selector poly(*N*-methacryloyl-D-leucine-3-pentylamide), 500 mm × 65 mm, 10 µm; eluent:

ethyl acetate (0–16.33 min) → methanol (16.34–24.12 min) → ethyl acetate (24.13–35.0 min); flow: 100 mL/min; temperature: 24°C; detection: 340 nm]. Yield 29.9 g (99%, 99.1% ee). HPLC [column: chiral silica gel phase based on the selector poly(*N*-methacryloyl-D-leucine-*tert*-butylamide), 250 mm × 4.6 mm; eluent: ethyl acetate; flow: 2 mL/min; temperature: 24°C; detection: 260 nm]: t_R = 1.55 min. $[\alpha]_D^{20}$ = +3.3 (*c* = 0.535 in dichloromethane); ^{1}H NMR (300 MHz, $[D_6]$DMSO): δ = 1.1 (t, 3H), 2.0 (s, 3H), 4.0 (q, 2H), 5.4 (d, 1H), 7.6 (m, 3H), 7.7 (m, 2H), 7.8 (m, 1H), 7.9 (m, 2H), 8.4 (d, 1H) ppm; MS (ESI+) *m*/*z*: 430 $[M+H]^+$. Also, the isomeric ethyl (4*S*)-4-(4-cyanophenyl)-6-methyl-2-oxo-1-[3-(trifluoromethyl)phenyl]-1,2,3,4-tetrahydro-pyrimidine-5-carboxylate was obtained.

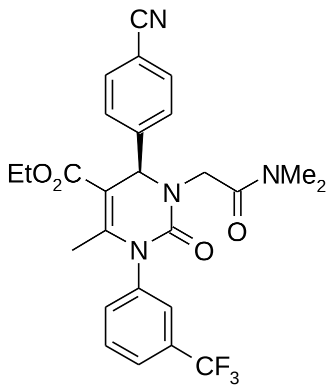

Ethyl (4*R*)-4-(4-cyanophenyl)-3-[2-(dimethylamino)-2-oxoethyl]-6-methyl-2-oxo-1-[3-(trifluoromethyl)phenyl]-1,2,3,4-tetrahydropyrimidine-5-carboxylate (15): To **16** (975 mg, 2.00 mmol) in *N*,*N*-dimethylformamide (DMF; 4 mL) were added a 2 M solution of dimethylamine in THF (2.2 mL, 4.401 mmol), 4-(*N*,*N*-dimethylamino)pyridine (449 mg, 4.00 mmol), 1-hydroxy-1*H*-benzotriazole hydrate (595 mg, 4.401 mmol) and 1-(3-dimethylaminopropyl)-3-ethylcarbodiimide hydrochloride (844 mg, 4.401 mmol). The mixture was stirred at room temperature overnight. After the mixture was concentrated in a rotary evaporator, the crude product was purified by preparative HPLC (column: GromSil C-18, 10 µm; eluent: acetonitrile + 0.1% TFA /H_2O + 0.1% TFA 10:90 → 95:5 → 10:90; flow: 50 mL/min; UV detection: 210 nm). Yield 795 mg (77%); LC-MS (method 5): t_R = 3.67 min; ^{1}H NMR (400 MHz, $[D_6]$DMSO): δ = 1.1 (t, 3H); 2.0 (s, 3H); 2.82(s, 3H); 2.87(s,3H); 3.63 (d, 2H); 4.0 (m, 2H); 5.5 (s, 1H); 7.61 (m, 1H); 7.67 (m, 2H), 7.72 (m, 2H); 7.82 (m, 1H); 7.88 (m, 2H) ppm; MS (ESI+) *m*/*z*: 515.4 $[M+H]^+$.

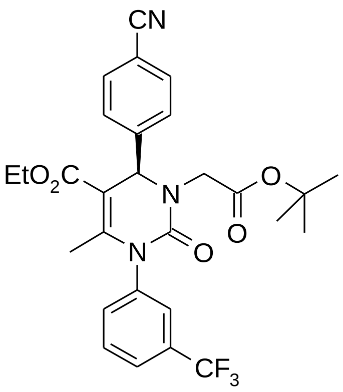

Ethyl (4*R*)-3-(2-*tert*-butoxy-2-oxoethyl)-4-(4-cyanophenyl)-6-methyl-2-oxo-1-[3-(trifluoromethyl)phenyl]-1,2,3,4-tetrahydropyrimidine-5-carboxylate (S16.1): Under argon, **14** (10.74 g, 25 mmol) and potassium carbonate (5.53 g, 40 mmol) were suspended in DMF (100 mL). *tert*-Butyl bromoacetate (7.8 g, 40 mmol) was added slowly. The reaction mixture was stirred at room temperature for 20 h, and then at 60°C for 3 h. The solvent was evaporated. The residue was dissolved in ethyl acetate (200 mL), and washed with H_2O (2 × 50 mL) and brine, and the organic layer was dried over sodium sulfate. The solvent was evaporated and the residue was submitted to flash chromatography on silica gel (cyclohexane 100% → cyclohexane/ethyl acetate 6:4). Yield 12.5 g (91%); LC-MS (method 6): t_R = 2.94 min; ^{1}H NMR (400 MHz, [D$_6$]DMSO): δ = 1.05 (t, 3H), 1.25 (s, 9H), 2.05 (s, 3H), 3.85 (d, 1H), 4.0 (m, 3H), 5.55 (s, 1H), 7.60–7.90 (m, 8H) ppm; MS (ESI+) *m*/*z*: 488.1 [*M*+H–C_4H_8]$^+$; MS (ESI–) *m*/*z*: 542.2 [*M*–H]$^-$.

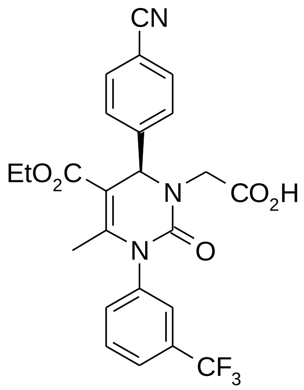

{(6*R*)-6-(4-Cyanophenyl)-5-(ethoxycarbonyl)-4-methyl-2-oxo-3-[3-(trifluoromethyl)phenyl]-3,6-dihydropyrimidin-1(2*H*)-yl}acetic acid (16): Under argon, to a solution of **S16.1** (165 mg, 0.30 mmol) in dichloromethane (1.5 mL) was added trifluoroacetic acid (TFA; 1.5 mL). The mixture was stirred at room temperature for 4 h. The solvent was evaporated and the residue was purified by preparative HPLC

(instrument: Gilson Abimed HPLC; column: Kromasil-100A C-18, 250 mm × 20 mm, 5 µm; eluent: H_2O + 0.1% TFA/acetonitrile + 0.1% TFA; 90:10 → 10:90; flow: 25 mL/min; UV detection: 210 nm). Yield 132 mg (89%); analytical HPLC (method 7): t_R = 3.0 min; LC-MS (method 8): t_R = 2.3 min; ^{1}H NMR (400 MHz, [D$_6$]DMSO): δ = 1.10 (t, 3H), 2.05 (s, 3H), 3.75 (d, 1H), 4.05 (q, 2H), 4.10 (d, 1H), 5.60 (s, 1H), 7.60–7.75 (m, 5H), 7.80 (d, 1H), 7.90 (d, 2H), 12.70 (s, 1H) ppm; MS (ESI+) *m/z*: 488.1 [*M*+H]$^+$.

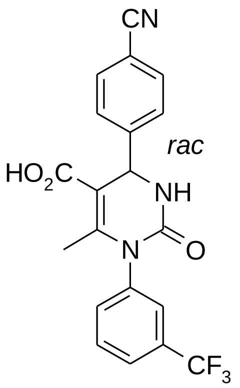

(*rac*)-4-(4-Cyanophenyl)-6-methyl-2-oxo-1-[3-(trifluoromethyl)phenyl]-1,2,3,4-tetrahydropyrimidine-5-carboxylic acid (S17.1): S14.1 (3 g, 7 mmol) was stirred in a mixture of H_2O (50 mL) and 5% KOH in ethanol (100 mL) at room temperature for 18 h. The reaction mixture was acidified with 1N hydrochloric acid to pH2. The formed precipitate was filtered, washed with water and dried. Yield 1.27 g (45%); ^{1}H NMR (300 MHz, [D$_6$]DMSO): δ = 2.0 (s, 3H), 5.4 (d, 1H), 7.6 (m, 1H), 7.6 (m, 2H), 7.7 (m, 1H), 7.8 (m, 1H), 7.9 (m, 3H), 8.3 (d, 1H), 12.5 (s, 1H) ppm; MS (ESI+) *m/z*: 402 [*M*+H]$^+$.

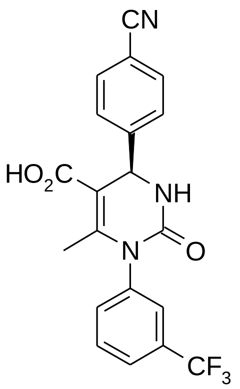

(4*R*)-4-(4-Cyanophenyl)-6-methyl-2-oxo-1-[3-(trifluoromethyl)phenyl]-1,2,3,4-tetrahydropyrimidine-5-carboxylic acid (S17.2): The enantiomers of **S17.1** were

separated by preparative HPLC on a chiral phase [column: KBD 8361 (chiral silica gel phase based on the monomer selector *N*-methacryloyl-L-leucine-1-menthylamide[8]), 250 mm × 20 mm; eluent: ethyl acetate → methanol → ethyl acetate; flow: 25 mL/min; temperature: 23°C; UV detection: 254 nm]. $[\alpha]_D^{20}$ = +2.5 (*c* = 0.505 in methanol); ^{1}H NMR (300 MHz, [D$_6$]DMSO): δ = 2.0 (s, 3H), 5.4 (d, 1H), 7.6 (m, 1H), 7.6 (m, 2H), 7.7 (m, 1H), 7.8 (m, 1H), 7.9 (m, 3H), 8.3 (d, 1H), 12.5 (s, 1H) ppm; MS (ESI+) *m*/*z*: 402 [*M*+H]$^+$.

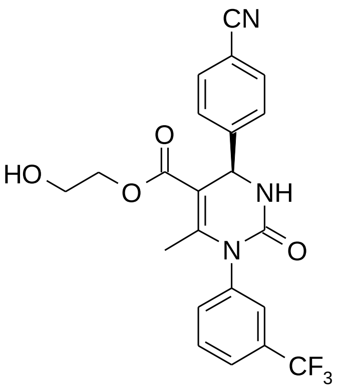

2-Hydroxyethyl (4*R*)-4-(4-cyanophenyl)-6-methyl-2-oxo-1-[3-(trifluoromethyl)-phenyl]-1,2,3,4-tetrahydropyrimidine-5-carboxylate (17): Under argon, **S17.2** (1560 mg, 3.89 mmol) was added to DMF (19.6 mL). After the addition of TEA (1.1 mL, 7.86 mmol) and 2-bromoethanol (1.11 mL, 15.7 mmol), the reaction mixture was stirred at about 70°C for 8 h. After cooling, the reaction mixture was concentrated in vacuo. The residue was taken up in ethyl acetate and washed with H_2O. The organic phase was dried with magnesium sulfate and concentrated in vacuo. The residue was taken up in methanol (8 mL) and purified by preparative HPLC (column: Nucleosil 100-5 C-18 Nautilus, 20 mm × 50 mm, 5 μm; eluent A: acetonitrile; eluent B: H_2O + 0.3% formic acid; gradient: 0 min 10% A, 2 min 10% A, 6 min 90% A, 7 min 90% A, 7.1 min 10% A, 8 min 10% A; wavelength: 220 nm; injection volume: ca. 500 μL; number of injections: 18). The product-containing fractions were combined and lyophilized. Yield 1290 mg (74.5%); $[\alpha]_D^{20}$ = +14.3 (*c* = 0.455 in methanol); ^{1}H NMR (300 MHz, [D$_6$]DMSO): δ = 2.05 (d, 3H), 3.5 (q, 2H), 3.95–4.15 (m, 2H), 4.75 (t, 1H), 5.45 (d, 1H), 7.55–7.75 (m, 5H), 7.75 (d, 1H), 7.85 (d, 2H), 8.35 (d, 1H) ppm; MS (ESI+) *m*/*z*: 446 [*M*+H]$^+$.

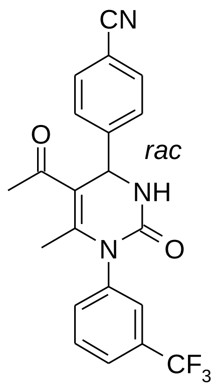

(*rac*)-4-{5-Acetyl-6-methyl-2-oxo-1-[3-(trifluoromethyl)phenyl]-1,2,3,4-tetra-hydropyrimidin-4-yl}benzonitrile (S18.1): 1-[3-(Trifluoromethyl)phenyl]urea (265 mg, 1.3 mmol), 4-cyanobenzaldehyde (131 mg, 1.0 mmol) and pentane-2,4-dione (100 mg, 1.0 mmol) were suspended in THF (2 mL), and catalytic amounts of concentrated hydrochloric acid were added. The mixture was stirred at reflux for 18 h. After cooling to room temperature, the solvent was removed in vacuo and the residue was purified by column chromatography on silica gel (cyclohexane/ethyl acetate 10:1 → 5:1 → 2:1 → 1:2). Yield 29 mg (7%); ^{1}H NMR (200 MHz, [D$_6$]DMSO): δ = 2.0 (s, 3H), 2.2 (s, 3H), 5.5 (d, 1H), 7.5 (m, 1H), 7.6 (m, 3H), 7.7 (m, 1H), 7.8 (m, 1H), 7.9 (m, 2H), 8.5 (d, 1H) ppm; MS (ESI+) *m/z*: 400.2 [*M*+H]$^+$.

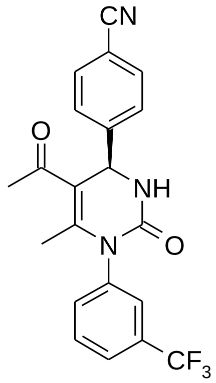

4-{(4*R*)-5-Acetyl-6-methyl-2-oxo-1-[3-(trifluoromethyl)phenyl]-1,2,3,4-tetrahydro-pyrimidin-4-yl}benzonitrile (18): The enantiomers of **S18.1** were separated by preparative HPLC on a chiral phase (column: chiral silica gel phase based on the monomer selector *N*-methacryloyl-L-leucine-1-menthylamide,[8] 250 mm × 20 mm; eluent: ethyl acetate → methanol → ethyl acetate; flow: 25 mL/min; temperature: 23°C; UV detection: 254 nm); $[\alpha]_D^{20}$ = +45.9 (*c* = 0.530 in methanol); ^{1}H NMR (200 MHz, [D$_6$]DMSO): δ = 2.0 (s, 3H), 2.2 (s, 3H), 5.5 (d, 1H), 7.5 (m, 1H), 7.6 (m,

3H), 7.7 (m, 1H), 7.8 (m, 1H), 7.9 (m, 2H), 8.5 (d, 1H) ppm; MS (ESI+) *m*/*z*: 400 $[M+H]^+$.

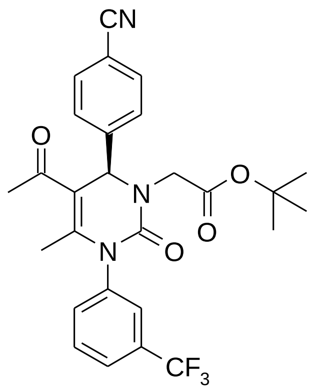

***tert*-Butyl {(6*R*)-5-acetyl-6-(4-cyanophenyl)-4-methyl-2-oxo-3-[3-(trifluoro-methyl)phenyl]-3,6-dihydropyrimidin-1(2*H*)-yl}acetate (S19.1):** The reaction was carried out under argon. To a solution of **18** (15 g, 37.6 mmol) in THF (450 mL), sodium hydride (suspension, 60%, 3.76 g, 93.9 mmol) was added in portions. The mixture was stirred at room temperature for 1 h. *tert*-Butyl bromoacetate (11 g, 56.3 mmol) was added slowly. The mixture was again stirred at room temperature for 1 h. The reaction mixture was poured cautiously into H_2O (500 mL) and brine was added. The mixture was extracted with ethyl acetate (3 ×). The combined organic layers were dried over sodium sulfate and concentrated. The residue was purified by MPLC on silica gel (cyclohexane/dichloromethane 1:1). Yield 9.94 g (51%); ^1H NMR (400 MHz, [D_6]DMSO): δ = 1.25 (s, 9H), 2.00 (s, 3H), 2.20 (s, 3H), 3.95 (d, 1H), 4.15 (d, 1H), 5.70 (s, 1H), 7.60–7.90 (m, 8H) ppm; MS (ESI+) *m*/*z*: 458.1 $[M+H]^+$; MS (ESI-) *m*/*z*: 512.2 $[M-H]^-$.

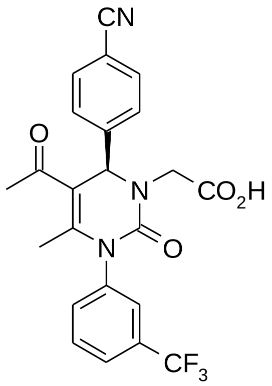

{(6*R*)-5-Acetyl-6-(4-cyanophenyl)-4-methyl-2-oxo-3-[3-(trifluoromethyl)phenyl]--3,6-dihydropyrimidin-1(2*H*)-yl}acetic acid (19): The reaction was carried out under

argon. To a solution of **S19.1** (12.2 g, 23.7 mmol) in dichloromethane (160 mL) was added TFA (36.5 mL, 473.2 mmol). The reaction mixture was stirred at room temperature overnight, and then concentrated. The residue was purified by flash column chromatography on silica gel (dichloromethane → dichloromethane/methanol 100:1 → dichloromethane/methanol 50:1). Yield 7.55 g (70%); ^{1}H NMR (400 MHz, [D$_6$]DMSO): δ = 1.95 (s, 3H), 2.25 (s, 3H), 2.95 (d, 1H), 4.15 (d, 1H), 5.80 (s, 1H), 7.55–7.90 (m, 8H) ppm; MS (ESI+) *m*/*z*: 458.1 [*M*+H]$^+$

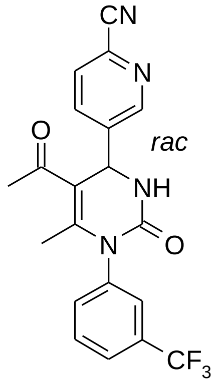

(*rac*)-5-{5-Acetyl-6-methyl-2-oxo-1-[3-(trifluoromethyl)phenyl]-1,2,3,4-tetra-hydropyrimidin-4-yl}pyridine-2-carbonitrile (S20.1): To a stirred solution of 5-formylpyridine-2-carbonitrile (75 mg, 0.57 mmol) in THF (5 mL) was added pentane-2,4-dione (57 mg, 0.57 mmol), 1-[3-(trifluoromethyl)phenyl]urea (116 mg, 0.57 mmol) and polyphosphoric acid ethyl ester (200 mg).[9] The reaction mixture was stirred at reflux for 24 h, then the solution was diluted with DMSO (2 mL) and purified by preparative HPLC (column: YMC Gel ODS-AQ S-5, 250 × 30 mm, 15 μm; eluent: acetonitrile/H_2O gradient, starting with 10% acetonitrile). Yield 101 mg (44%); ^{1}H NMR (200 MHz, [D$_6$]DMSO): δ = 2.02 (s, 3H), 2.24 (s, 3H), 5.54 (d, 1H), 7.52–7.90 (m, 4H), 8.08 (d, 2H), 8.50 (d, 1H), 8.81 (s, 1H) ppm; MS (ESI+) *m*/*z*: 401 [*M*+H]$^+$.

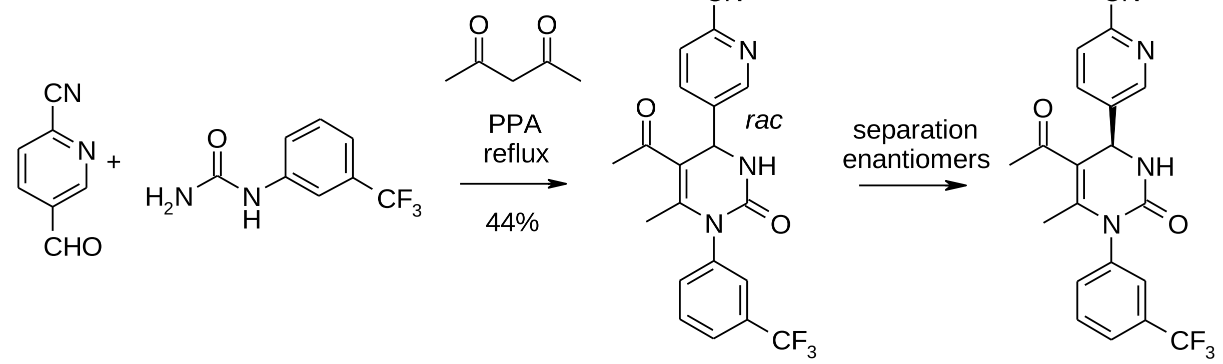

Scheme 4. Representative synthesis for pyrimidinones (Table 4): Synthesis of compound **20** (BAY-678).

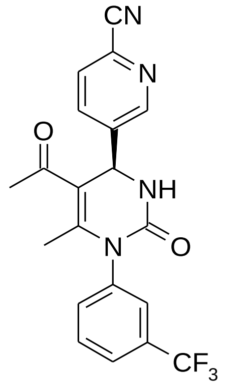

5-{(4*R*)-5-Acetyl-6-methyl-2-oxo-1-[3-(trifluoromethyl)phenyl]-1,2,3,4-tetrahydro-pyrimidin-4-yl}pyridine-2-carbonitrile (BAY-678, **20):** The enantiomers of **S20.1** were separated by preparative HPLC on a chiral phase [column: chiral silica gel phase based on the monomer selector *N*-methacryloyl-L-leucine-1-menthylamide[8], 250 mm × 20 mm, 5 μm; eluent: ethyl acetate → methanol → ethyl acetate; flow: 25 mL/min; temperature: 23°C; UV detection: 254 nm]. $[\alpha]_D^{20}$ = +25.1 (*c* = 0.505 in methanol); ^{1}H NMR (300 MHz, $CDCl_3$): δ = 2.06 (s, 3H), 2.35 (s, 3H), 5.69 (d, 1H), 6.02 (d, 1H), 7.29–7.50 (m, 2H), 7.57–7.75 (m, 3H), 7.83 (dd, 1H), 8.74 (d, 1H) ppm; MS (ESI+) *m*/*z*: 401 $[M+H]^+$.

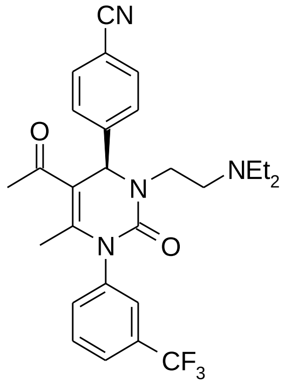

4-{(4*R*)-5-Acetyl-3-[2-(diethylamino)ethyl]-6-methyl-2-oxo-1-[3-(trifluoromethyl)-phenyl]-1,2,3,4-tetrahydropyrimidin-4-yl}benzonitrile (21): The reaction was carried out under argon. To a solution of **18** (5.0 g, 12.5 mmol) was added sodium hydride suspension in mineral oil (60%; 1.25 g, 31.3 mmol). The mixture was stirred at room temperature for 1 h. Then, 2-bromo-*N*,*N*-diethylethylamine hydrobromide

(4.9 g, 18.78 mmol) was added and the reaction mixture was stirred at room temperature overnight. Sodium hydride suspension in mineral oil (60%; 0.75 g, 18.78 mmol) was added again. The mixture was stirred at room temperature for 30 min, and then 2-bromo-*N*,*N*-diethylethylamine hydrobromide (4.9 g, 18.78 mmol) was added again. The mixture was stirred at room temperature for 4 h, then H_2O and brine were added. The mixture was extracted with ethyl acetate (2 ×). The combined organic layers were dried over sodium sulfate and concentrated. The residue was purified by flash chromatography on silica gel (dichloromethane/methanol 80:1 → 50:1 → 30:1). Yield 2.67 g (43%); ^{1}H NMR (400 MHz, [D$_6$]DMSO): δ = 0.87 (t, 6H), 1.76–1.81 (m, 3H), 2.17–2.27 (m, 1H), 2.30–2.43 (m, 4H), 2.53–2.62 (m, 1H), 2.81–2.92 (m, 1H), 3.40–3.51 (m, 1H), 5.60 (s, 1H), 7.66–7.77 (m, 4H), 7.81–7.88 (m, 2H), 7.99 (d, 2H) ppm (3H obscured by solvent signal); MS (ESI+) *m*/*z*: 499.1 [*M*+H]$^+$.

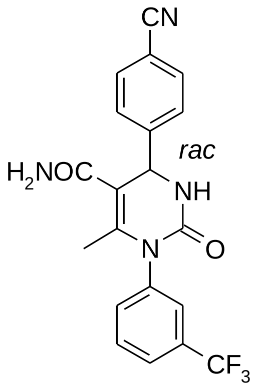

(*rac*)-4-(4-Cyanophenyl)-6-methyl-2-oxo-1-[3-(trifluoromethyl)phenyl]-1,2,3,4-tetrahydropyrimidine-5-carboxamide (S22.1): To a solution of **S17.1** (200 mg, 0.5 mmol) in THF (5 mL) were added 4-(*N*,*N*-dimethylamino)pyridine (6 mg, 0.05 mmol), *N*,*N*-diisopropylethylamine (77 mg, 0.6 mmol) and (benzotriazol-1-yloxy)tripyrrolidinophosphonium hexafluorophosphate (PyBOP; 115 mg, 0.6 mmol). The reaction mixture was stirred at room temperature for 15 min. Ammonia as a 0.5 M solution in dioxane (5 mL, 2.5 mmol) was added. The reaction mixture was stirred at room temperature for 1 h, then H_2O and ethyl acetate were added. The organic layer was dried over sodium sulfate and concentrated to dryness in vacuo. The crude product was purified by preparative HPLC (RP column; eluent: acetonitrile/H_2O + 0.01% TFA-mixtures). Yield 55 mg (28%); ^{1}H NMR (200 MHz, [D$_6$]DMSO): δ = 1.8 (s, 3H), 5.4 (d, 1H), 7.2 (br. s, 1H), 7.4 (br. s, 1H), 7.6 (m, 5H), 7.7 (m, 1H), 7.9 (m, 2H), 8.1 (d, 1H) ppm; MS (ESI+) *m*/*z*: 401 [*M*+H]$^+$.

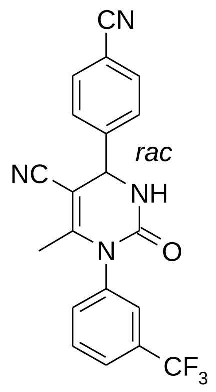

(*rac*)-4-(4-Cyanophenyl)-6-methyl-2-oxo-1-[3-(trifluoromethyl)phenyl]-1,2,3,4-tetrahydropyrimidine-5-carbonitrile (S22.2): To a solution of **S22.1** (0.609 g, 1.52 mmol) in THF (60 mL) was added (methoxycarbonylsulfamoyl)triethyl-ammonium-*N*-betaine (1.24 g, 12.93 mmol). The reaction mixture was stirred at room temperature for 1 h. The solvent was removed in vacuo and the residue was purified by column chromatography on silica gel (dichloromethane/methanol mixtures). Yield 249 mg (43%); ^{1}H NMR (300 MHz, [D$_6$]DMSO): δ = 1.8 (s, 3H), 5.4 (d, 1H), 7.7 (m, 4H), 7.8 (m, 2H), 8.0 (m, 2H), 8.4 (d, 1H) ppm; MS (CI+) *m*/*z*: 400 [*M*+NH$_4$]$^+$.

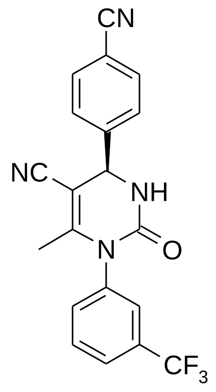

(4*R*)-4-(4-Cyanophenyl)-6-methyl-2-oxo-1-[3-(trifluoromethyl)phenyl]-1,2,3,4-tetrahydropyrimidine-5-carbonitrile (22): The enantiomers of **S22.2** were separated by preparative HPLC on a chiral phase (column: chiral silica gel phase based on the monomer selector *N*-methacryloyl-L-leucine-1-menthylamide,[8] 250 mm × 20 mm, 10 μm; eluent: ethyl acetate → methanol → ethyl acetate; flow 25 mL/min; temperature: 23°C; UV detection: 254 nm). $[\alpha]_D^{20}$ = –179 (*c* = 0.530 in methanol); ^{1}H NMR (300 MHz, [D$_6$]DMSO): δ = 1.8 (s, 3H), 5.4 (d, 1H), 7.7 (m, 4H), 7.8 (m, 2H), 8.0 (m, 2H), 8.4 (d, 1H) ppm; MS (ESI+) *m*/*z*: 383.1 [*M*+H]$^+$.

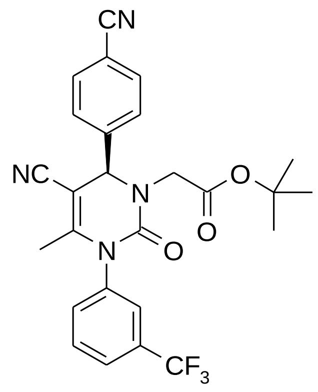

***tert*-Butyl {(6*R*)-5-cyano-6-(4-cyanophenyl)-4-methyl-2-oxo-3-[3-(trifluoro-methyl)phenyl]-3,6-dihydropyrimidin-1(2*H*)-yl}acetate (S23.1):** To a solution of **22** (5900 mg, 15.431 mmol) in DMF (150 mL) were added potassium carbonate (7677 mg, 55.55 mmol) and *tert*-butyl bromoacetate (7224 mg, 37.03 mmol). The reaction mixture was stirred at 60°C overnight. Then, the solvent was removed in a rotary evaporator, and the residue was taken up in a mixture of ethyl acetate and H_2O. The organic phase was separated, dried over sodium sulfate, filtered and concentrated. The residue was purified by MPLC on silica gel (cyclohexane/ethyl acetate 1:2). Yield 5570 mg (73%); LC-MS (method 8): t_R = 2.65 min; ^1H NMR (400 MHz, [D_6]DMSO): δ = 1.29 (s, 9H), 1.86 (s, 3H), 3.71 (d, 1H), 3.96 (d, 1H), 5.49 (s, 1H), 7.70–7.75 (m, 2H), 7.78 (d, 2H), 7.84 (d, 1H), 7.87 (s, 1H), 7.96 (d, 2H) ppm; MS (ESI–) *m*/*z*: 495.2 [*M*–H]$^-$.

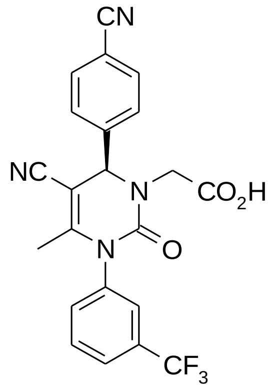

{(6*R*)-5-Cyano-6-(4-cyanophenyl)-4-methyl-2-oxo-3-[3-(trifluoromethyl)phenyl]-3,6-dihydropyrimidin-1(2*H*)-yl}acetic acid (S23.2): To a solution of **S23.1** (5840 mg, 11.76 mmol) in dichloromethane (40 mL) was added TFA (13.412 g, 117.62 mmol). The mixture was stirred at 50°C for 5 h. The volatile constituents were then removed in a rotary evaporator. The residue was purified by MPLC on silica gel

(dichloromethane/methanol 10:1). Yield 3120 mg (58%); LC-MS (method 9): t_R = 2.47 min; ^{1}H NMR (400 MHz, [D$_6$]DMSO): δ = 1.84 (s, 3H), 3.55 (d, 1H), 4.07 (d, 1H), 5.51 (s, 1H), 7.69–7.81 (m, 4H), 7.84–7.90 (m, 2H), 7.96 (d, 2H), 12.78 (br. s, 1H) ppm; MS (ESI+) *m*/*z*: 441.1 [*M*+H]$^+$.

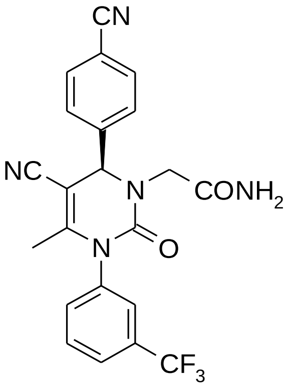

2-{(6*R*)-5-Cyano-6-(4-cyanophenyl)-4-methyl-2-oxo-3-[3-(trifluoromethyl)-phenyl]-3,6-dihydropyrimidin-1(2*H*)-yl}acetamide (23): A solution of compound **S23.2** (100 mg, 0.23 mmol) in THF (5 mL) was cooled to –20°C. *N*-Methylmorpholine (57 mg, 0.57 mmol) and ethyl chloroformate (62 mg, 0.57 mmol) were added. The mixture was stirred at –20°C for 30 min. A mixture of 35% aqueous ammonia solution (0.15 mL) in THF (1.5 mL) was added. The reaction mixture was then allowed to slowly reach room temperature, and the contents of the flask were then added to 3 N hydrochloric acid. The aqueous phase was extracted with ethyl acetate and separated off. The organic phase was dried over sodium sulfate, filtered and concentrated. The residue was purified by preparative HPLC (instrument: Abimed Gilson Pump 305/306, Manometric Module 806; column: GromSil C-18, 250 mm × 30 mm, 10 μm; eluent A: H_2O + 0.1% TFA, eluent B: acetonitrile; gradient: 0–3 min 10% B, 3.01–34 min 95% B, 34.01–38 min 95% B, 38.01–40 min 10% B; flow: 50 mL/min; UV detection: 210 nm). Yield 44 mg (44%); HPLC (method 10): t_R = 4.19 min; ^{1}H NMR (400 MHz, [D$_6$]DMSO): δ = 1.82 (s, 3H), 3.20 (d, 1H), 4.10 (d, 1H), 5.45 (s, 1H), 7.12 (s, 1H), 7.39 (s, 1H), 7.72–7.76 (m, 4H), 7.83 (d, 1H), 7.87 (s, 1H), 7.89 (d, 2H) ppm; MS (CI+) *m*/*z*: 440 [*M*+H]$^+$, 457 [*M*+NH_4]$^+$.

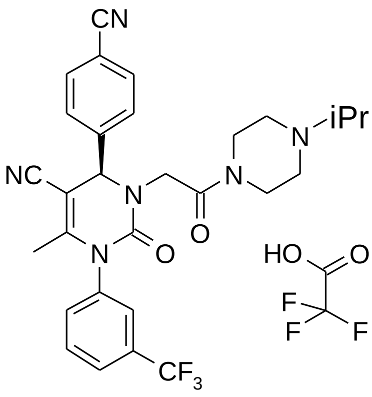

(4*R*)-4-(4-Cyanophenyl)-3-[2-(4-isopropylpiperazin-1-yl)-2-oxoethyl]-6-methyl-2-oxo-1-[3-(trifluoromethyl)phenyl]-1,2,3,4-tetrahydropyrimidine-5-carbonitrile trifluoroacetate (24): The reaction was carried out under argon. **S23.2** (110 mg (90% puritiy), 0.22 mmol) was dissolved in DMF (0.5 mL) and cooled to 0°C. 1-[Bis(dimethylamino)methylene]-1*H*-1,2,3-triazolo[4,5-*b*]pyridinium 3-oxid hexa-fluorophosphate (HATU; 171 mg, 0.45 mmol, 2 eq.) was added and the mixture was stirred for 20 min. Then, 1-isopropylpiperazine (86.5 mg, 0.67 mmol, 3 eq.) and *N*,*N*-diisopropylethylamine (78 µL, 0.45 mmol, 2 eq.) were added. The reaction mixture was stirred at room temperature for 90 min, and was then purified by preparative HPLC (RP-column; eluent: acetonitrile/H_2O + 0.1% TFA-mixtures).Yield 98 mg (66%); ^{1}H NMR (400 MHz, [D$_6$]DMSO): δ = 1.23 (d, 6H), 1.85 (s, 3H), 2.93 (br. s, 2H), 3.16–3.64 (m, 3H), 4.42–4.65 (m, 2H), 5.36 (s, 1H), 7.65–7.79 (m, 4H), 7.81–7.86 (m, 2H), 7.99 (d, 2H) ppm (2 signals not visible); MS (CI+) *m*/*z*: 551 [*M*+H]$^+$.

Synthesis of 25–27

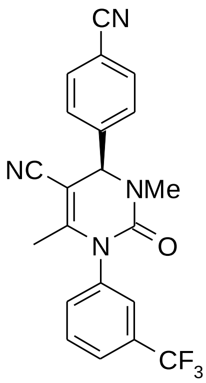

(4*R*)-4-(4-Cyanophenyl)-3,6-dimethyl-2-oxo-1-[3-(trifluoromethyl)phenyl]-1,2,3,4-tetrahydropyrimidine-5-carbonitrile (25): The reaction was carried out under argon. **22** (20 mg, 0.05 mmol) was dissolved in absolute THF and cooled to –78°C.

Lithium bis(trimethylsilyl)amide solution (LiHMDS; 1 M in THF; 0.1 mL, 0.11 mmol, 2 eq.) was added dropwise. After stirring at –78°C for 15 min, methyl iodide (37 mg, 0.26 mmol, 5 eq.) was added. The reaction mixture was stirred at –78°C for 90 min, and then at room temperature for 16 h. Saturated aqueous ammonium chloride solution (20 mL) and H_2O were added. The mixture was extracted with ethyl acetate (3 × 15 mL). The combined organic layers were dried over sodium sulfate and concentrated to dryness in vacuo. The residue was purified by preparative HPLC (column: Kromasil-100A C-18, 250 mm × 4.6 mm, 5 µm; eluent: acetonitrile/H_2O + 0.1% TFA 10:90 → 90:10). Yield 20 mg (96%); ^{1}H NMR (400 MHz, $CDCl_3$): δ = 1.89 (s, 3H), 2.93 (s, 3H), 5.11 (s, 1H), 7.50 (d, 3H), 7.64 (t, 1H), 7.74 (d, 1H), 7.80 (d, 2H) ppm (1H obscured by solvent peak); MS (ESI+) *m*/*z*: 397.3 [*M*+H]$^+$.

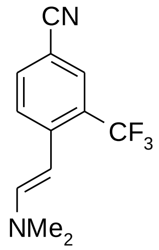

4-[(*E*)-2-(Dimethylamino)vinyl]-3-(trifluoromethyl)benzonitrile (S26.1): The reaction was carried out under argon. 4-Methyl-3-(trifluoromethyl)benzonitrile (20 g, 108 mmol) was dissolved in DMF (130 mL). Dimethylformamide dimethyl acetal (17.8 g, 140.4 mmol) was added and the reaction mixture was heated at 140°C for 20 h. The reaction mixture was concentrated to remove DMF. The crude product was used for the next step. Yield 29 g (111%, crude).

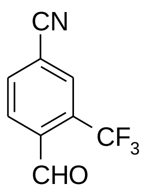

4-Formyl-3-(trifluoromethyl)benzonitrile (S26.2): S26.1 (29 g (crude), 101.6 mmol) was dissolved in a mixture of H_2O/THF (1:1, 430 mL); sodium periodate (65.17 g, 304.7 mmol) was added and the mixture was stirred at room temperature for 1 h. The precipitate was collected by filtration and the solid was washed with ethyl acetate. The filtrate phases were separated. The organic layer was washed with saturated aqueous sodium bicarbonate solution (3 ×) and brine (2 ×), dried over sodium sulfate

and concentrated. The residue was purified by column chromatography on silica gel (cyclohexane → cyclohexane/ethyl acetate 4:1). Yield 10.3 g (51%); ^{1}H NMR (400 MHz, [D$_6$]DMSO): δ = 8.23 (dd, 1H), 8.40 (d, 1H), 8.51 (s, 1H), 10.28–10.31 (m, 1H) ppm; MS (ESI+) *m*/*z*: 200 [*M*+H]$^+$.

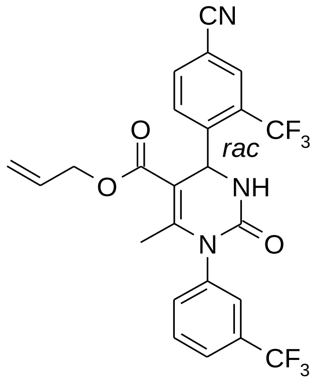

Allyl (*rac*)-4-[4-cyano-2-(trifluoromethyl)phenyl]-6-methyl-2-oxo-1-[3-(trifluoromethyl)phenyl]-1,2,3,4-tetrahydropyrimidine-5-carboxylate (S26.3): The reaction was carried out under argon. Allyl acetoacetate (4.64 g, 32.64 mmol) was dissolved in THF (160 mL). **S26.2** (6.5 g, 32.64 mmol), 1-[3-(trifluoromethyl)phenyl]urea (6.66 g, 32.64 mmol) and poly(2,6-dimethyl-1,4-phenylene oxide) (12.8 g) were added. The reaction mixture was heated at reflux for 19 h. The organic solvent was removed. The residue was dissolved in ethyl acetate (300 mL) and washed with saturated aqueous sodium bicarbonate solution (100 mL) and brine (100 mL), dried over sodium sulfate and concentrated. The residue was purified by column chromatography on silica gel (cyclohexane → cyclohexane/ethyl acetate 2:1). Yield 9.8 g (59%); ^{1}H NMR (400 MHz, [D$_6$]DMSO): δ = 2.11 (s, 3H), 4.33–4.45 (m, 2H), 4.87 (dd, 1H), 5.00 (dd, 1H), 5.54–5.65 (m, 1H), 5.75–5.78 (m, 1H), 7.71–7.76 (m, 2H), 7.79–7.84 (m, 1H), 7.87–7.92 (m, 1H), 8.09–8.15 (m, 2H), 8.21–8.25 (m, 1H), 8.28–8.30 (m, 1H) ppm; MS (ESI+) *m*/*z*: 510 [*M*+H]$^+$.

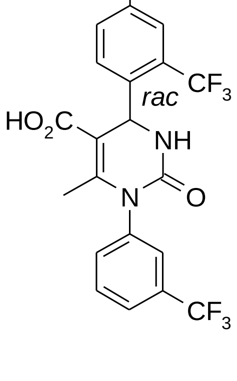

(*rac*)-4-[4-Cyano-2-(trifluoromethyl)phenyl]-6-methyl-2-oxo-1-[3-(trifluoromethyl)phenyl]-1,2,3,4-tetrahydropyrimidine-5-carboxylic acid (S26.4): The reaction was carried out under argon. **S26.3** (1.04 g, 2.04 mmol) and morpholine (266.8 mg, 3.06 mmol, 1.5 eq.) were dissolved in anhydrous THF (25 mL) at room temperature. Tetrakis(triphenylphosphine)palladium(0) (118 mg, 0.1 mmol, 0.05 eq.) was added. The mixture was stirred at room temperature for 20 h. Then, it was diluted with ethyl acetate (200 mL) and washed with aqueous hydrogen chloride solution (2 × 70 mL) and brine (50 mL). The organic layer was dried over sodium sulfate and concentrated to dryness in vacuo. The crude product was used without further purification for the next step. Yield 1.04 g (108%, crude); ^{1}H NMR (400 MHz, [D$_6$]DMSO): δ = 2.10 (s, 3H), 5.72 (d, 1H), 7.69–7.75 (m, 2H), 7.77–7.83 (m, 1H), 7.87 (s, 1H), 7.99 (d, 1H), 8.09 (d, 1H), 8.22 (d, 1H), 8.28 (s, 1H), 12.35 (s, 1H) ppm; MS (ESI+) *m*/*z*: 470.3 [*M*+H]$^+$.

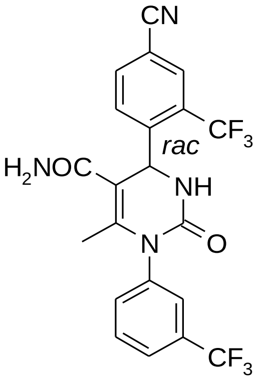

(*rac*)-4-[4-Cyano-2-(trifluoromethyl)phenyl]-6-methyl-2-oxo-1-[3-(trifluoromethyl)phenyl]-1,2,3,4-tetrahydropyrimidine-5-carboxamide (S26.5): The reaction was carried out under argon. **S26.4** (800 mg (crude)) and 1-[bis(dimethylamino)methylene]-1*H*-1,2,3-triazolo[4,5-*b*]pyridinium 3-oxid hexafluorophosphate (HATU; 1.3 g, 3.4 mmol) were dissolved in anhydrous DMF at 0°C. After 20 min, ammonium chloride (455.9 mg, 8.5 mmol) and *N*,*N*-diisopropylethylamine (2.1 mL, 11.93 mmol) were added. The mixture was stirred at room temperature for 90 min, then was purified directly in 3 portions by preparative HPLC (column: GromSil C-18, 10 µm; eluent: acetonitrile/H_2O + 0.1% TFA 10:90 → 90:10). Yield 540 mg (68% (if the starting material would be 100%)); ^{1}H NMR (400 MHz, [D$_6$]DMSO): δ = 1.72 (s, 3H), 5.64–5.69 (m, 1H), 7.64–7.81 (m, 4H), 7.89–7.93 (m, 1H), 8.10–8.15 (m, 1H), 8.25–8.30 (m, 2H) ppm (2H not visible); MS (ESI+) *m*/*z*: 469.1 [*M*+H]$^+$.

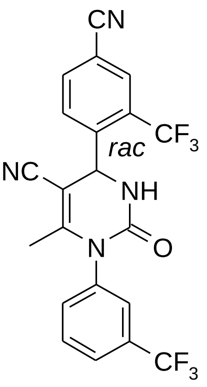

(*rac*)-4-[4-Cyano-2-(trifluoromethyl)phenyl]-6-methyl-2-oxo-1-[3-(trifluoromethyl)phenyl]-1,2,3,4-tetrahydropyrimidine-5-carbonitrile (S26.6): The reaction was carried out under argon. **S26.5** (400 mg, 0.85 mmol) was dissolved in anhydrous THF (10 mL). (Methoxycarbonylsulfamoyl)triethylammonium hydroxide (Burgess reagent; 814 mg, 3.42 mmol, 4 eq.) was added and the mixture was stirred at room temperature for 60 min. The solvent was removed in vacuo. The residue was purified by column chromatography on silica gel (cyclohexane/ethyl acetate 1:1). Yield 360 mg (94%); ^{1}H NMR (400 MHz, [D$_6$]DMSO): δ = 1.82 (d, 3H), 5.65 (s, 1H), 7.70–7.78 (m, 1H), 7.80–7.86 (m, 1H), 8.36 (s, 2H), 8.51 (d, 1H) ppm (3 protons were not assigned); MS (ESI+) *m*/*z*: 451.2 [*M*+H]$^+$.

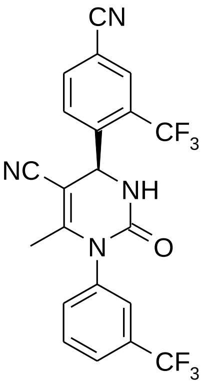

(4*R*)-4-[4-Cyano-2-(trifluoromethyl)phenyl]-6-methyl-2-oxo-1-[3-(trifluoromethyl)phenyl]-1,2,3,4-tetrahydropyrimidine-5-carbonitrile (26): S26.6 (340 mg) was separated into the enantiomers by preparative HPLC on a chiral phase [column: Daicel Chiralpak IB, 250 × 20 mm, 5 µm; sample preparation: solution in methanol/*tert*-butyl methyl ether (MTBE) (3:13, 31 mL); injection volume: 0.5 mL; eluent: MTBE/methanol 88:12; flow: 15 mL/min; temperature: 30°C; UV detection: 220 nm]. Yield 166 mg (98.5% purity, >99.5% ee); ^{1}H NMR (400 MHz, [D$_6$]DMSO): δ = 1.82 (d, 3H), 5.65 (br. s, 1H), 7.70–7.78 (m, 1H), 7.80–7.86 (m, 1H), 8.33–8.39 (m,

2H), 8.49–8.54 (m, 1H) ppm (3 protons were not assigned); MS (ESI+) *m*/*z*: 451.0 [*M*+H]$^+$.

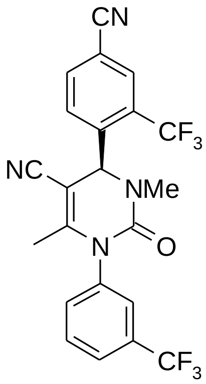

(4*R*)-4-[4-Cyano-2-(trifluoromethyl)phenyl]-3,6-dimethyl-2-oxo-1-[3-(trifluoromethyl)phenyl]-1,2,3,4-tetrahydropyrimidine-5-carbonitrile (27): The reaction was carried out under argon. **26** (100 mg, 0.222 mmol) was dissolved in absolute THF (10 mL) and cooled to –78°C. Lithium bis(trimethylsilyl)amide solution (LiHMDS; 1 M in THF; 0.27 mmol, 0.27 ml, 1.2 eq.) was added dropwise. After stirring for 20 min, methyl iodide (158 mg, 1.11 mmol, 5 eq.) was added. The mixture was allowed to warm to room temperature. Saturated aqueous ammonium chloride solution (50 mL) was added. The mixture was extracted with ethyl acetate (3 × 50 mL). The combined organic layers were dried over sodium sulfate and concentrated to dryness. The crude product was purified by column chromatography on silica gel (cyclohexane → cyclohexane/ethyl acetate 5:1). The product-containing fractions were concentrated and purified by preparative HPLC (column: GromSil C-18, 10 μm; eluent: acetonitrile/H_2O + 0.1% TFA 10:90 → 75:25). Yield 92 mg (89%); ^{1}H NMR (400 MHz, [D_6]DMSO): δ = 1.81 (s, 3H), 2.63 (s, 3H), 5.65 (s, 1H), 7.60–8.43 (m, 7H) ppm; MS (ESI+) *m*/*z*: 465.0 [*M*+H]$^+$.

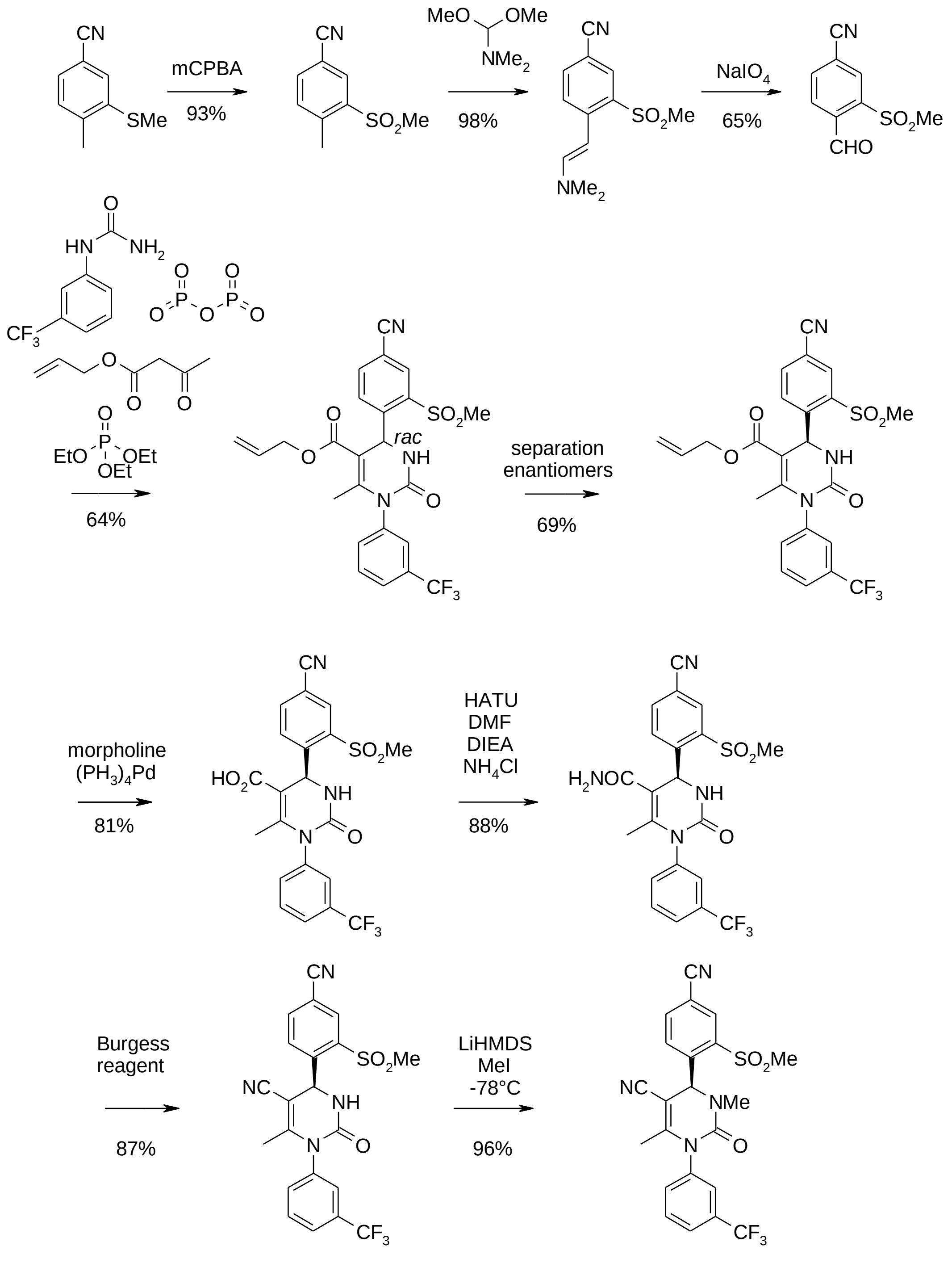

Scheme 5. Synthesis of compound **29** (BAY 85-8501).

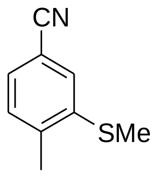

Method A: **4-Methyl-3-(methylsulfanyl)benzonitrile (31):** The reaction was carried out under argon. 3-Fluoro-4-methylbenzonitrile (3.00 g, 22.2 mmol) and sodium thiomethylate (1.57 g, 20.2 mmol) were dissolved in DMF (30 mL), and potassium

carbonate (6.97 g, 50.5 mmol) was added. The reaction mixture was heated at reflux overnight, then was concentrated. The residue was suspended in dichloromethane/methanol (10:1) and filtered. The fitrate was concentrated. The residue was purified by column chromatography on silica gel (cyclohexane/ethyl acetate 10:1). Yield 2.51 g (64% based on 3-fluoro-4-methylbenzonitrile).

Method B: **4-Methyl-3-(methylsulfanyl)benzonitrile (31):** To avoid exposure to sulfur compounds, all gas emissions of this reaction were washed with aqueous sodium hypochlorite solution. 3-Fluoro-4-methylbenzonitrile (200 g, 1.48 mol) was dissolved in DMF (1.5 L) and the solution was heated to 40°C. Sodium thiomethylate (126.8 g, 1.63 mol) was added portionwise (25 g each step); the temperature rose to 100°C during the addition. The reaction mixture was stirred at 175°C (bath temperature) for 1.5 h, then at room temperature overnight. Then, the mixture was poured into H_2O (7.5 L) and extracted with ethyl acetate (2 × 1.875 L). The combined organic layers were washed with brine (1.875 L) and concentrated, and the residue was purified by column chromatography on silica gel (petroleum ether/ethyl acetate 95:5). Yield 172 g (71%); GC-MS (method 11): t_R = 5.25 min; GC-MS (method 11): t_R = 5.25 min; ^{1}H NMR (400 MHz, [D$_6$]DMSO): δ = 2.30 (s, 3H), 2.54 (s, 3H), 7.38 (d, 1H), 7.52 (dd, 1H), 7.58 (br. s, 1H) ppm; MS (ESI+) *m*/*z*: 163 [*M*+H]$^+$.

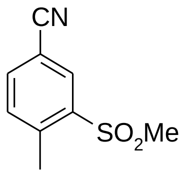

Method A: **4-Methyl-3-(methylsulfonyl)benzonitrile (32): 31** (14.05 g, 86.1 mmol) was dissolved in dichloromethane (700 mL) and cooled to 0°C. 3-Chloroperoxybenzoic acid (50.9 g, 206.6 mmol) was added slowly. The mixture was stirred at 0°C for 40 min and at room temperature overnight. The precipitated 3-chloroperoxybenzoic acid was removed by filtration. The filtrate was washed with 1 N aqueous sodium hydroxide solution. The organic layer was dried over sodium sulfate and concentrated. The residue was purified by column chromatography on silica gel (cyclohexane/ethyl acetate 1:1 → 1:2). Yield 13.65 g (81%).

Method B: **4-Methyl-3-(methylsulfonyl)benzonitrile (32):** 3-Chloroperoxybenzoic acid (2.501 kg, 10.14 mol) was dissolved in dichloromethane (27.2 L) and cooled to 10°C. **31** (552 g, 3.38 mol) was added portionwise. The reaction mixture was stirred

at room temperature for 5 h. The precipitated 3-chloroperoxybenzoic acid was removed by filtration and the solid was washed with dichloromethane (3 L). The combined filtrates were stirred with 1 N aqueous sodium hydroxide solution (15 L). The mixture was filtered and the organic layer was separated. The organic layer was stirred again with 1 N aqueous sodium hydroxide solution (15 L), separated from the aqueous layer, dried (e.g. over sodium sulfate) and concentrated. The residue was suspended in diethyl ether (4 L), stirred for 10 min and filtered. The collected solid was washed with diethyl ether and dried under high vacuum. Yield 613 g (93%); GC-MS (method 11): t_R = 6.59 min; ^{1}H NMR (400 MHz, [D$_6$]DMSO): δ = 2.30 (s, 3H), 2.54 (s, 3H), 7.38 (d, 1H), 7.52 (dd, 1H), 7.58 (br. s, 1H) ppm; MS (ESI+) *m*/*z*: 195.0 [*M*]$^+$.

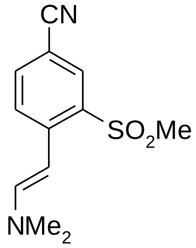

Method A: **4-[2-(Dimethylamino)vinyl]-3-(methylsulfonyl)benzonitrile (33):** The reaction was carried out under argon. **32** (13 g, 66.6 mmol) and N,N-dimethylformamide dimethyl acetal (10.32 g, 86.6 mmol) were dissolved in DMF (200 mL) and heated at 140°C for 14 h. N,N-dimethylformamide dimethyl acetal (3.97 g, 33.3 mmol) was added again, and the mixture was heated at 140°C for a further 24 h. The reaction mixture was concentrated to remove DMF. The residue was used for the next step.

Method B: **4-[2-(Dimethylamino)vinyl]-3-(methylsulfonyl)benzonitrile (33):** The reaction was carried out under argon. **32** (612 g, 3.13 mol) was dissolved in DMF (6.12 L), *N,N*-dimethylformamide dimethyl acetal (859 g, 7.21 mmol) was added. The mixture was heated at 140°C for 7 h, then was poured into an aqueous 10% sodium chloride solution (35 L) and extracted with ethyl acetate (2 × 10 L). The combined organic layers were washed with brine, dried (e.g. over sodium sulfate) and concentrated. The residue was dried under high vacuum overnight. Yield 1098 g (98%, ~70% purity); GC-MS (method 11): t_R = 8.95 min; MS (ESI+) *m*/*z*: 250 [*M*+H]$^+$.

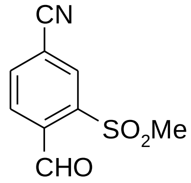

Method A: **4-Formyl-3-(methylsulfonyl)benzonitrile (34): 33** (16.67 g, 66.6 mmol) was dissolved in a mixture of H_2O/THF (1:1, 500 mL); sodium periodate (42.72 g, 199.7 mmol) was added and the mixture was stirred at room temperature overnight. The precipitate was collected by filtration and the solid was washed with ethyl acetate. The combined organic layers were washed with saturated aqueous sodium bicarbonate solution and brine, dried over sodium sulfate and concentrated. The residue was purified by column chromatography on silica gel (cyclohexane/ethyl acetate 1:1). Yield 4.6 g (33%).

Method B: **4-Formyl-3-(methylsulfonyl)benzonitrile (34): 33** (1098 g, 3.07 mol, ~70% purity) was dissolved in a mixture of H_2O/THF (1:1, 13.8 L); sodium periodate (1970 g, 9.21 mol) was added and the mixture was stirred at room temperature for 1 h. The precipitate was collected by filtration and the solid was washed with ethyl acetate (17 L). To the combined organic filtrates was added H_2O (17 L). The phases were separated. The organic layer was washed with saturated aqueous sodium bicarbonate solution (8.5 L) and brine (8.5 L), dried (e.g. over sodium sulfate) and concentrated. The residue was purified by column chromatography on silica gel (dichloromethane/ethyl acetate 9:1; 60 L). The product-containing fractions were suspended in petroleum ether and filtered. The solid was dried under high vacuum overnight. Yield 436 g (65%); GC-MS (method 11): t_R = 6.89 min; ^{1}H NMR (400 MHz, [D$_6$]DMSO): δ = 3.57 (s, 3H), 8.10 (d, 1H), 8.39 (dd, 1H), 8.45 (d, 1H), 10.63 (s, 1H) ppm; MS (ESI+) *m*/*z*: 227 [*M*+18]$^+$.

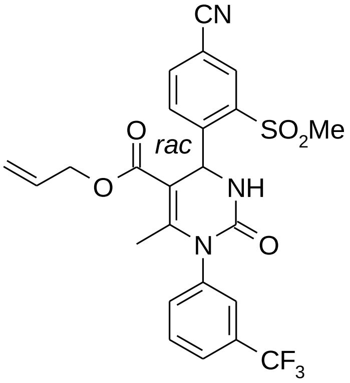

Allyl (*rac*)-4-[4-cyano-2-(methylsulfonyl)phenyl]-6-methyl-2-oxo-1-[3-(trifluoro-methyl)phenyl]-1,2,3,4-tetrahydropyrimidine-5-carboxylate (35): The reaction was carried out under argon. Triethyl phosphate (22.98 g, 126 mmol) and phosphorus pentoxide (11.94 g, 84.1 mmol) were stirred at 50°C overnight. The mixture was diluted with *tert*-butyl methyl ether (MTBE; 450 mL). **34** (22.00 g, 105 mmol), 1-[3-(trifluoromethyl)phenyl]urea (21.47 g, 105 mmol) and allyl acetoacetate (22.42 g, 158 mmol) were added. The reaction mixture was heated at reflux overnight. MTBE (350 mL) was removed by distillation. The residue was heated at reflux for a further 4 h. The organic solvents were removed. The residue was suspended in diethyl ether and filtered. The solid was washed with H_2O (350 mL) and diethyl ether (50 mL). Yield 34.74 g (64%); LC-MS (method 13): t_R = 1.28 min; ^{1}H NMR (400 MHz, [D$_6$]DMSO): δ = 2.15 (s, 3H), 3.45 (s, 3H), 4.45 (m, 2H), 4.95 (d, 1H), 5.05 (d, 1H), 5.65 (m, 1H), 6.40 (d, 1H), 7.20 (d, 1H), 7.70 (m, 2H), 7.80 (m, 1H), 7.85 (br. s, 1H), 8.10 (br. d, 1H), 8.25 (d, 1H), 8.35 (s, 1H) ppm; MS (ESI+) *m*/*z*: 520.2 $[M+H]^+$.

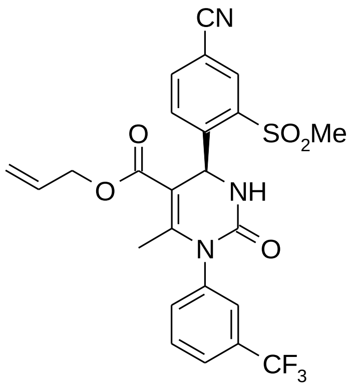

Allyl (4*S*)-4-[4-cyano-2-(methylsulfonyl)phenyl]-6-methyl-2-oxo-1-[3-(trifluoro-methyl)phenyl]-1,2,3,4-tetrahydropyrimidine-5-carboxylate (36): 35 (2.33 g) was separated into the enantiomers by preparative HPLC on a chiral phase [column: chiral silica gel phase based on the selector poly(*N*-methacryloyl-D-leucine-di-cyclopropylmethylamide), 600 mm × 30 mm, 10 µm; sample preparation: each 1 g of the sample was dissolved in THF/ethyl acetate/isohexane (20:25:25, 70 mL); injec-tion volume: 8 mL; eluent: isohexane/isopropanol 1:1; flow: 60 mL/min; temperature: 24°C; UV detection: 260 nm]. Yield 0.8 g (69%, >99.5% ee); LC-MS (method 14): t_R = 2.11 min; chiral analytical HPLC [column: chiral silica gel phase based on the selector poly(*N*-methacryloyl-D-leucine-dicyclopropylmethylamide), 250 mm × 4.6 mm, 5 µm; eluent: isohexane/ethyl acetate 1:1; flow: 2 mL/min; UV detection:

260 nm]: t_R = 1.45 min; $[\alpha]_D^{20}$ = +41.6 (*c* = 0.485 in methanol); ^{1}H NMR (400 MHz, [D$_6$]DMSO): δ = 2.10 (s, 3H), 3.45 (s, 3H), 4.45 (m, 2H), 4.95 (d, 1H), 5.05 (d, 1H), 5.65 (m, 1H), 6.40 (d, 1H), 7.20 (d, 1H), 7.70 (m, 2H), 7.80 (m, 1H), 7.85 (br. s, 1H), 8.10 (br. d, 1H), 8.25 (d, 1H), 8.35 (s, 1H) ppm; MS (ESI+) *m*/*z*: 520.1 $[M+H]^+$; MS (ESI–) *m*/*z*: 518.2 $[M-H]^-$.

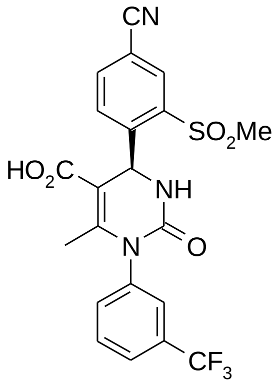

(4*S*)-4-[4-Cyano-2-(methylsulfonyl)phenyl]-6-methyl-2-oxo-1-[3-(trifluoro-methyl)phenyl]-1,2,3,4-tetrahydropyrimidine-5-carboxylic acid (37): The reaction was carried out under argon. **36** (800 mg, 1.54 mmol) and morpholine (201 mg, 2.31 mmol, 1.5 eq.) were dissolved in anhydrous THF (25 mL) at room temperature. Tetrakis(triphenylphosphine)palladium(0) (89 mg, 0.077 mmol, 0.05 eq.) was added. The mixture was stirred at room temperature for 90 min, then concentrated. The residue was dissolved in ethyl acetate (500 mL) and washed with saturated aqueous ammonium chloride solution (50 mL), H_2O (50 mL) and brine (50 mL). The organic layer was dried over sodium sulfate and concentrated. The residue was purified by preparative HPLC (column: GromSil C-18, 10 µm; eluent: acetonitrile/H_2O + 0.1% TFA 10:90 → 90:10). Yield 696 mg (94%); LC-MS (method 9): t_R = 2.15 min; ^{1}H NMR (400 MHz, [D$_6$]DMSO): δ = 2.14 (s, 3H), 3.45 (s, 3H), 6.35 (d, 1H), 7.14 (d, 1H), 7.72 (m, 2H), 7.80 (m, 1H), 7.86 (s, 1H), 8.11 (d, 1H), 8.27 (d, 1H), 8.36 (s, 1H), 12.64 (br. s, 1H) ppm; MS (ESI+) *m*/*z*: 480.1 $[M+H]^+$; MS (ESI–) *m*/*z*: 478.1 $[M-H]^-$.

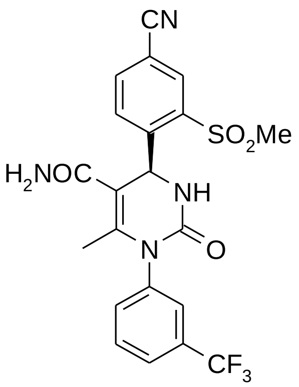

(4*S*)-4-[4-Cyano-2-(methylsulfonyl)phenyl]-6-methyl-2-oxo-1-[3-(trifluoro-methyl)phenyl]-1,2,3,4-tetrahydropyrimidine-5-carboxamide (38): The reaction was carried out under argon. **37** (696 mg, 1.45 mmol) and 1-[bis(di-methylamino)methylene]-1*H*-1,2,3-triazolo[4,5-*b*]pyridinium 3-oxid hexafluoro-phosphate (HATU; 1.1 g, 2.9 mmol, 2 eq.) were dissolved in anhydrous DMF (35 mL) at 0°C. After 20 min, ammonium chloride (388 mg, 7.26 mmol, 5 eq.) and *N*,*N*-diisopropylethylamine (1.31 g, 10.16 mmol, 7 eq.) were added. The mixture was stirred at room temperature for 4 h, then was concentrated. The residue was purified by preparative HPLC (column: GromSil C-18, 10 µm; eluent: aceto-nitrile/H_2O + 0.1% TFA 10:90 → 90:10). Yield 612 mg (88%); LC-MS (method 9): t_R = 1.94 min; ^1H NMR (400 MHz, [D_6]DMSO): δ = 1.80 (s, 3H), 3.40 (s, 3H), 6.35 (s, 1H), 7.20 (s, 1H), 7.25 (br. s, 1H), 7.45 (br. s, 1H), 7.65–7.80 (m, 4H), 8.10 (d, 1H), 8.30 (s, 1H), 8.35 (d, 1H) ppm; MS (ESI+) *m*/*z*: 479.1 $[M+H]^+$; MS (ESI–) *m*/*z*: 477 $[M-H]^-$.

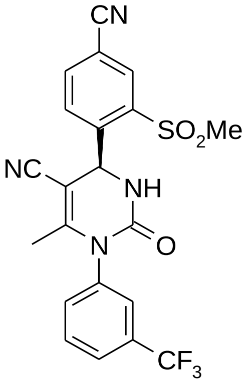

Method A: **(4*S*)-4-[4-Cyano-2-(methylsulfonyl)phenyl]-6-methyl-2-oxo-1-[3-(tri-fluoromethyl)phenyl]-1,2,3,4-tetrahydropyrimidine-5-carbonitrile (28):** The reaction was carried out under argon. **38** (560 mg, 1.17 mmol) was dissolved in anhydrous THF (35 mL). (Methoxycarbonylsulfamoyl)triethylammonium hydroxide (Burgess reagent; 1115 mg, 4.68 mmol, 4 eq.) was added and the mixture was stirred at room temperature for 90 min. The solvent was removed in vacuo. The

residue was purified by preparative HPLC (column: GromSil C-18, 10 μm; eluent: acetonitrile/H_2O + 0.1% TFA 10:90 → 90:10). Yield 470 mg (87%).

Method B: **(4*S*)-4-[4-Cyano-2-(methylsulfonyl)phenyl]-6-methyl-2-oxo-1-[3-(trifluoromethyl)phenyl]-1,2,3,4-tetrahydropyrimidine-5-carbonitrile (28): 38** (10.4 g, 21.7 mmol) and TEA (5.63 g, 55.6 mmol) were dissolved in anhydrous THF (50 mL). Trifluoroacetic anhydride (11.69 g, 55.6 mmol) was added dropwise, ensuring a temperature below 35°C. The reaction mixture was stirred at room temperature for 15 min. Saturated aqueous sodium bicarbonate solution (250 mL) was added dropwise. The mixture was extracted with ethyl acetate (2 ×). The combined organic layers were washed with brine and dried over magnesium sulfate. Silica gel (30 g) was added and the mixture was concentrated. The residue was purified by column chromatography on silica gel (dichloromethane/ethyl acetate 2:1). Yield 6.46 g (65%); LC-MS (method 9): t_R = 2.28 min; mp: 258–259°C; $[\alpha]_D^{20}$ = –222.0 (*c* = 0.480 in DMF); ^{1}H NMR (400 MHz, [D_6]DMSO): δ = 1.80 (s, 3H), 3.40 (s, 3H), 6.45 (s, 1H), 7.70–7.85 (m, 3H), 7.95 (br. s, 1H), 8.30–8.40 (m, 4H) ppm; MS (ESI+) *m*/*z*: 461.1 $[M+H]^+$; MS (ESI–) *m*/*z*: 459.2 $[M–H]^–$.

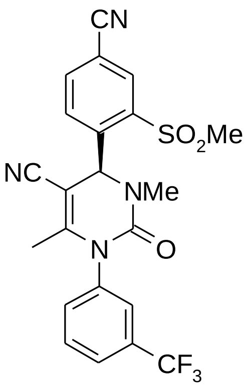

Method A: **(4*S*)-4-[4-Cyano-2-(methylsulfonyl)phenyl]-3,6-dimethyl-2-oxo-1-[3-(trifluoromethyl)phenyl]-1,2,3,4-tetrahydropyrimidine-5-carbonitrile (BAY 85-8501, 29):** The reaction was carried out under argon. **28** (75 mg, 163 μmol) was dissolved in THF (2 mL), and sodium hydride suspension in mineral oil (60%; 9.2 mg, 228 μmol) was added. After stirring at room temperature for 20 min, methyl iodide (32.4 mg, 14.2 μL, 228 μmol) was added. The reaction mixture was stirred at room temperature for 120 min. The mixture was purified directly by preparative HPLC (column: Kromasil-100A C-18, 250 mm × 4.6 mm, 5 μm; eluent: acetonitrile/H_2O + 0.1% TFA 10:90 → 80:20). Yield 18 mg (23%).

Method B: **(4*S*)-4-[4-Cyano-2-(methylsulfonyl)phenyl]-3,6-dimethyl-2-oxo-1-[3-(trifluoromethyl)phenyl]-1,2,3,4-tetrahydropyrimidine-5-carbonitrile (BAY 85-8501, 29):** The reaction was carried out under argon. **28** (460.4 mg, 1 mmol) was dissolved in absolute THF (10 mL) and cooled to –78°C. Lithium bis(tri-methylsilyl)amide solution (LiHMDS; 1 M in THF; 1 mL, 1 mmol, 1 eq.) was added dropwise. After stirring for 20 min, methyl iodide (710 mg, 5 mmol, 5 eq.) was added. The mixture was allowed to warm to room temperature over 60 h. The mixture was purified directly by preparative HPLC (column: GromSil C-18, 10 μm; eluent: aceto-nitrile/H_2O + 0.1% TFA 10:90 → 90:10). Yield 454 mg (96%); LC-MS (method 13): t_R = 1.21 min; ^{1}H NMR (400 MHz, [D_6]DMSO): δ = 1.80 (s, 3H), 2.65 (s, 3H), 3.40 (s, 3H), 6.45 (s, 1H), 7.65–8.40 (m, 6H), 8.45 (s, 1H) ppm; MS (ESI+) *m*/*z*: 475.0 (100) $[M+H]^+$; MS (ESI–) *m*/*z*: 473.2 $[M–H]^–$.

Synthesis of 30

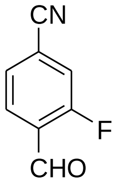

3-Fluoro-4-formylbenzonitrile (S30.1): The reaction was carried out under argon. 3-Fluoro-4-methylbenzonitrile (121 g, 895 mmol) and *N*,*N*-dimethylformamide dimethyl acetal (245 g, 2.06 mol) were dissolved in DMF (1.8 L) and the mixture was stirred under reflux overnight. The contents of the flask were then poured into H_2O (2 L), and the mixture was extracted with ethyl acetate (2 ×). The combined organic phases were washed with saturated sodium chloride solution, then concentrated, and the residue was redissolved in THF/H_2O (1:1, 2.7 L). Sodium periodate (503 g, 2.35 mol) was added and the mixture was stirred at room temperature for 1 h. The precipitate was then removed and the filtrate was recovered and extracted repeatedly with ethyl acetate. The combined organic phases were washed once with saturated sodium bicarbonate solution, dried (e.g. over sodium sulfate) and concentrated to give an oil. This oil was purified by column chromatography on silica gel (petroleum ether/dichloromethane 6:4 → 4:6 → pure dichloromethane). The product-containing fractions were concentrated. Yield 28 g (20%); GC-MS (method 11): t_R = 3.63 min;

^{1}H NMR (400 MHz, [D$_6$]DMSO): δ = 7.89 (d, 1H), 8.00 (t, 1H), 8.11 (d, 1H), 10.24 (s, 1H) ppm; MS (ESI+) *m*/*z*: 150.0 [*M*+H]$^+$.

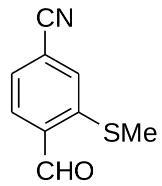

4-Formyl-3-(methylsulfanyl)benzonitrile (S30.2): S30.1 (2.00 g, 13.4 mmol) was dissolved in DMSO (27 mL) and sodium thiomethylate (1.50 g, 21.5 mmol) was added with ice-bath cooling. The mixture was stirred for 45 min and then diluted with H_2O (100 mL). The resulting precipitated product was collected by suction filtration, washed with H_2O and dried under reduced pressure. Yield 1.36 g (51%; ~35% purity); GC-MS (method 11): t_R = 5.90 min; ^{1}H NMR (400 MHz, [D$_6$]DMSO): δ = 2.54 (s, 3H), 7.78–7.86 (m, 2H), 8.08 (d, 1H), 10.18 (s, 1H) ppm; MS (ESI+) *m*/*z*: 178.0 [*M*+H]$^+$.

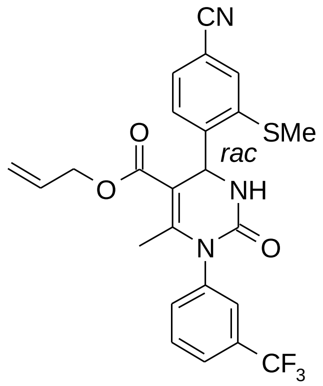

Allyl (*rac*)-4-[4-cyano-2-(methylsulfanyl)phenyl]-6-methyl-2-oxo-1-[3-(trifluoro-methyl)phenyl]-1,2,3,4-tetrahydropyrimidine-5-carboxylate (S30.3): The reaction was carried out under argon. Triethyl phosphate (1.46 g, 8.04 mmol) and phosphorus pentoxide (761 mg, 5.36 mmol) were stirred at 50°C overnight. The mixture was then diluted with MTBE (27 mL) and **S30.2** (1.18 g, 6.70 mmol), 1-[3-(trifluoromethyl)-phenyl]urea (1.37 g, 6.70 mmol) and allyl acetoacetate (1.43 g, 10.1 mmol) were added. The mixture was stirred under reflux overnight. Then, the solvent was removed under reduced pressure. The residue was suspended in diethyl ether and

collected by suction filtration. Yield 978 mg (19%, purity 63%); LC-MS (method 13): t_R = 1.37 min; ^{1}H NMR (400 MHz, [D$_6$]DMSO): δ = 2.08 (s, 3H), 2.58 (s, 3H), 4.45 (d, 2H), 4.94–5.08 (m, 2H), 5.67–5.78 (m, 1H), 5.86 (d, 1H), 7.65–7.75 (m, 4H), 7.81 (d, 3H), 8.19 (d, 1H) ppm; MS (ESI+) *m*/*z*: 488.3 [*M*+H]$^+$; MS (ESI–) *m*/*z*: 486.2 [*M*–H]$^-$.

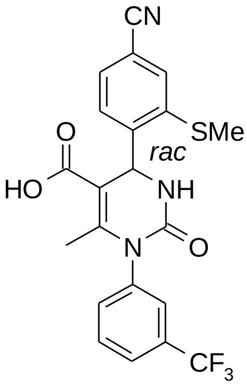

4-[4-Cyano-2-(methylsulfanyl)phenyl]-6-methyl-2-oxo-1-[3-(trifluoromethyl)phenyl]-1,2,3,4-tetrahydropyrimidine-5-carboxylic acid (S30.4): S30.3 (750 mg, 1.54 mmol) was dissolved in THF (10 mL) and morpholine (201 mg, 2.31 mmol) was added. The mixture was saturated with argon (argon was passed through the solution for 30 min). Tetrakis(triphenylphosphine)palladium(0) (7.47 mg, 0.006 mmol) was then added, and the mixture was stirred at room temperature for a further 3 h. The contents of the flask were then filtered through kieselguhr and the residue was washed with THF. The filtrate was concentrated under reduced pressure and the residue was crystallized from diethyl ether (15 mL). The crystals were collected by suction filtration and dried under high vacuum. Yield 663 mg (96%); LC-MS (method 13): t_R = 1.10 min; ^{1}H NMR (400 MHz, [D$_6$]DMSO): δ = 2.07 (s, 3H), 2.57 (s, 3H), 5.81 (d, 1H), 7.60–7.82 (m, 7H), 7.90 (d, 1H) ppm; MS (ESI+) *m*/*z*: 448.0 [*M*+H]$^+$; MS (ESI–) *m*/*z*: 446.3 [*M*–H]$^-$.

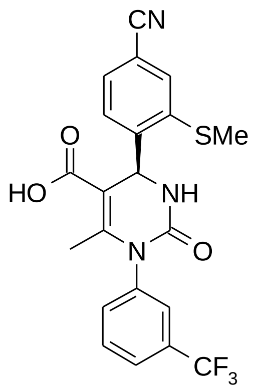

(4*S*)-4-[4-Cyano-2-(methylsulfanyl)phenyl]-6-methyl-2-oxo-1-[3-(trifluoromethyl)-phenyl]-1,2,3,4-tetrahydropyrimidine-5-carboxylic acid (S30.5): S30.4 (663 mg, 1.48 mmol) was separated into the enantiomers by preparative HPLC on a chiral phase [column: chiral silica gel phase based on the selector poly(*N*-methacryloyl-D-leucine-dicyclopropylmethylamide), 670 mm × 40 mm; sample preparation: the sample was dissolved in methanol/ethyl acetate (1:3, 20 mL); injection volume: 15 mL; gradient elution: ethyl acetate (100%) → methanol (100%); flow rate: 80 mL/min; temperature: 25°C; UV detection: 260 nm]. Yield 279 mg (84%, 96% ee); HPLC (method 4): t_R = 4.15 min; $[\alpha]_D^{20}$ = +14.0 (*c* = 0.210 in DMF); ^{1}H NMR (400 MHz, [D$_6$]DMSO): δ = 2.07 (s, 3H), 2.57 (s, 3H), 5.80 (d, 1H), 7.62–7.83 (m, 7H), 8.02 (d, 1H) ppm; MS (CI+) *m*/*z*: 448.1 [*M*+H]$^+$.

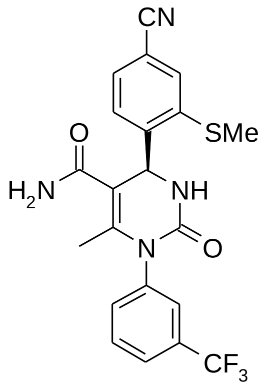

(4*S*)-4-[4-Cyano-2-(methylsulfanyl)phenyl]-6-methyl-2-oxo-1-[3-(trifluoromethyl)-phenyl]-1,2,3,4-tetrahydropyrimidine-5-carboxamide (S30.6): The reaction was carried out under argon. **S30.5** (240 mg, 0.536 mmol) was dissolved in THF (5 mL), and PyBOP (419 mg, 0.81 mmol) and TEA (380 mg, 3.76 mmol) were added. After brief stirring, the mixture was cooled to 0°C and ammonium chloride (143 mg, 2.68 mmol) was added. The reaction mixture was stirred at room temperature overnight and the contents of the flask were then added to 1 N hydrochloric acid. The

mixture was extracted with ethyl acetate (2 ×). The combined organic phases were washed with 1 N hydrochloric acid and saturated sodium chloride solution, dried over magnesium sulfate and concentrated. The residue was purified by preparative HPLC (RP column). Yield 161 mg (67%); LC-MS (method 13): t_R = 0.99 min; ^{1}H NMR (400 MHz, [D$_6$]DMSO): δ = 1.72 (s, 3H), 2.55 (s, 3H), 5.78 (s, 1H), 7.09 (br. s, 1H), 7.35 (br. s, 1H), 7.58–7.83 (m, 8H) ppm; MS (ESI+) *m*/*z*: 447.1 [*M*+H]$^+$; MS (ESI–) *m*/*z*: 445.3 [*M*–H]$^-$.

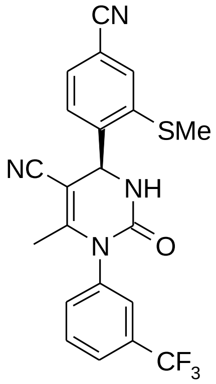

(4*S*)-4-[4-Cyano-2-(methylsulfanyl)phenyl]-6-methyl-2-oxo-1-[3-(trifluoromethyl)-phenyl]-1,2,3,4-tetrahydropyrimidine-5-carbonitrile (S30.7): The reaction was carried out under argon. **S30.6** (95 mg, 0.21 mmol) was dissolved in THF (4 mL) and (methoxycarbonylsulfamoyl)triethylammonium hydroxide (Burgess reagent; 101 mg, 0.42 mmol) was added. After stirring at room temperature for 30 min, the mixture was diluted with ethyl acetate (4 mL) and H_2O (1 mL). The mixture was then applied to a Merck EXtrelut® NT3 column and the filtrate was purified by preparative HPLC (RP-column). Yield 96 mg (quant.); HPLC (method 10): t_R = 4.61 min; ^{1}H NMR (400 MHz, [D$_6$]DMSO): δ = 1.80 (s, 3H), 2.61 (s, 3H), 5.76 (s, 1H), 7.67–7.89 (m, 7H), 8.28 (s, 1H) ppm; MS (CI+) *m*/*z*: 429.1 [*M*+H]$^+$/ *m*/*z*: 446.1 [*M*+NH$_4$]$^+$.

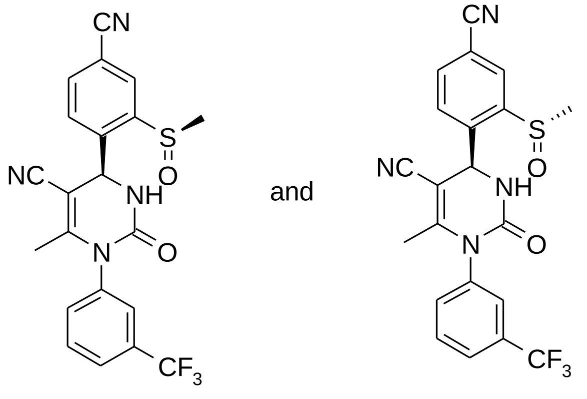

Method A: **(R_S/S_S,4*S*)-4-{4-Cyano-2-(methylsulfinyl)phenyl}-6-methyl-2-oxo-1-[3-(trifluoromethyl)phenyl]-1,2,3,4-tetrahydropyrimidine-5-carbonitrile (mixture of diastereomers) (S30.8): S30.7** (55 mg, 0.13 mmol) was dissolved in ethanol (5.5 mL), and methyltrioxorhenium(VII) (3.20 mg, 13 μmol) and hydrogen peroxide (16 mg, 0.14 mmol) were added. The reaction mixture was stirred at room temperature for 60 min and then concentrated under reduced pressure; the residue was purified by preparative HPLC (RP column). Yield 27 mg (47%); LC-MS (method 13): t_R = 1.05 min; MS (ESI+) *m*/*z*: 445.0 $[M+H]^+$.

Method B: **(R_S/S_S,4*S*)-4-{4-Cyano-2-(methylsulfinyl)phenyl}-6-methyl-2-oxo-1-[3-(trifluoromethyl)phenyl]-1,2,3,4-tetrahydropyrimidine-5-carbonitrile (mixture of diastereomers) (S30.8): S30.7** (2.00 g, 4.67 mmol) was initially charged in methanol/H_2O (4.4:1, ~40 mL); sodium periodate (1.90 g, 8.87 mmol, 1.9 eq.) was added and the mixture was stirred at 30°C for 16 h. More sodium periodate (0.45 g, 2.10 mmol, 0.45 eq.) was then added and the mixture was stirred at 50°C for a further 4 h. The reaction mixture was then added to saturated aqueous sodium bicarbonate solution (~100 mL). The mixture was extracted with ethyl acetate (4 × 50 mL), and the combined organic phases were dried over sodium sulfate, filtered and concentrated under reduced pressure. The crude product was subjected to flash chromatography on silica gel (cyclohexane → ethyl acetate). Yield 2.18 g (quant.); LC-MS (method 3): t_R = 4.61 min; ^{1}H NMR (400 MHz, [D_6]DMSO): δ = 1.85 (s, 3H), 2.85 (s, 3H), 5.75 (s, 1H), 7.70–8.50 (m, 8H) ppm; MS (ESI+) *m*/*z*: 445.0 $[M+H]^+$; MS (ESI–) *m*/*z*: 443.1 $[M-H]^-$.

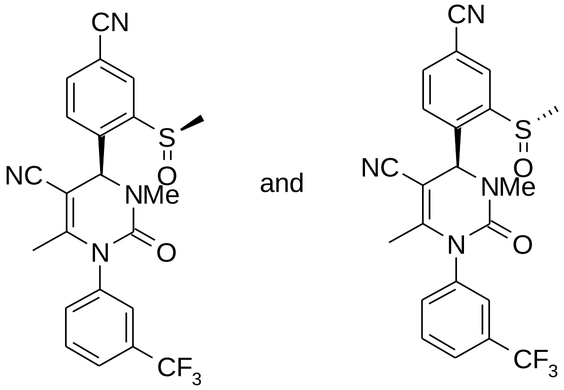

(R_S/S_S,4*S*)-4-{4-Cyano-2-(methylsulfinyl)phenyl}-3,6-dimethyl-2-oxo-1-[3-(trifluoromethyl)phenyl]-1,2,3,4-tetrahydropyrimidine-5-carbonitrile (mixture of diastereomers) (S30.9): The reaction was carried out under argon. **S30.8** (535 mg,

1.2 mmol) was initially charged in absolute THF (12 mL), and a 1 M solution of LiHMDS in THF (1.45 mL, 1.45 mmol, 1.2 eq.) was added at –78°C; methyl iodide (854 mg, 6.0 mmol, 5 eq.) was added and the mixture was stirred for 16 h, with gradual warming from –78°C to room temperature. The reaction mixture was then concentrated under reduced pressure, saturated ammonium chloride solution (50 mL) was added, and the mixture was extracted with ethyl acetate (3 × 30 mL). The combined organic phases were dried over solid sodium sulfate, filtered and concentrated under reduced pressure. The residue was purified by preparative HPLC (column: GromSil C-18, 10 μm; eluent: acetonitrile/H_2O + 0.1% TFA 10:90 → 90:10). Yield 488 mg (88%); LC-MS (method 9): t_R = 2.12 min; ^1H NMR (400 MHz, [D_6]DMSO): δ = 1.80 (s, 3H), 2.65 (s, 3H), 2.90 (s, 3H)5.80 (s, 1H), 7.70–8.20 (m, 6H), 8.45 (s, 1H) ppm; MS (ESI+) *m*/*z*: 459.0 $[M+H]^+$; MS (ESI–) *m*/*z*: 456.9 $[M–H]^–$.

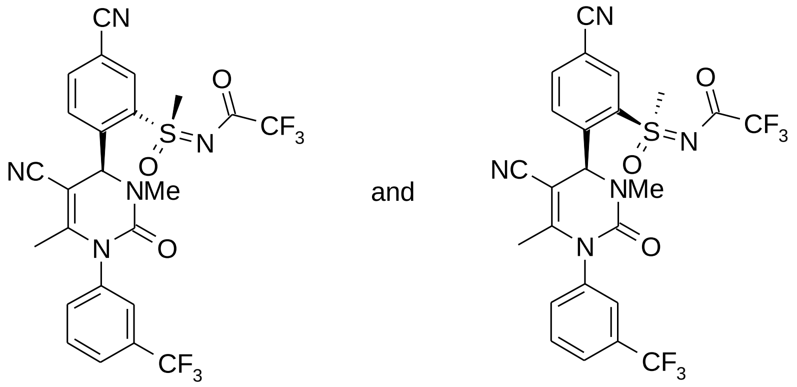

(*R*s/*S*s)-*N*-[(5-Cyano-2-{(4*S*)-5-cyano-3,6-dimethyl-2-oxo-1-[3-(trifluoromethyl)-phenyl]-1,2,3,4-tetrahydropyrimidin-4-yl}phenyl)(methyl)oxido-λ⁶-sulfanyl-idene]-2,2,2-trifluoroacetamide (mixture of diastereomers) (S30.10): The reaction was carried out under argon. The diastereomeric mixture **S30.9** (488 mg, 1.1 mmol) was initially charged in dichloromethane (10 mL), and 2,2,2-trifluoroacetamide (241 mg, 2.13 mmol, 2.0 eq.), magnesium oxide (172 mg, 4.26 mmol, 4.0 eq.), rhodium(II) acetate dimer (24 mg, 53 μmol, 0.05 eq.) and (diacetoxyiodo)benzene (514 mg, 1.60 mmol, 1.5 eq.) were added in succession. The mixture was stirred at room temperature for 16 h. More 2,2,2-trifluoroacetamide (120 mg, 1.06 mmol, 1.0 eq.), magnesium oxide (86 mg, 2.13 mmol, 2.0 eq.), rhodium(II) acetate dimer (12 mg, 27 μmol, 0.025 eq.) and (diacetoxyiodo)benzene (257 mg, 0.79 mmol, 0.75 eq.) were then added, and the mixture was stirred at room temperature for a further 24 h. The reaction mixture was then filtered through kieselguhr, the filtrate was concentrated under reduced pressure, and the residue was purified by

preparative HPLC (column: GromSil C-18, 10 μm; eluent: acetonitrile/H_2O + 0.1% TFA 10:90 → 80:20). Yield 160 mg (25%); LC-MS (method 13): t_R = 2.12 min; MS (ESI+) *m*/*z*: 570.1 $[M+H]^+$; MS (ESI–) *m*/*z*: 567.9 $[M–H]^–$.

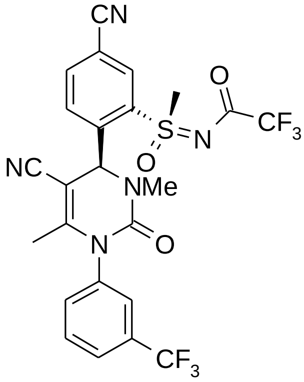

(*R*S)-*N*-[(5-Cyano-2-{(4*S*)-5-cyano-3,6-dimethyl-2-oxo-1-[3-(trifluoromethyl)-phenyl]-1,2,3,4-tetrahydropyrimidin-4-yl}phenyl)(methyl)oxido-λ^6-sulfanyl-idene]-2,2,2-trifluoroacetamide (S30.11): The diastereomeric mixture of **S30.10** (160 mg) was separated by flash chromatography on silica gel (cyclohexane → cyclohexane/ethyl acetate 45:55). The *R*S-diastereomer was obtained in later-eluting fractions. Yield 68 mg; LC-MS (method 13): t_R = 1.35 min; MS (ESI+) *m*/*z*: 570.1 $[M+H]^+$; MS (ESI–) *m*/*z*: 568.4 $[M–H]^–$.

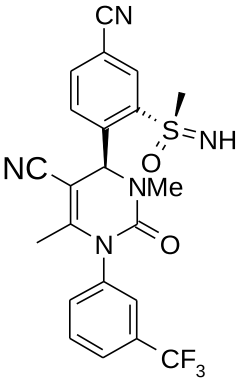

(*R*S)-(4*S*)-4-[4-Cyano-2-(*S*-methylsulfonimidoyl)phenyl]-3,6-dimethyl-2-oxo-1-[3-(trifluoromethyl)phenyl]-1,2,3,4-tetrahydropyrimidine-5-carbonitrile (30): The reaction was carried out under argon. **S30.11** (78 mg, 137 μmol) was initially charged in an acetonitrile/methanol mixture (10:1, 7.7 mL). At 0°C, solid potassium carbonate (9.5 mg, 68 μmol, 0.5 eq.) was added and the reaction mixture was stirred for 15 min. The mixture was then neutralized with TFA (7.8 mg, 68 μmol, 0.5 eq.) and concentrated under reduced pressure. The residue was purified by preparative HPLC

(column: GromSil C-18, 10 µm; eluent: acetonitrile/H_2O + 0.1% TFA 10:90 → 80:20). Yield 60 mg (93%); LC-MS (method 12): t_R = 0.98 min; chiral analytical HPLC (column: Chiralpak AD-H, 250 mm × 4.6 mm, 5 µm; eluent: isohexane/ethanol 50:50; flow rate: 1 mL/min; injection volume: 10 µL; temperature: 40°C; UV detection: 220 nm): t_R = 4.40 min; $[\alpha]_D^{20}$ = –286.9 (*c* = 0.49 in chloroform); ^{1}H NMR (400 MHz, [D_6]DMSO): δ = 1.80 (s, 3H), 2.70 (s, 3H), 3.30 (s, 3H), 6.80 (s, 1H), 7.70–8.30 (m, 6H), 8.45 (s, 1H) ppm; MS (ESI+) *m*/*z*: 474.3 $[M+H]^+$; MS (ESI–) *m*/*z*: 472.4 $[M–H]^–$

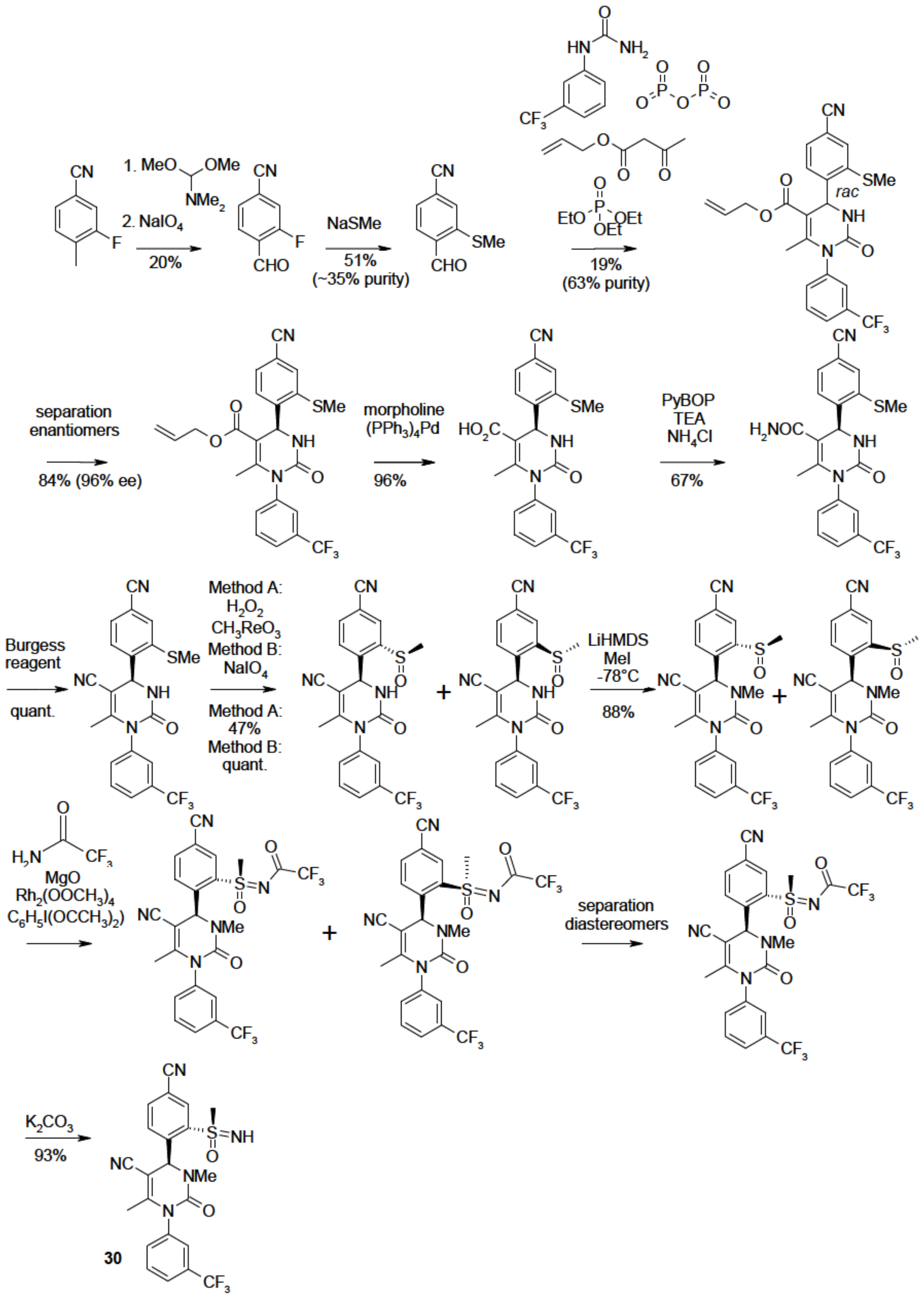

Scheme 6. Synthesis of compound **30**

EXPERIMENTAL BIOLOGY

All procedures conformed to European Community directives and national legislation (German law for the protection of animals) for the use of animals for scientific purposes and were approved by the competent regional authority.

Protein for Biochemical Neutrophil Elastase Assays: Human neutrophil elastase (HNE) enzyme purified from blood neutrophils was purchased from SERVA Electrophoresis GmbH, Heidelberg, Germany. The orthologous rat enzyme, rat neutrophil elastase (RNE), was prepared from lungs as a source of neutrophil cells; here, the rat lung tissue was mechanically homogenized and neutrophil elastase was liberated from the neutrophil granula via acid extraction and enriched via fractionated ammonium sulfate precipitation. Murine neutrophil elastase (MNE) was prepared from neutrophil cells, harvested from a peritoneal lavage after lipopolysaccharide (LPS) challenge, as follows: Cells in homogenate or lavage were collected in Dulbecco's phosphate-buffered saline (PBS) supplemented with 10 mM EDTA. Cells were harvested via centrifugation (5 min, 14000 rpm) and transferred to ice-cold water with a resulting cell number of 3×10^6 cells/mL. Cells were lysed via ultrasonication until the sample was milky white and homogeneous (e.g., 2 × 30 sec pulses with intermittent cooling on ice). Neutrophil granula were lysed via sulfuric acid treatment (adding 0.4 N sulfuric acid to give a final concentration of 0.16 N, incubation on ice for 30 min with mixing). The sample was transferred to siliconized Eppendorf tubes and the soluble fraction was harvested via centrifugation (5 min, 14000 rpm). The supernatant with the liberated neutrophil elastase was dialyzed against 20 mM sodium acetate pH 4.0 at 4°C, centrifuged again and the final supernatant was stored at 4°C.

Elastase Inhibition Assessment: Elastase inhibition was assessed in vitro by applying microtiter-based biochemical test systems. In general, the hydrolysis of a fluorogenic peptide substrate, MeOSuc-AAPV-AMC (24 µL, final concentration 1 mM–1 µM, routinely 20 µM) in assay buffer (0.1 mM HEPES pH 7.4, 0.5 M NaCl, 0.1% BSA), at 37°C, in the presence or absence of test compound (1 µL) in DMSO, was assessed by applying neutrophil elastase (25 µL, final concentration 16–0.08 nM, routinely 0.8 nM) in assay buffer over a time period with linear enzyme kinetics (minutes to hours, routinely 60 minutes). Inhibitory capacity of very potent inhibitors (subnanomolar IC_{50} under routine conditions) was assessed with a matching very low

enzyme concentration (0.08 nM) in order to maintain a good sensitivity of the assay. Here, the linear enzyme reaction was recorded up to several hours to achieve a sufficient turnover of the substrate. The increase in fluorescence (ex. 395 nm, em. 460 nm) was proportional to elastase activity. IC_{50} values were determined by inter-polation from plots of relative fluorescence versus inhibitor concentration (N≥4, standard deviation in general was less than factor 2). K_i values were determined from Dixon plots (Figures S6 and S7).

We observed a good correlation between IC_{50} values and K_i values as measurements were carried out at substrate concentration below k_m.

For selected compounds, this assay was performed using a modified assay buffer (assay buffer with 1 mM H_2O_2) to mimic oxidative stress conditions.

The on-rates at which elastase inhibitors bind to the target were determined by applying a modified enzymatic assay. The hydrolysis of the fluorogenic substrate MeOSuc-AAPV-umbelliferyl was monitored on a millisecond timescale, in the presence or absence of elastase inhibitor. Using nonlinear regression of the reaction progress curves, the observed rate constant of the onset of inhibition, k_{obs} was obtained by fitting the following equation

$$\mathrm{P}(t) = v_s t + (v_o - v_s)(1 - \mathrm{e}^{-k_{obs}\ t})/k_{obs}$$

where P(t) is the product concentration at reaction time t (measured in relative fluorescent units), v_o is the initial velocity and v_s is the velocity at steady state.[10]

k_{obs} values were then plotted against elastase inhibitor concentration. The slope of the linear correlation

$k_{obs} = k_{off} + k_{on}$ [inhibitor]

gave the association rate constant, k_{on} at the substrate concentration used.

The estimated off-rate at which elastase inhibitors dissociate from the target, k_{off} was calculated from the corresponding K_i values and on-rates using the equation

$k_{off} = k_{on}\ K_i$

The corresponding residence times for the elastase inhibitor binding were calculated using the equation

$t_{1/2} = 1/k_{off}$

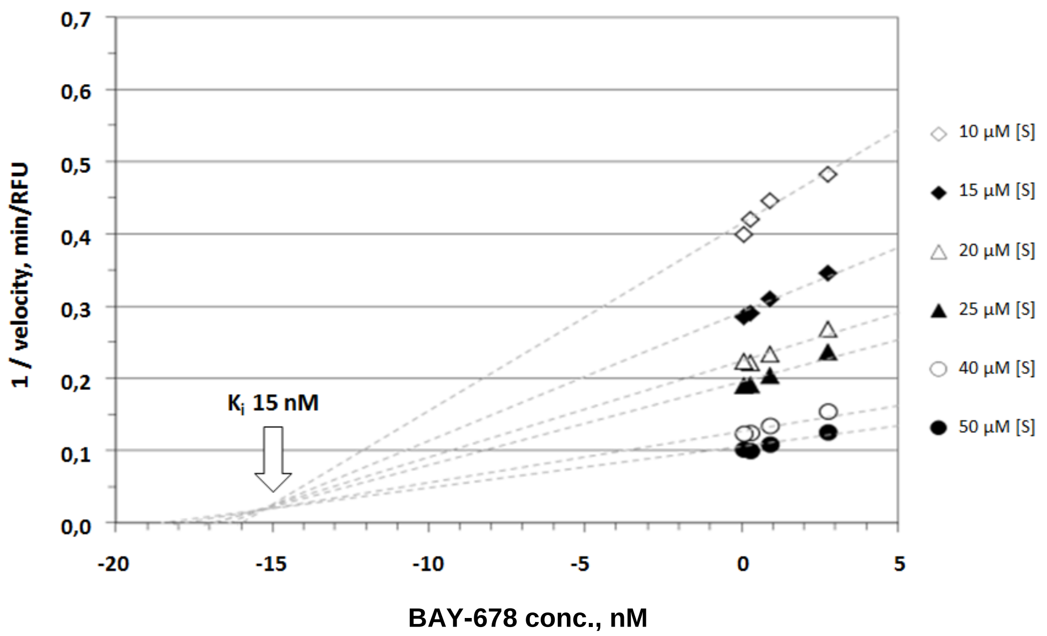

Figure S6. *K*ᵢ determination via the Dixon plot of BAY-678 (**20**).

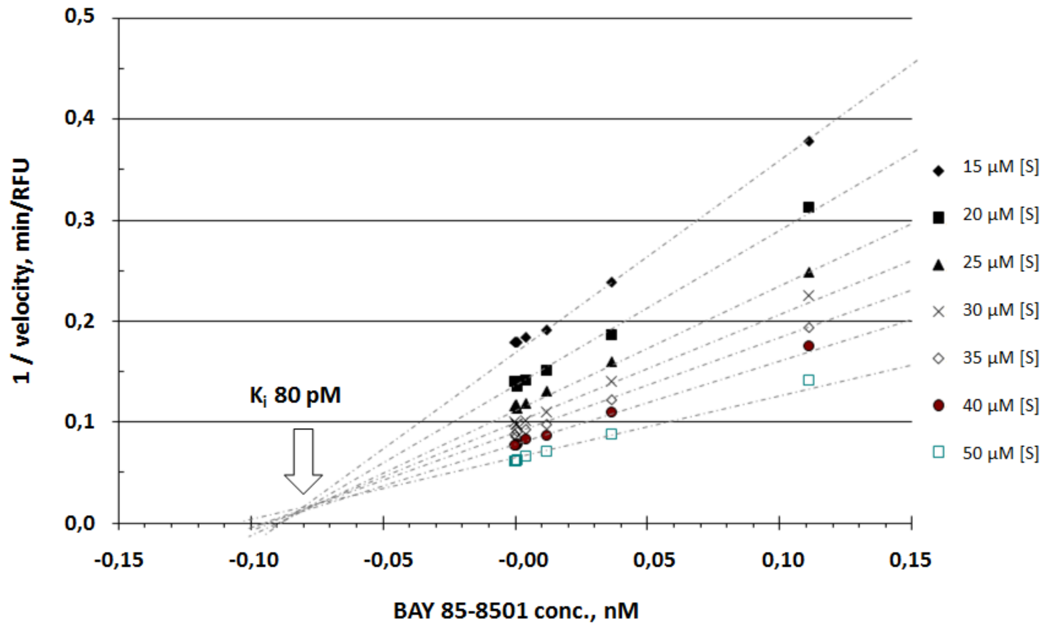

Figure S7. *K*ᵢ determination via the Dixon plot of BAY 85-8501 (**29**).

Table S3. In vitro potency of BAY-678 and BAY 85-8501 against 21 serine proteases.

Serine Protease	BAY-678 and BAY 85-8501 IC_{50} [nM]
PPE	>30000
Cathepsin G	>30000
Chymotrypsin	>30000
Trypsin	>30000
Chymase	>30000
DPPII	>30000
DPPIV	>30000
Urokinase	>30000
FAP	>30000
Kallikrein-1	>30000
Kallikrein-4	>30000
Kallikrein-5	>30000
Kallikrein-7	>30000
Kallikrein-12	>30000
Kallikrein-B1	>30000
Thrombin	>30000
FXa	>30000
FVIIa	>30000
FIXa	>30000
FXIa	>30000
Plasmin	>30000

EXPERIMENTAL PHARMACOLOGY

In Vivo Pharmacodynamic Studies – Acute Lung Injury Mice Model: Elastase-induced lung failure in mice, rats or hamsters is a widely used animal model of acute lung failure (also: ‘acute lung injury’, ‘acute respiratory distress syndrome’).[11,12] The animals were treated 1 hour prior to orotracheal instillation of human neutrophil elastase (HNE) or porcine pancreatic elastase (PPE). In this protocol, each mouse received an intratracheal instillation of 1.2 U HNE or 0.5 U PPE dissolved in 100 μL of sterile isotonic saline. 1 hours after orotracheal HNE or PPE instillation, a broncho-alveolar lavage was carried out, and the hemoglobin content and the differential cell picture of the lavage were determined.

EXPERIMENTAL PHARMACOKINETICS

Pharmacokinetic Studies: Pharmacokinetic properties in rats were determined by administering the test compounds as solutions orally by gavage (doses between 0.3

– 3 mg/kg // Water 50% + Solutol 40% + EtOH 10% or Water 50% + PEG400 40% + EtOH 10% // admin volume 5 mL/kg) or intravenously by infusion (15 min) (dose 0.84 mg/kg // formulation: Plasma 99% + DMSO 1% // admin volume 5 mL/kg) of the compound into a lateral tail vein. One day before the administration of the test compound, a catheter was implanted into a jugular vein. Blood samples were withdrawn from the jugular vein via the catheter at different time points, transferred to heparinized tubes and plasma was separated by centrifugation. The samples were analyzed by LC-MS/MS (e.g., using an API 3000 system, Sciex Corp. Toronto, Canada). PK parameters were calculated using in-house PK software (KinEx).

CYP Inhibition Assay: The ability of substances to inhibit CYP1A2, CYP2C9, CYP2D6 and CYP3A4 in humans was investigated with pooled human liver microsomes as enzyme source in the presence of standard substrates (see below) which form CYP-specific metabolites. The inhibitory effects were investigated with six different concentrations of the test compounds [1.6, 3.1, 6.3, 12.5, 25 and 50 µM], compared with the extent of the CYP-specific metabolite formation with the standard substrates in the absence of the test compounds, and the corresponding IC_{50} values were calculated. Known inhibitors of the respective enzymes (‘standard inhibitors’) were included as positive controls. *Procedure:* Test compounds were preferably dissolved in acetonitrile. 96-well plates were incubated with pooled human liver microsomes at 37°C for a defined time (10–20 min). Incubation of phenacetin (CYP1A2), diclofenac (CYP2C9), dextromethorphan (CYP2D6) or midazolam (CYP3A4) at their reaction K_m values with human liver microsomes in the presence of different concentrations of the test compound (as potential inhibitor) was carried out on a workstation (Tecan, Genesis, Crailsheim, Germany). Substrates (with 2% acetonitrile) in the absence of the test compound were incubated in parallel as references. Standard incubation mixtures consisted of 1.3 mM NADP, 3.3 mM $MgCl_2 \cdot 6H_2O$, 3.3 mM glucose 6-phosphate, glucose-6-phosphate dehydrogenase (0.4 U/mL) and 100 mM phosphate buffer (pH 7.4) in a total volume of 200 µL. Total solvent (acetonitrile) concentration was 2%. Reactions were stopped by addition of 100 µL acetonitrile containing the respective internal standard. Precipitated proteins were removed by centrifugation of the well plate (3000 rpm, 10 min); supernatants were combined and analyses were performed by LC-MS/MS (e.g. API 3000 or API 4000 system, Sciex Corp. Toronto, Canada).

Hepatocyte Assay To Determine the Metabolic Stability: The metabolic stability of test compounds in the presence of hepatocytes was determined by incubating the compounds with low concentrations (preferably below or around 1 μM) and with low cell counts (preferably 1 × 10^6 cells/mL) in order to ensure, as far as possible, linear kinetic conditions in the experiment. Seven samples of the incubation solution were taken in a fixed time pattern for LC-MS/MS analysis (e.g. API 2000 system, Sciex Corp., Toronto, Canada) in order to determine the half-life (i.e., the degradation) of the compound in each case. Various clearance parameters (CL) and F_{max} values were calculated from this half-life (see below). The CL and F_{max} values represent a measure of the phase 1 and phase 2 metabolism of the compounds in the hepatocytes. In order to minimize the influence of the organic solvent on the enzymes in the incubation mixtures, this concentration was generally limited to 1% (acetonitrile) or 0.1% (DMSO). A cell count for hepatocytes in the liver of 1.1 * 10^8 cells/g of liver was used for calculation for all species and breeds. CL parameters calculated on the basis of half-lives extending substantially beyond the incubation time (normally 90 min) can only be regarded as rough guidelines.

The calculated parameters (and their meaning) were:

**F_{max} well-stirred [%]**	maximum possible bioavailability after oral administration
Calculation:	(1-CL_{blood} well-stirred/QH) * 100<br>with QH = species-specific hepatic blood flow
**CL_{blood} well-stirred [L/(h*kg)]**	calculated blood clearance (well-stirred model)
Calculation:	(QH * $CL'_{intrinsic}$) / (QH + $CL'_{intrinsic}$)
**$CL'_{intrinsic}$ [mL/(min*kg)]**	maximum ability of the liver (of the hepatocytes) to metabolize a compound (on the assumption that the hepatic blood flow was not rate-limiting)
Calculation:	$CL'_{intrinsic, apparent}$ * species-specific hepatocyte count [1.1 × 10^8/g of liver] * species-specific liver weight [g/kg]
**$CL'_{intrinsic, apparent}$ [mL/(min*mg)]**	normalizes the elimination constant by dividing it by the hepatocyte cell count x (x × 10^6/mL) employed
Calculation:	k_{el} [1/min]/(cell count [x × 10^6]/incubation volumes [mL])

[1] W. Kabsch, *Acta Crystallogr. Sect. D* **2010**, *66*, 125–132.

[2] M. D. Winn, C. C. Ballard, K. D. Cowtan, E. J. Dodson, P. Emsley, P. R. Evans, R. M. Keegan, E. B. Krissinel, A. G. Leslie, A. McCoy, S. J. McNicholas, G. N. Murshudov, N. S. Pannu, E. A. Potterton, H. R. Powell, R. J. Read, A. Vagin, K. S. Wilson, *Acta. Crystallogr. Sect. D* **2011,** *67*, 235–242.

[3] T. G. G: Battye, L. Kontogiannis, O. Johnson, H. R. Powell, A. G. Leslie, *Acta. Crystallogr. Sect. D* **2011,** 67, 271-281.

[4] PROTEUM2, Version 2009, Bruker, AXS Inc., Madison, Wisconsin, USA, **2009**.

[5] G. M. Sheldrick, *Acta Crystallogr*. *Sect. A* **2008**, *64*, 112–122.

[6] Oxford Diffraction, *CrysAlis CCD*, Oxford Diffraction Ltd, Abingdon, England, **2006**.

[7] F. Bigi, S. Carloni, L. Ferrari, R. Maggi, A. Mazzacani, G. Sartori, *Tetrahedron Lett.* **2001**, *42*, 5203–5205.

[8] D. Arlt, B. Bömer, R. Grosser, W. Lange, *Angew. Chem. Int. Ed.* **1991**, *30*, 1662–1664; *Angew. Chem.* **1991**, *103*, 1685–1687.

[9] Freshly prepared, according to the procedure of M. P. Cava, M. V. Lakshmikantham, M. J. Mitchell, *J. Org. Chem.* **1969**, *34*, 2665–2667.

[10] J. F. Morrison, C. T. Walsh, *Adv. Enzymol. Relat. Areas Mol. Biol.* **1988**, *61*, 201–301.

[11] G. M. Tremblay, E. Vachon, C. Larouche, Y. Bourbonnais, *Chest* **2002**, *121*, 582–588.

[12] T. Kuraki, M. Ishibashi, M. Takayama, M. Shiraishi, M. Yoshida, *Am. J. Respir. Crit. Care Med.* **2002**, *166*, 496–500.
